# Copper-catalyzed reaction of aziridine for the synthesis of substituted imidazolidine and imidazolidinone

**DOI:** 10.3389/fchem.2023.1272034

**Published:** 2023-09-29

**Authors:** Kota Hashimoto, Daiki Higuchi, Satoshi Matsubara, Kei Murakami

**Affiliations:** ^1^ Department of Chemistry, School of Science, Kwansei Gakuin University, Sanda, Japan; ^2^ JST-PRESTO, Chiyoda, Japan

**Keywords:** copper catalyst, aziridine, imine, isocyanate, imidazolidine, imidazolidinone, cyclization

## Abstract

Herein we report a copper-catalyzed synthesis of imidazolidine by employing the reaction of aziridine with imine. The reaction smoothly provided a diverse range of 2-substituted imidazolidines with high compatibility with various functional groups. Moreover, during our investigation, we discovered that isocyanate also reacted with aziridine to yield substituted imidazolidinones efficiently. The versatility of these reactions was further demonstrated by their application in the synthesis of hybrid molecules derived from two pharmaceutical compounds. This approach opens new possibilities for the discovery of novel classes of bioactive molecules.

## 1 Introduction

**FIGURE 1:**
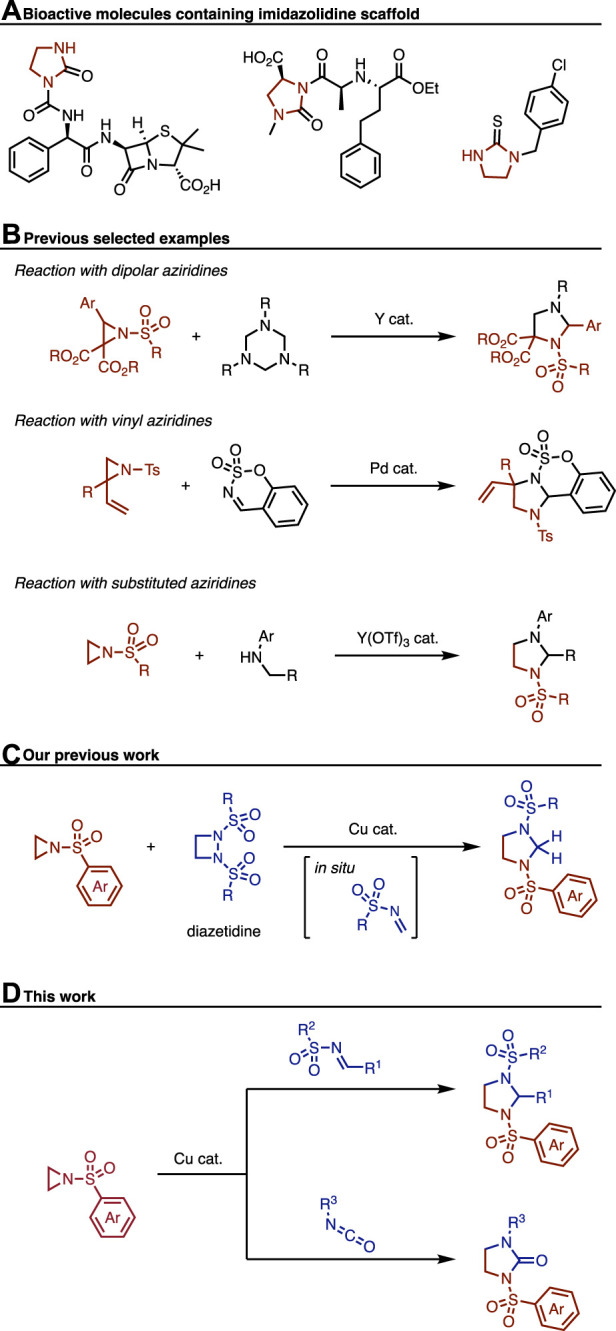
Background of this research.

The construction of saturated nitrogen-containing heterocycles is of utmost importance as they are prevalent structures in a wide range of natural products and pharmaceuticals (Smith et al., 1999; Antonopoulos et al., 2008). Among such saturated azaheterocycles, the synthesis of substituted imidazolidines has received comparatively less attention than other mono-azaheterocycles, such as pyrrolidines. However, given that imidazolidine scaffolds have been identified in numerous bioactive molecules, the development of new synthetic methodologies for accessing these scaffolds is highly important (Figure 1A).

To access imidazolidine derivatives, aziridines have been widely employed as the starting materials (Figure 1B). Aziridines can be categorized into three types based on their reactivity: i) activated dipolar aziridines (Wu and Zhang, 2012; Shi et al., 2021), ii) activated vinyl aziridines (Lin et al., 2018; Spielmann et al., 2018), and iii) other substituted aziridines (Liu et al., 2017; Sengoden et al., 2017; Li et al., 2018; Tu et al., 2019; Pradhan et al., 2020). For type i), Shi et al. reported in 2021 the reaction of donor-accepter-type aziridines with 1,3,5-triazinanes through a [3 + 2] cycloaddition (Shi et al., 2021). This reaction demonstrated that aziridines can serve as efficient precursors for the synthesis of multi-substituted imidazolidines. Regarding type ii), Spielmann et al. reported the palladium-catalyzed [3 + 2] cycloaddition reaction of vinylaziridines with cyclic imines (Spielmann et al., 2018). The reaction proceeds through the formation of π-allylpalladium intermediate, which then reacts with the cyclic imine to yield the corresponding products. This approach allows for the diastereoselective synthesis of imidazolidine-fused tricyclic molecules. For type iii), Sengoden et al. reported copper-catalyzed reaction of aziridine with *N*-alkylanilines (Sengoden et al., 2017). This reaction involves the oxidation of aniline derivatives to generate an iminium intermediate *in situ*, which allows the efficient preparation of *N-*aryl-*N*-sulfonylimidazolidines. Despite the availability of various methods for accessing imidazolidines from aziridines, copper-catalyzed reactions of aziridines with imines or isocyanates have remained relatively underdeveloped (Zhu and Chiba, 2016; Bozorov et al., 2019; Pal et al., 2020; Pineschi, 2020; Zhang et al., 2022).

In this context, our previous study demonstrated the reaction of aziridine with diazetidine (Figure 1C), which led to the formation of imidazolidines (Higuchi et al., 2023). While the scope of this reaction was broad to construct various imidazolidines, the synthetic limitation associated with diazetidine hindered its widespread use for accessing imidazolidine derivatives. During our mechanistic investigation, we disclosed that an *in situ* formed imine reacted with aziridine to form the corresponding imidazolidine. This discovery motivated us to explore the reaction of aziridines with substituted imines to expand the scope of this transformation. Herein, we report the copper-catalyzed synthesis of imidazolidines through the reaction of aziridines with imines (Figure 1D). This transformation exhibits good functional group compatibility to afford the desired products efficiently. Additionally, we have successfully demonstrated that the use of isocyanates instead of imines allows accessing imidazolidinones. We have shown its application in the synthesis of hybrid molecules derived from Celecoxib and NLRP3 inflammasome inhibitor.

## 2 Results and discussions

**FIGURE 2:**
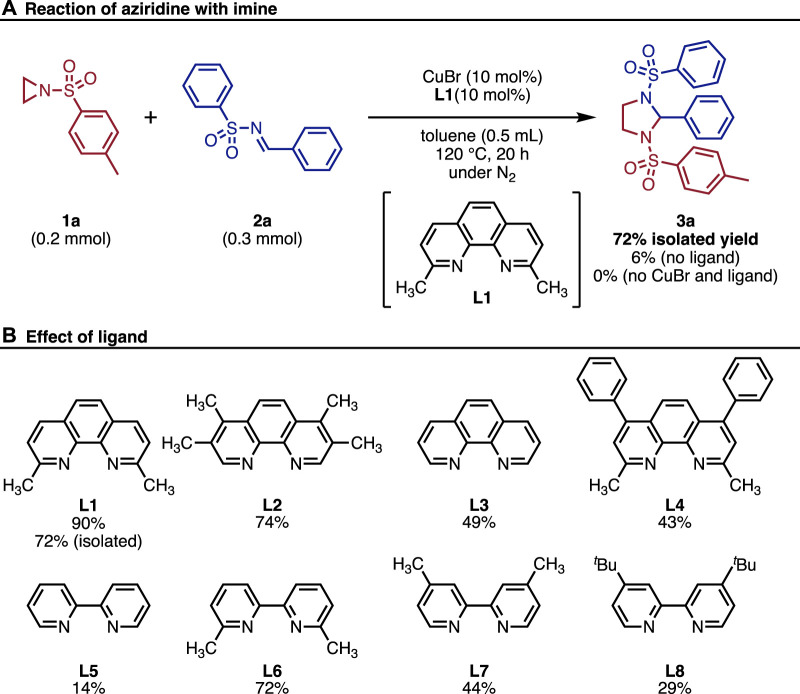
**(A)** Reaction of aziridine with imine. **(B)** Effect of ligand. ^1^H NMR yields were shown using 1,1,2,2-tetrachloroethane.

We initiated our study by investigating the ligand effect for the reaction. The treatment of aziridine **1a** with imine **2a** in the presence of 10 mol% of CuBr and **L1** in toluene at 120 °C for 20 h afforded the corresponding imidazolidine **3a** in 72% isolated yield (Figure 2A). It is noteworthy that the ligand effect is critical where 6% of **3a** was obtained without the addition of **L1**. Furthermore, CuBr was found to be essential for the reaction, and no product was observed in the absence of both CuBr and **L1**. We then investigated the effect of ligands (Figure 2B). Although no clear correlation between ligand structure and catalytic activity was observed, 1,10-phenanthroline derivatives showed good results (^1^H NMR yields using various ligands **L2**: 74% **L3**: 49%, **L4**: 43%). Similarly, 2,2-bipyridine derivatives also promoted the reaction (**L5**–**L8**: 14%–72% ^1^H NMR yields.). After the screening, we decided to use **L1** as the optimal ligand because it provided the highest isolated yield.

**FIGURE 3:**
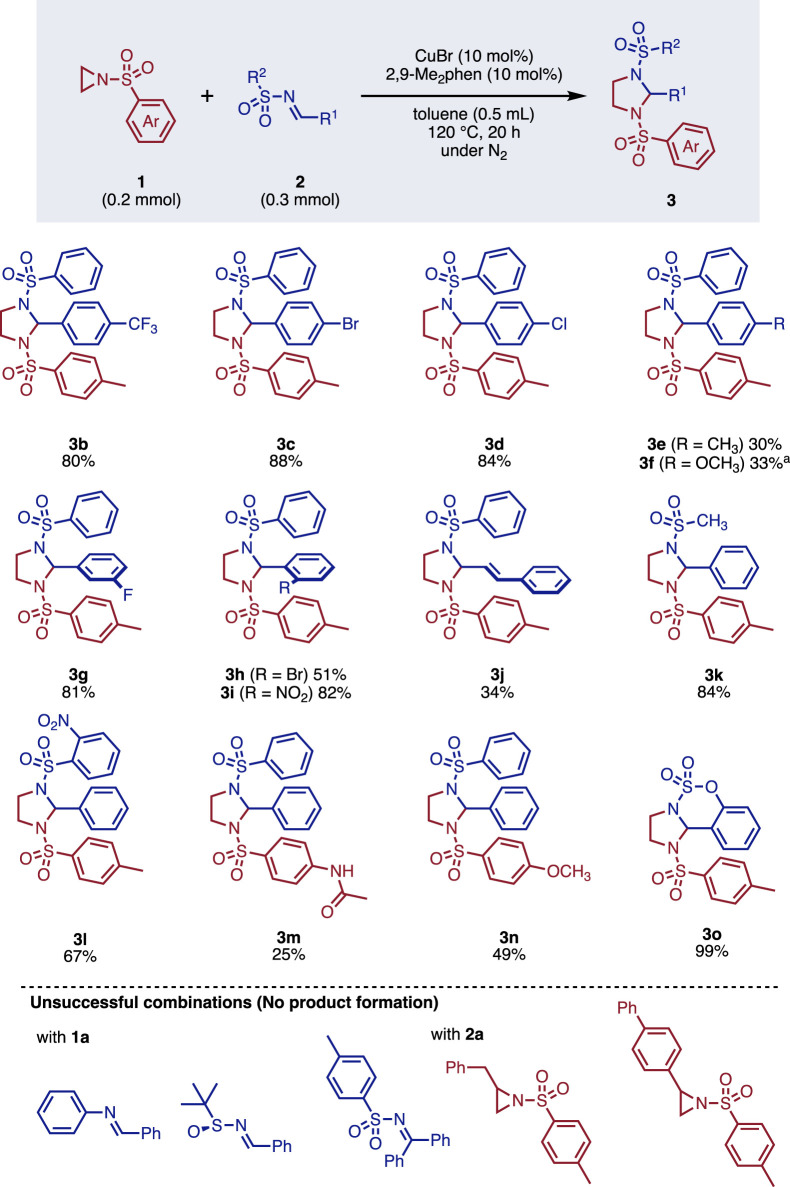
Scope of imidazolidine synthesis. ^a^ reaction time: 40 h.

The scope of the reaction was investigated (Figure 3). Initially, we examined the substituent effect on imines. The use of electron-withdrawing *p*-trifluoromethylphenyl group at the R^1^ position allowed an efficient transformation to give **3b** in 80% yield. Other electron-withdrawing groups, such as *p*-bromo- or *p*-chlorophenyl groups, provided the corresponding products **3c** and **3d** in 88% and 84% yields, respectively. It is noteworthy that such halogen moieties can serve as footholds for further transformation. The reactions of electron-donating *p*-methyl- or *p*-methoxyphenyl-substituted imines were relatively sluggish, however, both starting materials were completely consumed. Not only *p-*substituted one but also *m-*substituted phenylsulfonylimine reacted smoothly converted to **3g** in 81% yield. Despite the bulky *o*-bromo-, or *o*-nitrophenyl groups, the corresponding products **3h** and **3i** were obtained in moderate to high yields. Although the yield was not high, imine **2j** derived from cinnamaldehyde, was applicable to the reaction to afford **3j** in 34% yield. A variety of sulfonyl groups were tolerated under the reaction conditions, where methylsulfonyl (Ms) or *o*-nitrophenylsulfonyl (Ns)-substituted imines provided **3k** and **3l** in 84% and 67% yields without the loss of these sulfonyl groups. When electron-donating group-substituted arylsulfonylaziridines were employed, the reactions were less effective and gave **3m** and **3n** in 25% and 49% yields, respectively. Furthermore, the reaction was applicable to cyclic imine to give **3o** in almost quantitative yield. Unsuccessful combinations of substrates are summarized at the bottom of Figure 3. The reactions of *N*-phenylimine or *N*-sulfoxyimine with **1a** failed to give the corresponding products. Although the reaction of unsubstituted aziridine **1a** with imine **2a** smoothly provided the products, the reaction of diphenylmethanimine or substituted aziridine failed. This can be attributed to steric hindrance, as the reaction of substituted aziridine proceeded smoothly with methanimine derivatives (Higuchi et al., 2023).

**FIGURE 4:**
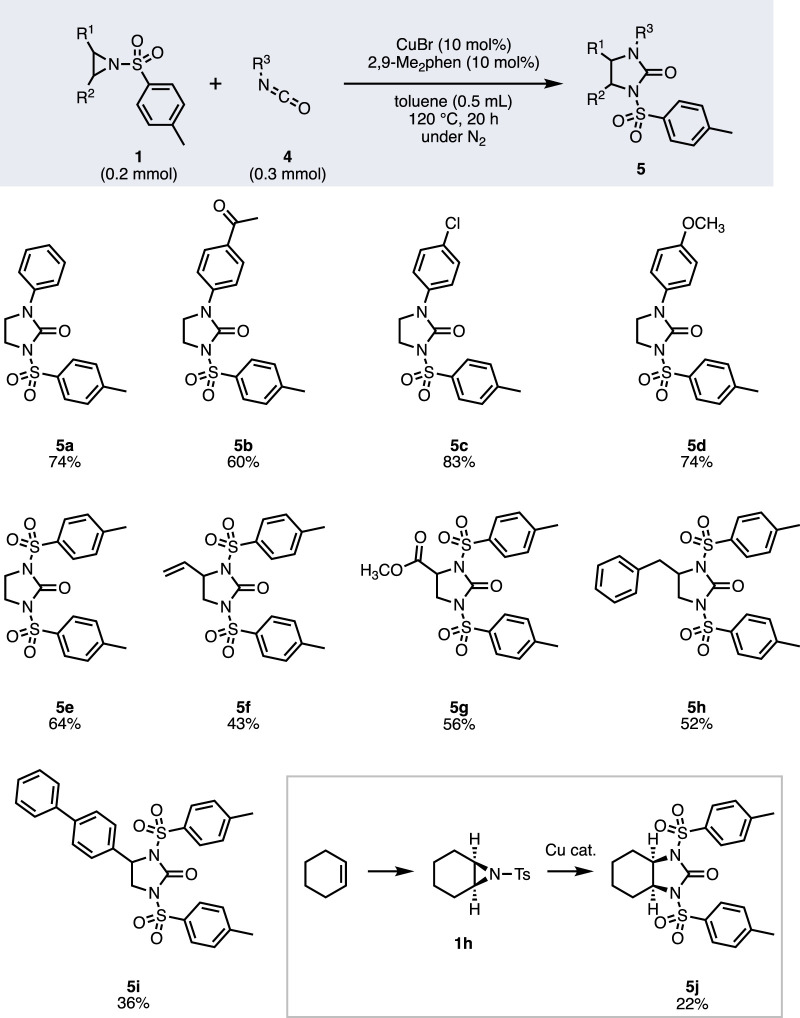
Scope of imidazolidinone synthesis.

To expand the scope of the reaction, we employed isocyanate **4** as the coupling partner (Figure 4). The reaction of tosylaziridine **1a** with phenylisocyanate **4a** under the same optimized conditions afforded imidazolidinone **5a** in 74% yield. A variety of arylisocyanate was applicable to the reaction where electron-withdrawing *p*-acetyl-, *p*-chloro-, or electron-donating *p-*methoxyphenylisocyanate gave products **5b**–**5d** in 60%–83% yields. When tosylisocyanate was employed as the substrate, product **5e** was obtained in 64% yield. We then investigated the scope of substituted aziridines. Compared with the reaction with imine, the reaction with isocyanate showed a broader substrate scope of aziridines. The reaction of vinylaziridine afforded **5f** in 43% yield, and no isolable byproduct that reacted with the olefin was observed, leaving less than 20% of vinylaziridine remaining. Other substituents, such as methoxycarbonyl, benzyl, or biphenyl groups, were applicable to the reaction to give **5g**–**5i** in 36%–56% yields. Bicyclic aziridine **1h**, which can be prepared from cyclohexene, reacted to give **5j** in 22% yield. Although the yield was not high, the formal *cis*-diamination product can be obtained through this transformation (Minakata et al., 2021).

**FIGURE 5:**
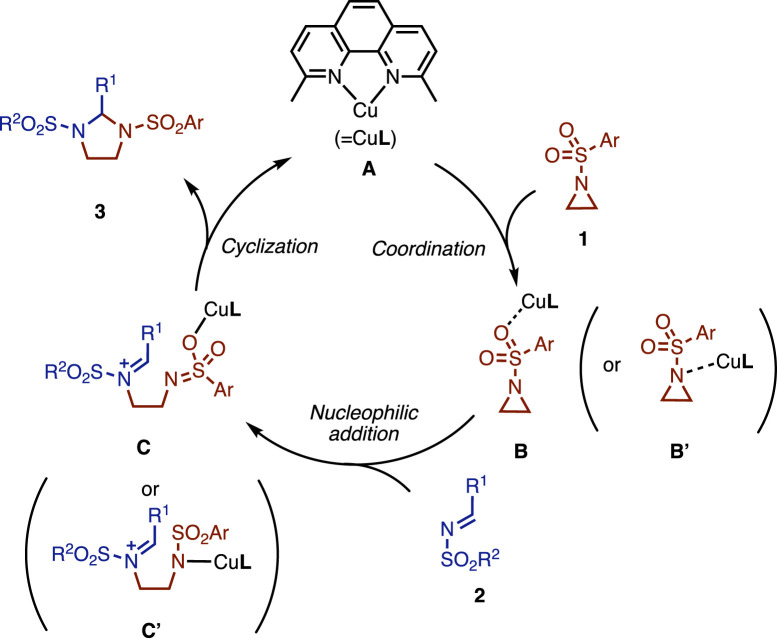
Proposed mechanism.

A proposed mechanism is shown in Figure 5. The reaction initiates with the coordination of aziridine **1** to copper catalyst **A** to give the corresponding intermediate **B** (or **B′**). Subsequently, imine **2** attacks to open the aziridine ring to give intermediate **C** (or **C’**). Finally, a cyclization reaction takes place from **C** to give product **3**. From the result of the competitive experiment, the electron-donating group might reduce the reactivity of intermediate **C**, which hampers the cyclization and eventually the turnover of the catalyst. Note that another reaction mechanism can be depicted (see Supplementary Figure S1). At this stage, we considered that the reaction with isocyanate also proceeded through a similar reaction mechanism. Because it is difficult to elucidate the full reaction mechanism, further investigation is ongoing.

**FIGURE 6:**
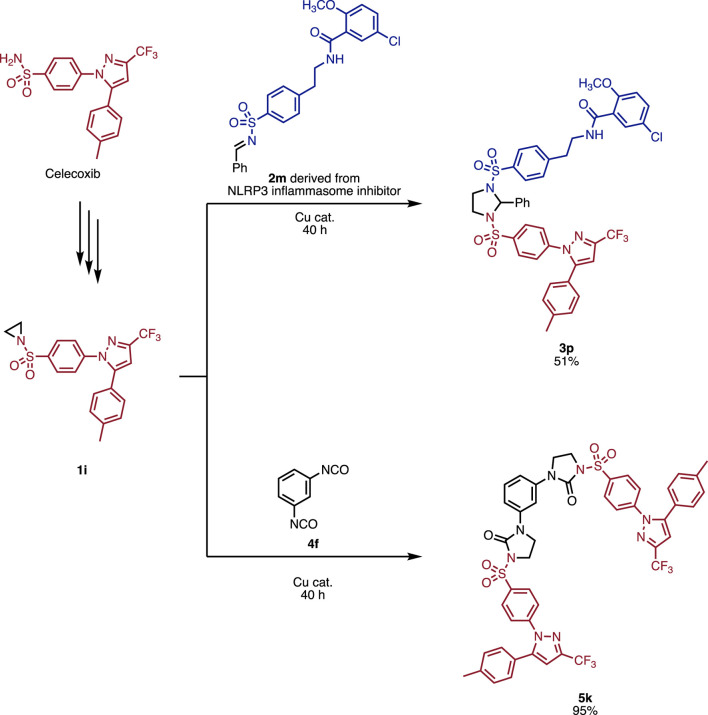
Application toward the syntheses of pharmaceutical hybrids.

Finally, we applied the developed reaction to synthesize pharmaceutical hybrids (Figure 6). Firstly, we synthesized aziridine **1i** from Celecoxib (Ozaki et al., 2021). Aziridine **1i** was then reacted with imine **2m**, derived from an NLRP3 inflammasome inhibitor, to give **3p** in 51% yield. Furthermore, the reaction of aziridine **1i** with bis-isocyanate **4f** afforded product **5k** in 95% yield. These applications clearly demonstrate the excellent functional group compatibility that allows connecting various pharmaceuticals.

## 3 Conclusion

In conclusion, we have successfully reported the copper-catalyzed reaction of aziridine with imine and isocyanate, which afforded imidazolidine and imidazolidinone respectively. These developed reactions exhibited broad functional group compatibility to access a diverse array of potential bioactive 5-membered azaheterocycles. Furthermore, to demonstrate the practical applicability of these reactions, we prepared hybrids of pharmaceutical molecules using the developed reactions. These reactions surely facilitate the syntheses of complex azaheterocycles, which will accelerate the discovery of structurally new bioactive molecules in the future.

## 4 Materials and methods

For experimental procedures and compound characterization data, see the Supplementary Materials.

### 4.1 Experimental section

#### 4.1.1 Experimental procedures and characterization data

##### 4.1.1.1 General procedure for reaction of aziridine with imine

Aziridine (0.20 mmol, 1.0 equiv.), imine (0.30 mmol, 1.5 equiv.), copper bromide (3.0 mg, 0.020 mmol, 10 mol%), and 2,9-dimethyl-1,10-phenanthroline (4.2 mg, 0.020 mmol, 10 mol%) were added with a stirring bar to a dried Schlenk tube. The tube was filled with nitrogen. Toluene (0.5 mL) was added to the tube and the mixture was stirred at 120°C for 20 h. The crude mixture was passed through a short pad of silica gel and concentrated *in vacuo*. Purification by chromatography on silica gel provided the desired product.

##### 4.1.1.2 General procedure for reaction of aziridine with isocyanate

Aziridine (0.20 mmol, 1.0 equiv.), isocyanate (0.30 mmol, 1.5 equiv.), copper bromide (3.0 mg, 0.020 mmol, 10 mol%), and 2,9-dimethyl-1,10-phenanthroline (4.2 mg, 0.020 mmol, 10 mol%) were added with a stirring bar to a dried Schlenk tube. The tube was filled with nitrogen. Toluene (0.5 mL) was added to the tube and the mixture was stirred at 120°C for 20 h. The crude mixture was passed through a short pad of silica gel and concentrated *in vacuo*. Purification by chromatography on silica gel provided the desired product.

## Data Availability

The original contributions presented in the study are included in the article/Supplementary Material, further inquiries can be directed to the corresponding author.
