# Supplementary material for: Copper-catalyzed reaction of aziridine for the synthesis of substituted imidazolidine and imidazolidinone
Source: Front Chem. 2023 Sep 29;11:1272034. doi: 10.3389/fchem.2023.1272034 (PMC10570437; doi:10.3389/fchem.2023.1272034)
Supplement: Supplementary file 1 [file DataSheet1.PDF]

Supplementary Material

Copper-Catalyzed Reaction of Aziridine for the Synthesis of Substituted Imidazolidine and imidazolidinone

Kota Hashimoto, Daiki Higuchi, Satoshi Matsubara, Kei Murakami*

*** Correspondence:** Kei Murakami: kei.murakami@kwansei.ac.jp

1 Experimental Procedures and Characterization Data

1. General Experimental Details

Unless otherwise noted, all reactants or reagents including dry solvents were obtained from commercial suppliers and used as received. Unless otherwise noted, all reactions were performed with dry solvents under an atmosphere of N_2 gas in dried glassware using standard vacuum-line techniques. Anhydrous toluene and CuBr were purchased from FUJIFILM Wako Pure Chemical Corporation. 2,9-dimethyl-1,10-phenanthroline was purchased from TCI. Isocyanate **4a** was purchased from Kanto Chemical Co., Inc., **4b** was purchased from Sigma-Aldrich, **4c**, **4d**, **4e**, **4f** were purchased from TCI. All work-up and purification procedures were carried out with reagent-grade solvents. Analytical thin-layer chromatography (TLC) was performed using Chem Scene HPTLC Silica Gel 60 GF254. Flash column chromatography was performed with Kanto Silica Gel 60 N (spherical, neutral) (40–50 µm). Silica-gel column chromatography was performed on an Isolera Spektra instrument equipped with a Biotage Sfär Silica HC D 10 g cartridge for 0.20 mmol scale reactions. Preparative recycling gel permeation chromatography (GPC) was performed with a JAI LC-9260 II NEXT instrument equipped with JAIGEL-2HR columns using chloroform as an eluent. High-resolution mass spectra were recorded on the JEOL JMS-T100LC spectrometer for electrospray ionization (ESI). Nuclear magnetic resonance (NMR) spectra were recorded on a JNM-ECX-500 (^{1}H 500 MHz, ^{13}C 126 MHz, ^{19}F 471 MHz) spectrometer. Chemical shifts for ^{1}H NMR are expressed in parts per million (ppm) relative to tetramethylsilane (δ 0.00 ppm). Chemical shifts for ^{13}C NMR are expressed in ppm relative to $CDCl_3$ (δ 77.16 ppm) and $C_2D_2Cl_4$ (δ 73.78 ppm). Chemical shifts for ^{19}F NMR are expressed in ppm relative to C_6F_6 (δ –163.00 ppm). Data are reported as follows: chemical shift, multiplicity (s = singlet, d = doublet, dd = doublet of doublets, t = triplet, q = quartet, m = multiplet), coupling constant (Hz), and integration.

1-1. Procedure for the synthesis of aziridine

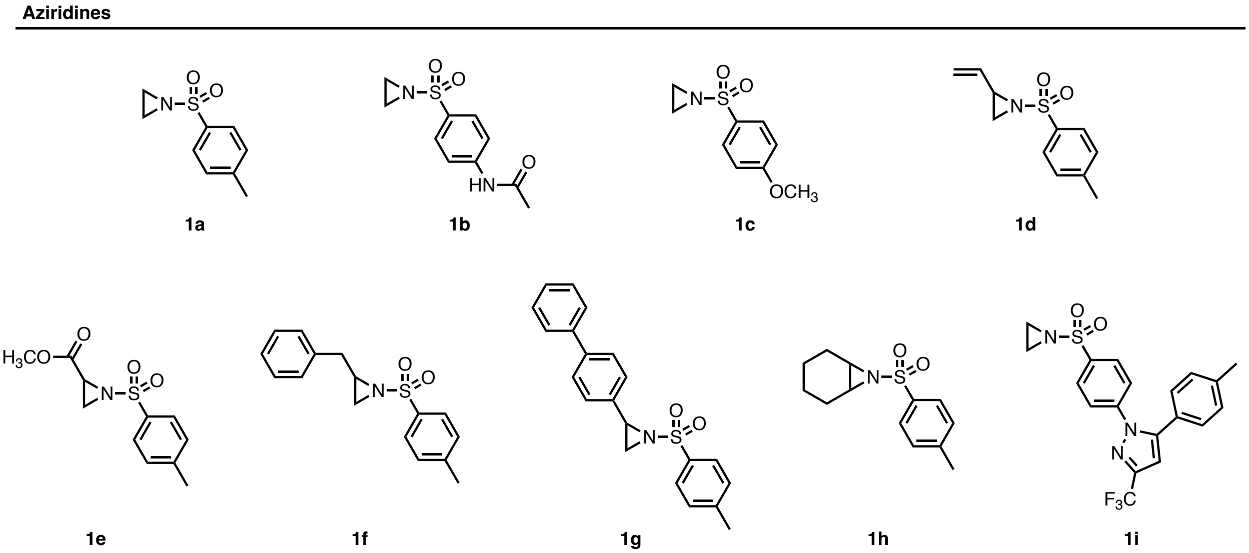

Aziridines as described above were prepared in our previous report (Higuchi, D., Matsubara, S., Kadowaki, H., Tanaka, D., and Murakami, K. (2023). Copper-Catalyzed Heterocyclic Recombination of Aziridine and Diazetidine for the Synthesis of Imidazolidine. Chem. Eur. J. 29, e202301071.).

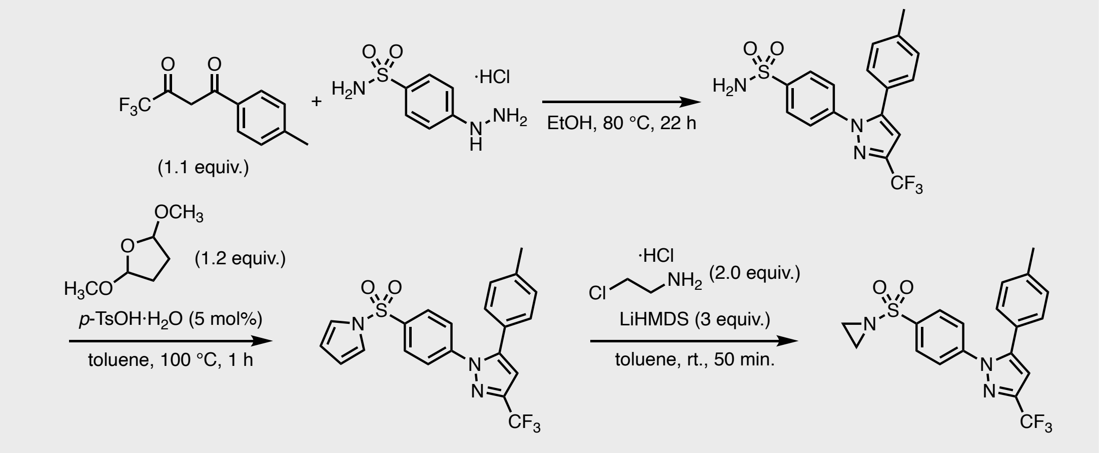

Celecoxib: Celecoxib was prepared according to the reported method (Chantal, S., and Darren, L. R. (2021). Improved batch and flow syntheses of the nonsteroidal anti-inflammatory COX-2 inhibitor celecoxib. React. Chem. Eng. 6, 138.). 4,4,4-Trifluoro-1-(4-methyl-phenyl)-butane-1,3-dione (1.21 g, 5.3 mmol, 1.1 equiv.) was added to a solution of 4-sulfamidophenylhydrazine hydrochloride (0.941 g, 5.0 mmol) in absolute ethanol (71 mL) followed by reaction at 80 °C for 22 h under a nitrogen atmosphere. The mixture was concentrated *in vacuo* to obtain celecoxib. The crude product was used at the next step without purification.

Sulfonyl pyrrole derived from Celecoxib: Sulfonyl pyrrole was prepared according to the reported method (Ozaki, T., Yorimitsu, H., and Perry, G. J. P. (2021). Primary Sulfonamide Functionalization

via Sulfonyl Pyrroles: Seeing the N-Ts Bond in a Different Light. Chem. Eur. J. 27, 15387–15391.). A 100-mL flask was charged with crude celecoxib, and placed under nitrogen atmosphere. Then, toluene (6.0 mL) and 2,5-dimethoxytetrahydrofuran (0.21 mL, 1.7 mmol, 1.1 equiv.) were added. *p*-TsOH·H_2O (0.014 g, 0.073 mmol, 5 mol%) was added at room temperature and the mixture was stirred at 100 °C for 1 h. The reaction was quenched with saturated $NaHCO_3$ aq. The resulting biphasic solution was extracted with EtOAc three times. The combined organic layers were washed with brine, dried over Na_2SO_4, and concentrated *in vacuo*. The crude mixture was passed through a short pad of silica gel by chloroform eluent and concentrated *in vacuo* to provide sulfonyl pyrrole as a white solid. The crude product was used at the next step without purification.

1i: **1i** was prepared according to the reported method (Higuchi, D., Matsubara, S., Kadowaki, H., Tanaka, D., and Murakami, K. (2023). Copper-Catalyzed Heterocyclic Recombination of Aziridine and Diazetidine for the Synthesis of Imidazolidine. Chem. Eur. J. 29, e202301071.). A Schlenk tube was charged with sulfonyl pyrrole (0.498 g, 1.2 mmol), LiHMDS (1.0 M in THF, 3.4 mL, 3.4 mmol, 2.8 equiv.) and toluene (10.0 mL). Then, 2-chloroethylamine hydrochloride (0.277 g, 2.4 mmol, 2.0 equiv.) was added and the mixture was stirred for 50 min. at room temperature. The reaction was quenched with water and then the resulting biphasic solution was extracted with EtOAc three times. The combined organic layer was washed with brine, dried over Na_2SO_4, and concentrated *in vacuo*. The crude mixture was purified by column chromatography on silica gel (hexane/EtOAc = 85:15) and GPC afforded **1i** (0.0507 g, 0.124 mmol, 11%) as a brown oil. ^{1}H NMR ($CDCl_3$ 500 MHz) δ 2.39–2.41 (m, 7H), 6.75 (s, 1H), 7.12 (d, *J* = 8.0 Hz, 2H), 7.19 (d, *J* = 8.0 Hz, 2H), 7.52 (d, *J* = 9.0 Hz, 2H), 7.94 (d, *J* = 9.0 Hz, 2H); ^{13}C NMR ($CDCl_3$ 126 MHz) δ 21.5, 27.9, 106.6, 121.1 (q, *J* = 271 Hz), 125.5, 125.7, 128.8, 129.2, 129.9, 137.4, 140.0, 143.5, 144.3 (q, *J* = 38.4 Hz), 145.4; The product is known and the spectroscopic properties are consistent with the data available in the literature.

Imines

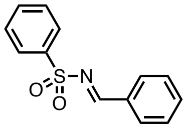

2a

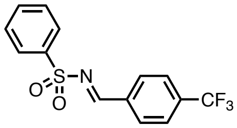

2b

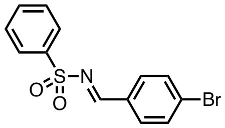

2c

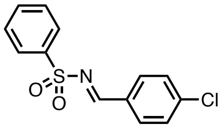

2d

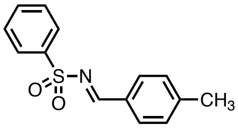

2e

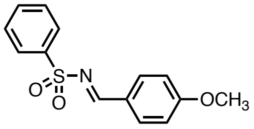

2f

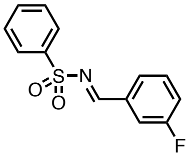

2g

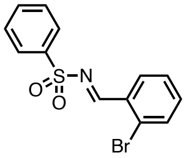

2h

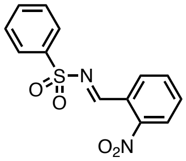

2i

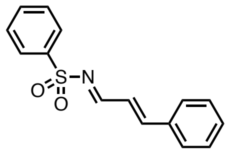

2j

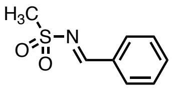

2k

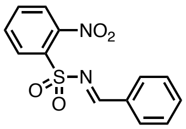

2l

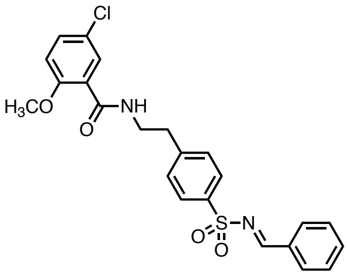

2m

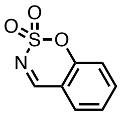

2n

Isocyanates

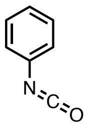

4a

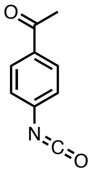

4b

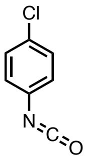

4c

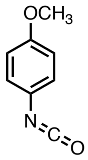

4d

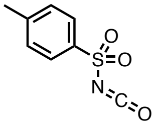

4e

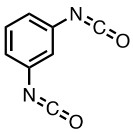

4f

1-2. Procedure for the synthesis of imine

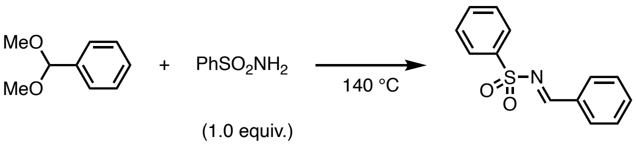

***N*-benzylidenebenzenesulfonamide (2a):** *N*-benzylidenebenzenesulfonamide was prepared according to the reported method (Bowman, R. K., and Johnson, J. S. (2004). Lewis Acid Catalyzed Dipolar Cycloadditions of an Activated Imidate. J. Org. Chem. 69, 8537–8540.). Benzaldehyde dimethylacetal (2.00 g, 13.1 mmol) and benzenesulfonamide (2.11 g, 13.4 mmol) were added to a 50 mL round–bottomed flask equipped with a stirring bar. This was heated to 140 °C and the reaction was stirred for 2 h. The crude mixture was concentrated *in vacuo* and purified by recrystallization (hexane/EtOAc) afforded **2a** (2.20 g, 8.96 mmol, 68%) as a white solid. ^{1}H NMR (CDCl$_3$ 500 MHz) δ 7.50 (t, J = 7.5 Hz, 2H), 7.56 (t, J = 7.5 Hz, 2H), 7.61–7.65 (m, 2H), 7.94 (d, J = 7.5 Hz, 2H), 8.02 (d, J = 7.5 Hz, 2H), 9.07 (s, 1H); ^{13}C NMR (CDCl$_3$ 126 MHz) δ 128.2, 129.31, 129.33, 131.5, 132.4, 133.7, 135.2, 138.3, 170.8; The product is known and the spectroscopic properties are consistent with the data available in the literature (Bowman, R. K., and Johnson, J. S. (2004). Lewis Acid Catalyzed Dipolar Cycloadditions of an Activated Imidate. J. Org. Chem. 69, 8537–8540.).

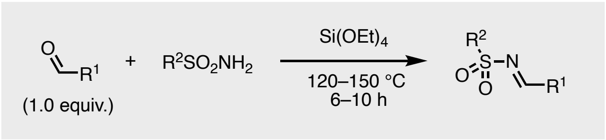

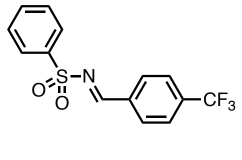

2b
51%

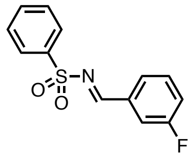

2g
42%

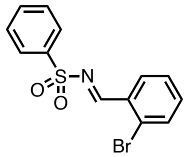

2h
45%

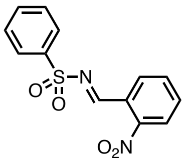

2i
26%

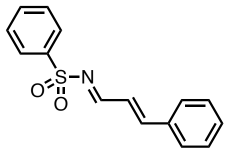

2j
58%

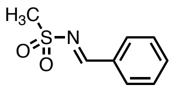

2k
35%

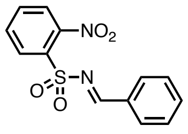

2l
26%

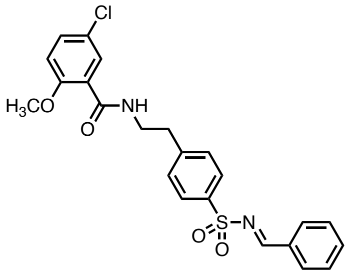

2m
40%

Method A: Imines as described above were prepared according to the reported method (Dong, D.-J., Li, H.-H., and Tian, S.-K. (2010). A Highly Tunable Stereoselective Olefination of Semistabilized Triphenylphosphonium Ylides with N-Sulfonyl Imines. J. Am. Chem. Soc. 132, 14, 5018–5020.). A test tube was charged with aldehyde (2.0 mmol), sulfonamide (2.0 mmol), and $Si(OEt)_4$ (0.54 mL, 1.2 equiv.) Then, the mixture was stirred at 120–150 °C for 6–10 h. The crude mixture was concentrated *in vacuo* and was purified by flash column chromatography or recrystallization.

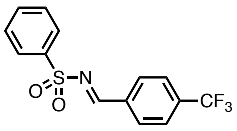

***N*-(4-(trifluoromethyl)benzylidene)benzenesulfonamide (2b):** The title compound was synthesized according to General Procedure (**Method A**) from benzenesulfonamide (0.315 g, 2.0 mmol scale).The reaction was stirred at 150 °C for 8 h. Purified by recrystallization (hexane/EtOAc) afforded **2b** (0.320 g, 1.02 mmol, 51%) as a white solid. ^{1}H NMR ($CDCl_3$ 500 MHz) δ 7.57–7.60 (m, 2H), 7.67 (t, *J* = 7.5 Hz, 1H), 7.76 (d, *J* = 8.5 Hz, 2H), 8.02–8.07 (m, 4H), 9.11 (s, 1H); ^{13}C NMR ($CDCl_3$ 126 MHz) δ 123.4 (q, *J* = 274 Hz), 126.2 (q, *J* = 3.7 Hz), 128.3, 129.4, 131.6, 134.1, 135.4, 136.0 (q, *J* = 32.5 Hz), 137.7, 169.0; The product is known and the spectroscopic properties are consistent with the data available in the literature (Truong, N., Sauer, S. J., Seraphin.-H, C., and Coltart, D. M. (2016). Direct carbon–carbon bond formation via reductive soft enolization: a syn-selective Mannich addition of α-iodo thioesters. Org. Biomol. Chem. 14, 7864.).

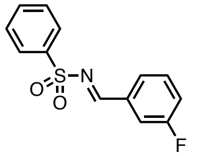

***N*-(3-fluorobenzylidene)benzenesulfonamide (2g):** The title compound was synthesized according to General Procedure (**Method A**) from benzenesulfonamide (0.316 g, 2.0 mmol scale). The reaction was stirred at 120 °C for 7 h. Purified by recrystallization (hexane/EtOAc) afforded **2g** (0.224 g, 0.852 mmol, 42%) as a white solid. ^{1}H NMR ($CDCl_3$ 500 MHz) δ 7.32 (td, *J* = 8.5, 3.0 Hz, 1H), 7.49 (td, *J* = 8.0, 5.5 Hz, 1H), 7.57 (t, *J* = 8.0 Hz, 2H), 7.64–7.70 (m, 3H), 8.01–8.03 (m, 2H), 9.04 (s, 1H); ^{13}C NMR ($CDCl_3$ 126 MHz) δ 116.7 (d, *J* = 22.8 Hz), 122.2 (d, *J* = 21.7 Hz), 128.1 (d, *J* = 3.7 Hz), 128.3, 129.4, 131.0 (d, *J* = 8.4 Hz), 133.9, 134.5 (d, *J* = 8.4 Hz), 137.9, 163.0 (d, *J* = 250 Hz), 169.3 (d, *J* = 2.4 Hz); The product is known and the spectroscopic properties are consistent with the data available in the literature (Omar, K., Gamal, G.-T., Maxime, D. C., Thierry, T., and Patrice, V. (2011). Original synthesis of 2-substituted-4,11-dimethoxy-1-(phenylsulfonyl)-2,3-dihydro-1*H*-naphtho[2,3-*f*]indole-5,10-diones using TDAE and Cu-catalyzed reaction strategy. Tetrahedron, 67, 6173–6180.).

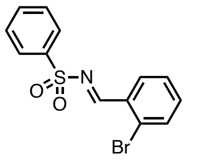

***N*-(2-bromobenzylidene)benzenesulfonamide (2h):** The title compound was synthesized according to General Procedure (**Method A**) from benzenesulfonamide (0.319 g, 2.0 mmol scale). The reaction

was stirred at 150 °C for 6 h. Purified by recrystallization (hexane/EtOAc) afforded **2h** (0.295 g, 0.911 mmol, 45%) as a brown solid. ^{1}H NMR (CDCl$_3$ 500 MHz) δ 7.39 (t, *J* = 7.5 Hz, 1H), 7.45 (td, *J* = 7.5, 2.0 Hz, 1H), 7.58 (t, *J* = 7.5 Hz, 2H), 7.66 (t, *J* = 7.5 Hz, 2H), 8.03 (d, *J* = 7.5 Hz, 2H), 8.16 (dd, *J* = 8.0, 2.0 Hz, 1H), 9.46 (s, 1H); ^{13}C NMR (CDCl$_3$ 126 MHz) δ 128.1, 128.4, 129.2, 129.4, 130.8, 131.3, 133.9, 134.0, 136.0, 137.8, 169.8; HRMS (ESI, positive): *m*/*z* = 345.9508 calcd for $C_{13}H_{10}BrNNaO_2S$: 345.9513 [M + Na]$^+$.

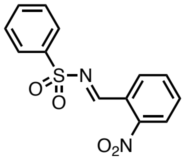

***N*-(2-nitrobenzylidene)benzenesulfonamide (2i):** The title compound was synthesized according to General Procedure (**Method A**) from benzenesulfonamide (0.317 g, 2.0 mmol scale). The reaction was stirred at 120 °C for 5 h. Purified by flash column chromatography on silica gel (hexane/EtOAc = 60:40) and GPC afforded **2i** (0.154 g, 0.529 mmol, 26%) as a white solid. ^{1}H NMR (CDCl$_3$ 500 MHz) δ 7.60 (t, *J* = 8.0 Hz, 2H), 7.68 (t, *J* = 8.0 Hz, 1H), 7.75–7.79 (m, 2H), 8.05 (d, *J* = 8.0 Hz, 2H), 8.11–8.17 (m, 2H), 9.51 (s, 1H); ^{13}C NMR (CDCl$_3$ 126 MHz) δ 125.2, 128.0, 128.6, 129.5, 130.7, 134.23, 134.25, 137.0, 149.8, 167.4; One sp^2 carbon peak was not observed because of overlapping. HRMS (ESI, positive): *m*/*z* = 313.0245 calcd for $C_{13}H_{10}N_2NaO_4S$: 313.0259 [M + Na]$^+$.

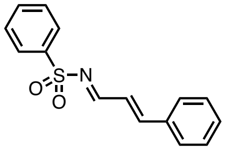

***N*-((1E,2E)-3-phenylallylidene)benzenesulfonamide (2j):** The title compound was synthesized according to General Procedure (**Method A**) from benzenesulfonamide (0.319 g, 2.0 mmol scale). The reaction was stirred at 120 °C for 10 h. Purified by flash column chromatography on silica gel (hexane/EtOAc = 60:40) and recrystallization (hexane/EtOAc) afforded **2j** (0.321 g, 1.18 mmol, 58%) as a yellow solid. ^{1}H NMR (CDCl$_3$ 500 MHz) δ 7.00 (dd, *J* = 16.0, 9.5 Hz, 1H), 7.41–7.65 (m, 9H), 7.98–8.00 (m, 2H), 8.81 (d, *J* = 9.0 Hz, 1H); ^{13}C NMR (CDCl$_3$ 126 MHz) δ 124.8, 128.0, 128.8, 129.29, 129.33, 131.9, 133.6, 134.2, 138.5, 154.3, 171.5; HRMS (ESI, positive): *m*/*z* = 294.0553 calcd for $C_{15}H_{13}NNaO_2S$: 294.0565 [M + Na]$^+$.

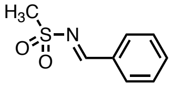

***N*-benzylidenemethanesulfonamide (2k):** The title compound was synthesized according to General Procedure (**Method A**) from methanesulfonamide (0.193 g, 2.0 mmol scale). The reaction was stirred at 120 °C for 7 h. Purified by recrystallization (hexane/EtOAc) afforded **2k** (0.132 g, 0.720 mmol, 35%) as a white solid. ^{1}H NMR (CDCl$_3$ 500 MHz) δ 3.15 (s, 3H), 7.55 (t, *J* = 7.5 Hz, 2H), 7.67 (d, *J* =7.5 Hz, 1H), 7.96–7.98 (m, 2H), 9.04 (s, 1H); ^{13}C NMR (CDCl$_3$ 126 MHz) δ 40.4, 129.4, 131.4, 132.2, 135.3, 171.8; The product is known and the spectroscopic properties are consistent with the data available in the literature (Ayonon, A., Nalbandian, C., Guillemard, L., and Gustafson, J. (2017). Benzylic bromination catalyzed by triphenylphosphine selenide via Lewis basic activation. Tetrahedron Lett. 58, 2940–2943.).

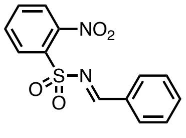

***N*-benzylidene-2-nitrobenzenesulfonamide (2l):** The title compound was synthesized according to General Procedure (**Method A**) from benzaldehyde (0.212 g, 2.0 mmol scale). The reaction was stirred at 120 °C for 6 h. Purified by flash column chromatography on silica gel (hexane/EtOAc = 55:45) afforded **2l** (0.150 g, 0.516 mmol, 26%) as a white solid. ^{1}H NMR ($CDCl_3$ 500 MHz) δ 7.52 (t, *J* = 8.0 Hz, 2H), 7.65–7.68 (m, 1H), 7.77–7.84 (m, 3H), 7.99 (dd, *J* = 8.5, 1.5 Hz, 2H), 8.36–8.40 (m, 1H), 9.08 (s, 1H); ^{13}C NMR ($CDCl_3$ 126 MHz) δ 124.9, 129.4, 131.7, 131.9, 132.1, 132.7, 134.8, 135.8, 148.5, 173.8; One sp^2 carbon peak was not observed because of overlapping. HRMS (ESI, positive): *m*/*z* = 313.0273 calcd for $C_{13}H_{10}N_2NaO_4S$: 313.0259 [M + Na]$^+$.

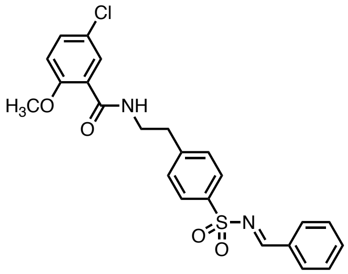

2m: The title compound was synthesized according to General Procedure (**Method A**) from benzaldehyde (0.098 g, 0.92 mmol scale). The reaction was stirred at 120 °C for 7 h. Purified by flash column chromatography (hexane/EtOAc = 50:50) and GPC afforded **2m** (0.170 g, 0.371 mmol, 40%) as a white solid. ^{1}H NMR ($CDCl_3$ 500 MHz) δ 3.02 (t, *J* = 7.0 Hz, 2H), 3.72–3.78 (m, 5H), 6.84 (d, *J* = 9.0 Hz, 1H), 7.34 (dd, *J* = 8.5, 3.0 Hz, 1H), 7.44 (d, *J* = 8.0 Hz, 2H), 7.49 (t, *J* = 7.5 Hz, 2H), 7.63 (t, *J* = 7.5 Hz, 1H), 7.82–7.84 (m, 1H), 7.92 (d, *J* = 7.5 Hz, 2H), 7.96 (d, *J* = 8.0 Hz, 2H), 8.13 (d, *J* = 3.0 Hz, 1H), 9.05 (s, 1H); ^{13}C NMR ($CDCl_3$ 126 MHz) δ 35.6, 40.6, 56.2, 112.9, 122.6, 126.6, 128.3, 129.3, 129.8, 131.4, 131.8, 132.2, 132.4, 135.2, 136.3, 145.9, 155.9, 164.0, 170.6; HRMS (ESI, positive): *m*/*z* = 479.0813 calcd for $C_{23}H_{21}ClN_2NaO_4S$: 479.0808 [M + Na]$^+$.

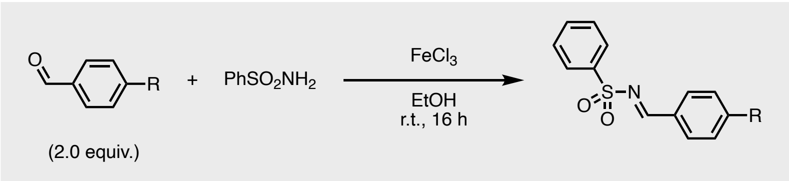

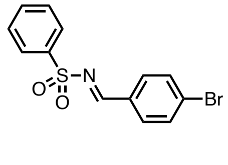

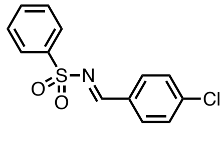

2c 44%

2d 38%

Method B: Imines as described above were prepared according to the reported method (Wu, X.-F, Bray, C. V.-L., Bechki, L., and Darcel, C. (2009). Iron-catalyzed sulfonylimine synthesis under

neutral conditions. Tetrahedron, 65, 7380–7384.). A test tube was charged with aldehyde (4.0 mmol), benzenesulfonamide (2.0 mmol), $FeCl_3$, and ethanol (8.0 mL). Then, the mixture was stirred at room temperature for 16 h. The crude mixture was concentrated *in vacuo* and was purified by recrystallization.

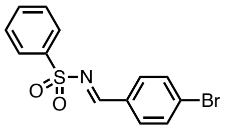

***N*-(4-bromobenzylidene)benzenesulfonamide (2c):** The title compound was synthesized according to General Procedure (**Method B**) from benzensulfonamide (0.324 g, 2.1 mmol scale). $FeCl_3$ (6.3 mg, 0.039 mmol, 2 mol%) was added. Purified by recrystallization (hexane/EtOAc) afforded **2c** (0.297 g, 0.917 mmol, 44%) as a white solid. ^{1}H NMR ($CDCl_3$ 500 MHz) δ 7.57 (t, J = 8.0 Hz, 2H), 7.64–7.67 (m, 3H), 7.80 (d, J = 8.0 Hz, 2H), 8.01 (d, J = 8.0 Hz, 2H), 9.01 (s, 1H); ^{13}C NMR ($CDCl_3$ 126 MHz) δ 128.2, 129.4, 130.6, 131.3, 132.6, 132.8, 133.9, 138.0, 169.4; The product is known and the spectroscopic properties are consistent with the data available in the literature (Chen, D., Chen, X., Du, Taiping., Kong, Li., Zhen, R., Zhen, S., Wen, Y. and Zhu, G. (2010). Highly efficient and diastereoselective synthesis of 1,3-oxazolidines featuring a palladium-catalyzed cyclization reaction of 2-butene-1,4-diol derivatives and imines. Tetrahedron Lett. 51, 5131–5133.).

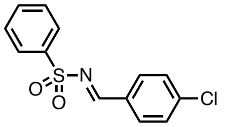

***N*-(4-bromobenzylidene)benzenesulfonamide (2d):** The title compound was synthesized according to General Procedure (**Method B**) from benzensulfonamide (0.310 g, 2.0 mmol scale). $FeCl_3$ (13.4 mg, 0.083 mmol, 4 mol%) was added. Purified by recrystallization (hexane/EtOAc) afforded **2d** (0.210 g, 0.751 mmol, 38%) as a white solid. ^{1}H NMR ($CDCl_3$ 500 MHz) δ 7.48 (d, J = 8.0 Hz, 2H), 7.57 (t, J = 8.0 Hz, 2H), 7.64–7.67 (m, 1H), 7.88 (d, J = 8.0 Hz, 2H), 8.00–8.02 (m, 2H), 9.03 (s, 1H); ^{13}C NMR ($CDCl_3$ 126 MHz) δ 128.2, 129.3, 129.8, 130.9, 132.6, 133.8, 138.1, 141.7, 169.3; The product is known and the spectroscopic properties are consistent with the data available in the literature (Chen, D., Chen, X., Du, Taiping., Kong, Li., Zhen, R., Zhen, S., Wen, Y. and Zhu, G. (2010). Highly efficient and diastereoselective synthesis of 1,3-oxazolidines featuring a palladium-catalyzed cyclization reaction of 2-butene-1,4-diol derivatives and imines. Tetrahedron Lett. 51, 5131–5133.).

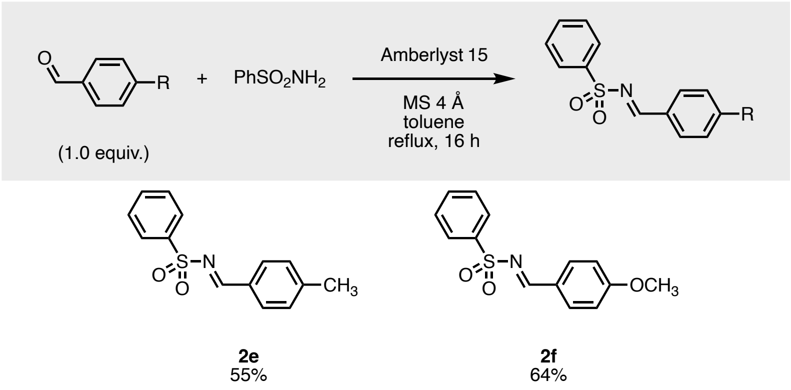

Method C: Imines as described above were prepared according to the reported method (Martzel, T., Lohier, J.-F., Gaumont, A.-C., Brière, J.-F., and Perrio, S. (2018). Sulfinate-Organocatalyzed (3+2) Annulation Reaction of Propargyl or Allenyl Sulfones with Activated Imines. Eur. J. Org. Chem. 36, 5069–5073.). A test tube was charged with aldehyde (2.0 mmol), benzenesulfonamide (2.0 mmol), Amberlyst 15, MS 4 Å, and toluene (3.5 mL). Then, the mixture was stirred at 120 °C for 16 h. The crude mixture was passed through a short pad of Celite and concentrated *in vacuo*. Purification by flash column chromatography on silica gel provided the desired product.

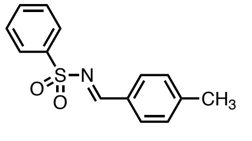

***N*-(4-methylbenzylidene)benzenesulfonamide (2e):** The title compound was synthesized according to General Procedure (**Method C**) from benzensulfonamide (0.317 g, 2.0 mmol scale). Amberlyst 15 (7.8 mg) and MS 4Å (12 mg) were added. Purified by flash column chromatography on silica gel (hexane/EtOAc = 75:25) afforded **2e** (0.288 g, 1.11 mmol, 55%) as a white solid. ^{1}H NMR ($CDCl_3$ 500 MHz) δ 2.43 (s, 3H), 7.30 (d, *J* = 8.5 Hz, 2H), 7.53–7.56 (m, 2H), 7.61–7.64 (m, 1H), 7.83 (d, *J* = 8.5 Hz, 2H), 8.00–8.02 (m, 2H), 9.02 (s, 1H); ^{13}C NMR ($CDCl_3$ 126 MHz) δ 22.2, 128.1, 129.3, 129.9, 130.1, 131.6, 133.6, 138.6, 146.7, 170.6; The product is known and the spectroscopic properties are consistent with the data available in the literature (Chen, D., Chen, X., Du, Taiping., Kong, Li., Zhen, R., Zhen, S., Wen, Y. and Zhu, G. (2010). Highly efficient and diastereoselective synthesis of 1,3-oxazolidines featuring a palladium-catalyzed cyclization reaction of 2-butene-1,4-diol derivatives and imines. Tetrahedron Lett. 51, 5131–5133.).

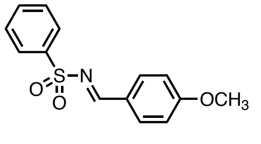

***N*-(4-methoxybenzylidene)benzenesulfonamide (2f):** The title compound was synthesized according to General Procedure (**Method C**) from benzensulfonamide (0.314 g, 2.0 mmol scale). Amberlyst 15 (8.1 mg) and MS 4Å (16 mg) were added. Purified by flash column chromatography (hexane/EtOAc = 60:40) afforded **2f** (0.349 g, 1.27 mmol, 64%) as a white solid. ^{1}H NMR ($CDCl_3$ 500 MHz) δ 3.88 (s, 3H), 6.97 (d, *J* = 8.0 Hz, 2H), 7.54 (t, *J* = 8.0 Hz, 2H), 7.60–7.63 (m, 1H), 7.90 (d, *J* = 8.0 Hz, 2H), 7.99–8.01 (m, 2H), 8.97 (s, 1H); ^{13}C NMR ($CDCl_3$ 126 MHz) δ 55.8, 114.8, 125.3, 128.0, 129.2, 133.4, 134.0, 138.9, 165.5, 169.8; The product is known and the spectroscopic properties are consistent with the data available in the literature (Chen, D., Chen, X., Du, Taiping., Kong, Li., Zhen, R., Zhen, S., Wen, Y. and Zhu, G. (2010). Highly efficient and diastereoselective synthesis of 1,3-oxazolidines featuring a palladium-catalyzed cyclization reaction of 2-butene-1,4-diol derivatives and imines. Tetrahedron Lett. 51, 5131–5133.).

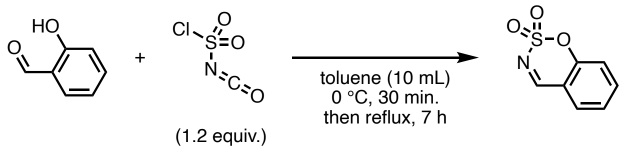

2n: Imine **2n** was synthesized according to the reported method (Luo, Z., Gao, Z.-H., Zhang, C.-L., Chen, K.-Q., and Ye, S. (2018). Enantioselective Synthesis of Cyclic α-Aminophosphonates through N-Heterocyclic Carbene-Catalyzed [4+2] Annulation of Enals with α-Iminophosphonates. Asian J. Org. Chem. 7, 2452–2455.) Chlorosulfonyl isocyanate (0.31 mL, 3.6 mmol) was slowly added to a solution of salicylaldehyde (0.368 g, 3.0 mmol) in toluene (10 mL) at 0 °C. After stirring the mixture at 0 °C for 30 min., the solution was refluxed for 7 h. Then, the reaction mixture was concentrated *in vacuo* and purified by flash column chromatography on silica gel (hexane/EtOAc = 65:35) afforded **2n** (0.272 g, 1.48 mmol, 49%) as a white solid. ^{1}H NMR ($CDCl_3$ 500 MHz) δ 7.27–7.32 (m, 1H), 7.43–7.47 (m, 1H), 7.71–7.74 (m, 1H), 7.75–7.80 (m, 1H), 8.69 (s, 1H); ^{13}C NMR ($CDCl_3$ 126 MHz) δ 115.4, 118.6, 126.4, 131.1, 137.8, 154.2, 168.1; The product is known and the spectroscopic properties are consistent with the data available in the literature (Spielmann, K., van der Lee, A., de Figueriedo, R. M., and Campagne, J.-M. (2018). Diastereoselective Palladium-Catalyzed (3+2)-Cycloadditions from Cyclic Imines and Vinyl Aziridines. Org. Lett. 20, 1444–1447.).

1-3. General procedure for reaction of aziridine with imine

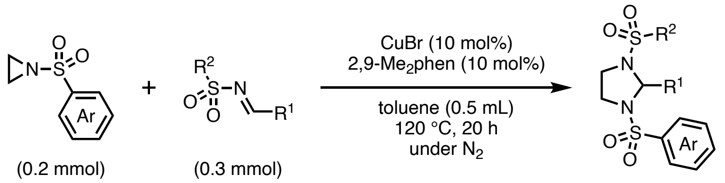

Method E: Aziridine (0.20 mmol, 1.0 equiv.), imine (0.30 mmol, 1.5 equiv.), copper bromide (3.0 mg, 0.020 mmol, 10 mol%), and 2,9-dimethyl-1,10-phenanthroline (4.2 mg, 0.020 mmol, 10 mol%) were added with a stirring bar to a dried Schlenk tube. The tube was filled with nitrogen. Toluene (0.5 mL) was added to the tube and the mixture was stirred at 120 °C for 20 h. The crude mixture was passed through a short pad of silica gel and concentrated *in vacuo*. Purification by chromatography on silica gel provided the desired product.

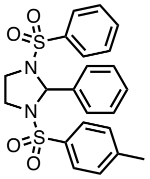

2-phenyl-1-(phenylsulfonyl)-3-tosylimidazolidine (3a): The title compound was synthesized according to General Procedure (**Method E**) (**1a** 39.1 mg, 0.20 mmol, **2a** 73.4 mg, 0.30 mmol scale). Purified by flash column chromatography on silica gel (hexane/EtOAc = 60:40) and GPC afforded **3a** (63.6 mg, 0.144 mmol, 72%) as a white solid. ^{1}H NMR ($CDCl_3$ 500 MHz) δ 2.43 (s, 3H), 3.41–3.48 (m, 4H), 6.41 (s, 1H), 7.25–7.31 (m, 5H), 7.34–7.35 (m, 2H), 7.46 (t, *J* = 8.5 Hz, 2H), 7.58–7.61 (m, 3H), 7.71 (dd, *J* = 8.5, 1.5 Hz, 2H); ^{13}C NMR ($CDCl_3$ 126 MHz) δ 21.7, 46.3, 46.4, 76.0, 126.9, 127.7, 127.8, 128.5, 128.8, 129.3, 130.0, 133.3, 134.8, 137.8, 138.7, 144.4; HRMS (ESI, positive): *m*/*z* = 465.0916 calcd for $C_{22}H_{22}N_2NaO_4S_2$: 465.0919 $[M + Na]^+$.

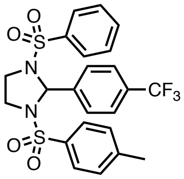

1-(phenylsulfonyl)-3-tosyl-2-(4-(trifluoromethyl)phenyl)imidazolidine (3b): The title compound was synthesized according to General Procedure (**Method E**) (**1a** 39.7 mg, 0.20 mmol, **2b** 93.6 mg, 0.30 mmol scale). Purified by flash column chromatography on silica gel (hexane/EtOAc = 60:40) and GPC afforded **3b** (81.9 mg, 0.160 mmol, 80%) as a colorless oil. ^{1}H NMR ($CDCl_3$ 500 MHz) δ 2.43 (s, 3H), 3.42–3.50 (m, 4H), 6.36 (s, 1H), 7.25–7.26 (m, 2H), 7.46–7.63 (m, 9H), 7.70–7.72 (m, 2H); ^{13}C NMR ($CDCl_3$ 126 MHz) δ 21.7, 46.4, 46.6, 75.2, 124.0 (q, *J* = 273 Hz), 125.5 (q, *J* = 3.7 Hz), 127.5, 127.69, 127.73, 129.4, 130.1, 131.0 (q, *J* = 32.4 Hz), 133.6, 134.3, 137.4, 142.7, 144.7; ^{19}F NMR ($CDCl_3$ 471 MHz) δ –63.9; HRMS (ESI, positive): *m*/*z* = 533.0784 calcd for $C_{23}H_{21}F_3N_2NaO_4S_2$: 533.0793 $[M + Na]^+$.

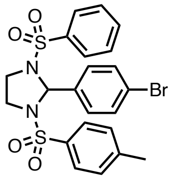

2-(2-bromophenyl)-1-(phenylsulfonyl)-3-tosylimidazolidine (3c): The title compound was synthesized according to General Procedure (**Method E**) (**1a** 39.4 mg, 0.20 mmol, **2c** 97.6 mg, 0.30 mmol scale). Purified by flash column chromatography on silica gel (hexane/EtOAc = 60:40) and GPC afforded **3c** (91.7 mg, 0.176 mmol, 88%) as a colorless oil. ^{1}H NMR ($CDCl_3$ 500 MHz) δ 2.43 (s, 3H), 3.40–3.46 (m, 4H), 6.29 (s, 1H), 7.22–7.27 (m, 4H), 7.40 (d, *J* = 9.0 Hz, 2H), 7.47 (t, *J* = 8.0 Hz, 2H), 7.57–7.62 (m, 3H), 7.69–7.70 (m, 2H); ^{13}C NMR ($CDCl_3$ 126 MHz) δ 21.7, 46.3, 46.4, 75.3, 123.0, 127.68, 127.72, 128.7, 129.4, 130.0, 131.6, 133.5, 134.5, 137.5, 137.9, 144.6; HRMS (ESI, positive): *m*/*z* = 543.0027 calcd for $C_{22}H_{21}BrN_2NaO_4S_2$: 543.0024 $[M + Na]^+$.

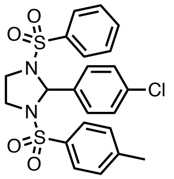

2-(4-chlorophenyl)-1-(phenylsulfonyl)-3-tosylimidazolidine (3d): The title compound was synthesized according to General Procedure (**Method E**) (**1a** 39.2 mg, 0.20 mmol, **2d** 84.4 mg, 0.30 mmol scale). Purified by flash column chromatography on silica gel (hexane/EtOAc = 50:50) and GPC afforded **3d** (79.7 mg, 0.167 mmol, 84%) as a white solid. ^{1}H NMR ($CDCl_3$ 500 MHz) δ 2.42 (s, 3H), 3.40–3.46 (m, 4H), 6.31 (s, 1H), 7.23–7.28 (m, 4H), 7.30 (d, *J* = 8.5 Hz, 2H), 7.47 (t, *J* = 8.5 Hz, 2H), 7.57–7.62 (m, 3H), 7.70 (d, *J* = 8.5 Hz, 2H); ^{13}C NMR ($CDCl_3$ 126 MHz) δ 21.7, 46.3, 46.4, 75.3, 127.67, 127.72, 128.4, 128.7, 129.4, 130.0, 133.5, 134.5, 134.7, 137.4, 137.5, 144.6; HRMS (ESI, positive): *m*/*z* = 499.0523 calcd for $C_{22}H_{21}ClN_2NaO_4S_2$: 499.0529 $[M + Na]^+$.

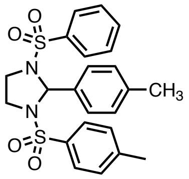

2-(4-methyl)-1-(phenylsulfonyl)-3-tosylimidazolidine (3e): The title compound was synthesized according to General Procedure (**Method E**) (**1a** 38.9 mg, 0.20 mmol, **2e** 78.5 mg, 0.30 mmol scale). Purified by flash column chromatography on silica gel (hexane/EtOAc = 60:40) and GPC afforded **3e** (27.2 mg, 0.060 mmol, 30%) as a white solid. ^{1}H NMR ($CDCl_3$ 500 MHz) δ 2.33 (s, 3H), 2.43 (s, 3H), 3.39–3.47 (m, 4H), 6.36 (s, 1H), 7.08 (d, *J* = 8.0 Hz, 2H), 7.18–7.26 (m, 4H), 7.46 (t, *J* = 8.0 Hz, 2H), 7.58–7.63 (m, 3H), 7.70–7.72 (m, 2H); ^{13}C NMR ($CDCl_3$ 126 MHz) δ 21.3, 21.8, 46.2, 46.3, 75.9, 126.9, 127.7, 127.8, 129.2, 129.3, 130.0, 133.3, 134.8, 135.8, 137.9, 138.7, 144.3; HRMS (ESI, positive): *m*/*z* = 479.1067 calcd for $C_{23}H_{24}N_2NaO_4S_2$: 479.1075 $[M + Na]^+$.

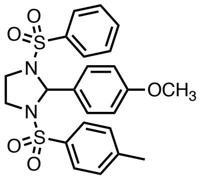

2-(4-methoxyphenyl)-1-(phenylsulfonyl)-3-tosylimidazolidine (3f): The title compound was synthesized according to General Procedure (**Method E**) (**1a** 40.2 mg, 0.20 mmol, **2f** 83.2 mg, 0.30 mmol scale). The reaction was stirred for 40 h. Purified by flash column chromatography on silica gel (hexane/EtOAc = 60:40) and GPC afforded **3f** (31.5 mg, 0.067 mmol, 33%) as a white solid. ^{1}H NMR ($CDCl_3$ 500 MHz) δ 2.43 (s, 3H), 3.42–3.48 (m, 4H), 3.80 (s, 3H), 6.33 (s, 1H), 6.79 (d, J = 9.0 Hz, 2H), 7.24–7.26 (m, 4H), 7.46 (t, J = 8.0 Hz, 2H), 7.58–7.61 (m, 3H), 7.69–7.71 (m, 2H); ^{13}C NMR ($C_2D_2Cl_4$ 126 MHz, 100 °C) δ 21.2, 46.06, 46.10, 55.3, 75.7, 113.9, 127.3, 127.4, 128.1, 128.9, 129.6, 130.8, 132.8, 138.3, 143.9, 159.9; One sp^2 carbon peak was not observed because of overlapping. HRMS (ESI, positive): m/z = 495.1030 calcd for $C_{23}H_{24}N_2NaO_5S_2$: 495.1024 [M + Na]$^+$.

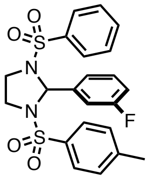

1-(phenylsulfonyl)-2-(3-fluorophenyl)-3-tosylimidazolidine (3g): The title compound was synthesized according to General Procedure (**Method E**) (**1a** 39.5 mg, 0.20 mmol, **2g** 79.4 mg, 0.30 mmol scale). Purified by flash column chromatography on silica gel (hexane/EtOAc = 60:40) and GPC afforded **3g** (74.4 mg, 0.162 mmol, 81%) as a white solid. ^{1}H NMR ($CDCl_3$ 500 MHz) δ 2.44 (s, 3H), 3.38–3.48 (m, 4H), 6.37 (s, 1H), 6.97–7.03 (m, 2H), 7.20 (d, J = 8.0 Hz, 1H), 7.25–7.29 (m, 3H), 7.48 (t, J = 8.0 Hz, 2H), 7.60–7.63 (m, 3H), 7.71–7.73 (m, 2H); ^{13}C NMR ($CDCl_3$ 126 MHz) δ 21.7, 46.3, 46.4, 75.2, 114.0 (d, J = 22.8 Hz), 115.8 (d, J = 20.4 Hz), 122.8 (d, J = 2.4 Hz), 127.7, 127.8, 129.4, 130.1, 130.2 (d, J = 7.2 Hz), 133.5, 134.5, 137.5, 141.4 (d, J = 7.2 Hz), 144.6, 162.7 (d, J = 248 Hz); ^{19}F NMR ($CDCl_3$ 471 MHz) δ –113.8; HRMS (ESI, positive): m/z = 483.0820 calcd for $C_{22}H_{21}FN_2NaO_4S_2$: 483.0825 [M + Na]$^+$.

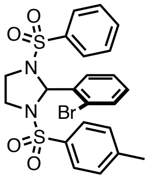

2-(2-bromophenyl)-1-(phenylsulfonyl)-3-tosylimidazolidine (3h): The title compound was synthesized according to General Procedure (**Method E**) (**1a** 40.6 mg, 0.20 mmol, **2h** 98.1 mg, 0.30 mmol scale). Purified by flash column chromatography on silica gel (hexane/EtOAc = 60:40) and GPC afforded **3h** (54.8 mg, 0.105 mmol, 51%) as a white solid. ^{1}H NMR ($CDCl_3$ 500 MHz) δ 2.41 (s, 3H), 3.44–3.50 (m, 2H), 3.65–3.71 (m, 2H), 6.43 (s, 1H), 7.12–7.22 (m, 4H), 7.31 (dd, J = 8.0, 1.5 Hz, 1H), 7.41 (t, J = 8.0 Hz, 2H), 7.51–7.56 (m, 2H), 7.61 (d, J = 9.0 Hz, 2H), 7.70–7.72 (m, 2H); ^{13}C NMR ($CDCl_3$ 126 MHz) δ 21.7, 47.0, 75.1, 123.4, 127.7, 127.8, 127.9, 129.2, 129.8, 129.9, 130.3, 133.2, 133.5, 134.0, 137.2, 138.1, 144.3; One sp^3 carbon peak was not observed because of

overlapping. HRMS (ESI, positive): *m*/*z* = 543.0034 calcd for $C_{22}H_{21}BrN_2NaO_4S_2$: 543.0024 [M + Na]$^+$.

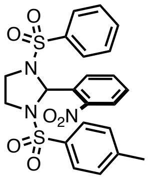

2-(2-nitrophenyl)-1-(phenylsulfonyl)-3-tosylimidazolidine (3i): The title compound was synthesized according to General Procedure (**Method E**) (**1a** 39.7 mg, 0.20 mmol, **2i** 87.7 mg, 0.30 mmol scale). Purified by flash column chromatography on silica gel (hexane/EtOAc = 45:55) afforded **3i** (80.5 mg, 0.165 mmol, 82%) as a white solid. ^{1}H NMR ($CDCl_3$ 500 MHz) δ 2.40 (s, 3H), 3.19–3.29 (m, 2H), 3.62–3.69 (m, 2H), 6.88 (s, 1H), 7.18 (d, *J* = 8.0 Hz, 2H), 7.41 (t, *J* = 8.0 Hz, 2H), 7.46–7.49 (m, 1H), 7.54–7.62 (m, 4H), 7.70–7.72 (m, 2H), 7.79 (dd, *J* = 8.0, 1.0 Hz, 1H), 7.92–7.94 (dd, *J* = 8.0, 1.0 Hz, 1H); ^{13}C NMR ($CDCl_3$ 126 MHz) δ 21.7, 47.16, 47.19, 69.7, 125.1, 127.8, 127.9, 129.3, 129.5, 129.6, 130.0, 133.0, 133.3, 133.4, 135.3, 136.2, 144.5, 148.5; HRMS (ESI, positive): *m*/*z* = 510.0753 calcd for $C_{22}H_{21}N_3NaO_6S_2$: 510.0770 [M + Na]$^+$.

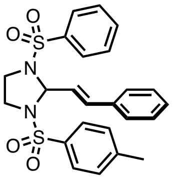

(*E*)-1-(phenylsulfonyl)-2-styryl-3-tosylimidazolidine (3j): The title compound was synthesized according to General Procedure (**Method E**) (**1a** 39.9 mg, 0.20 mmol, **2j** 81.9 mg, 0.30 mmol scale). Purified by flash column chromatography on silica gel (hexane/EtOAc = 60:40) and GPC afforded **3j** (32.7 mg, 0.070 mmol, 34%) as a yellow solid. ^{1}H NMR ($CDCl_3$ 500 MHz) δ 2.42 (s, 3H), 3.39–3.44 (m, 4H), 5.90–5.99 (m, 2H), 6.63 (d, *J* = 15.5 Hz, 1H), 7.24–7.32 (m, 7H), 7.47 (t, *J* = 9.0 Hz, 2H), 7.58–7.61 (m, 1H), 7.65 (d, *J* = 9.0 Hz, 2H), 7.76–7.78 (m, 2H); ^{13}C NMR ($CDCl_3$ 126 MHz) δ 21.7, 45.99, 46.05, 75.2, 125.0, 127.1, 127.75, 127.80, 128.5, 128.7, 129.4, 130.0, 133.3, 133.4, 134.9, 135.5, 138.0, 144.4; HRMS (ESI, positive): *m*/*z* = 491.1089 calcd for $C_{24}H_{24}N_2NaO_4S_2$: 491.1075 [M + Na]$^+$.

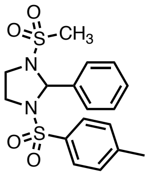

1-(methylsulfonyl)-2-phenyl-3-tosylimidazolidine (3k): The title compound was synthesized according to General Procedure (**Method E**) (**1a** 39.1 mg, 0.20 mmol, **2k** 55.0 mg, 0.30 mmol scale). Purified by flash column chromatography on silica gel (hexane/EtOAc = 50:50) afforded **3k** (63.6 mg, 0.167 mmol, 84%) as a white solid. ^{1}H NMR ($CDCl_3$ 500 MHz) δ 2.45 (s, 3H), 2.72 (s, 3H), 3.46–3.52 (m, 2H), 3.55–3.60 (m, 1H), 3.76–3.80 (m, 1H), 6.40 (s, 1H), 7.31–7.37 (m, 5H), 7.43–7.47 (m, 2H), 7.78 (d, *J* = 8.0 Hz, 2H); ^{13}C NMR ($CDCl_3$ 126 MHz) δ 21.8, 38.1, 46.4, 46.5, 75.6, 126.7, 128.0, 128.7, 128.9, 130.2, 134.5, 138.3, 144.9; The product is known and the spectroscopic

properties are consistent with the data available in the literature (Martinez, C., and Muñiz, K. (2015). An Iodine-Catalyzed Hofmann–Löffler Reaction. Angew. Chem. Int. Ed. 54, 8287–8291.).

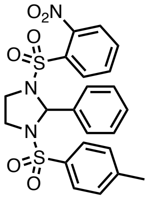

1-((2-nitrophenyl)sulfonyl)-2-phenyl-3-tosylimidazolidine (3l): The title compound was synthesized according to General Procedure (**Method E**) (**1a** 40.8 mg, 0.21 mmol, **2l** 87.4 mg, 0.30 mmol scale). Purified by flash column chromatography on silica gel (hexane/EtOAc = 60:40) and GPC afforded **3l** (68.0 mg, 0.139 mmol, 67%) as a white solid. ^{1}H NMR ($CDCl_3$ 500 MHz) δ 2.42 (s, 3H), 3.55–3.61 (m, 2H), 3.70–3.77 (m, 2H), 6.44 (s, 1H), 7.15–7.23 (m, 3H), 7.26–7.29 (m, 4H), 7.37–7.40 (m, 1H), 7.53–7.60 (m, 3H), 7.69 (d, J = 8.5 Hz, 2H); ^{13}C NMR ($CDCl_3$ 126 MHz) δ 21.7, 46.4, 47.2, 76.4, 124.0, 127.1, 127.9, 128.5, 129.0, 130.1, 131.0, 131.6, 132.3, 133.8, 134.3, 137.6, 144.7, 147.8; HRMS (ESI, positive): m/z = 510.0747 calcd for $C_{22}H_{21}N_3NaO_6S_2$: 510.0770 $[M + Na]^+$.

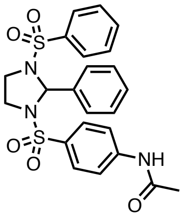

3m: The title compound was synthesized according to General Procedure (**Method E**) (**1b** 48.3 mg, 0.20 mmol, **2a** 73.9 mg, 0.30 mmol scale). Purified by flash column chromatography on silica gel (hexane/EtOAc = 25:75) and GPC afforded **3m** (24.2 mg, 0.050 mmol, 25%) as a white solid. ^{1}H NMR ($CDCl_3$ 500 MHz) δ 2.19 (s, 3H), 3.42–3.50 (m, 4H), 6.41 (s, 1H), 7.27–7.34 (m, 5H), 7.46 (t, J = 8.0 Hz, 2H), 7.58–7.70 (m, 8H); ^{13}C NMR ($CDCl_3$ 126 MHz) δ 24.9, 46.3, 46.4, 76.0, 119.4, 126.9, 127.7, 128.6, 128.9, 129.1, 129.4, 132.1, 133.5, 137.8, 138.5, 142.8, 168.9; HRMS (ESI, positive): m/z = 508.0964 calcd for $C_{23}H_{23}N_3NaO_5S_2$: 508.0977 $[M + Na]^+$.

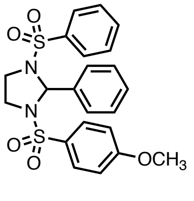

1-((4-methoxyphenyl)sulfonyl)-2-phenyl-3-(phenylsulfonyl)imidazolidine (3n): The title compound was synthesized according to General Procedure (**Method E**) (**1c** 42.8 mg, 0.20 mmol, **2a** 73.4 mg, 0.30 mmol scale). Purified by flash column chromatography on silica gel (hexane/EtOAc = 40:60) and GPC afforded **3n** (44.7 mg, 0.097 mmol, 49%) as a colorless oil. ^{1}H NMR ($CDCl_3$ 500 MHz) δ 3.39–3.58 (m, 4H), 3.87 (s, 3H), 6.40 (s, 1H), 6.92 (d, J = 9.0 Hz, 2H), 7.27–7.31 (m, 3H), 7.32–7.40 (m, 2H), 7.47 (t, J = 8.0 Hz, 2H), 7.59 (t, J = 8.0 Hz, 1H), 7.66 (d, J = 9.0 Hz, 2H), 7.69–7.77 (m, 2H); ^{13}C NMR ($CDCl_3$ 126 MHz) δ 46.3, 46.4, 55.8, 76.0, 114.6, 127.0, 127.7, 128.5, 128.8,

129.3, 130.0, 133.3, 137.9, 138.7, 163.6; One sp^2 carbon peak was not observed because of overlapping. HRMS (ESI, positive): *m*/*z* = 481.0857 calcd for $C_{22}H_{22}N_2NaO_5S_2$: 481.0868 $[M + Na]^+$.

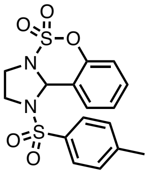

3o: The title compound was synthesized according to General Procedure (**Method E**) (**1a** 39.3 mg, 0.20 mmol, **2n** 54.8 mg, 0.30 mmol scale). Purified by flash column chromatography on silica gel (hexane/EtOAc = 70:30) afforded **3o** (75.2 mg, 0.198 mmol, 99%) as a yellow oil. ^1H NMR ($CDCl_3$ 500 MHz) δ 2.50 (s, 3H), 3.14–3.19 (m, 1H), 3.28–3.38 (m, 1H), 3.46–3.54 (m, 1H), 3.74–3.80 (m, 1H), 6.47 (s, 1H), 7.01 (d, *J* = 8.0 Hz, 1H), 7.29–7.34 (m, 1H), 7.39–7.42 (m, 3H), 7.74–7.77 (m, 1H), 7.82 (d, *J* = 8.0 Hz, 2H); ^{13}C NMR ($CDCl_3$ 126 MHz) δ 21.8, 45.9, 46.7, 76.8, 118.0, 118.3, 126.5, 128.0, 129.2, 130.5, 131.3, 133.5, 145.5, 149.9; HRMS (ESI, positive): *m*/*z* = 403.0386 calcd for $C_{16}H_{16}N_2NaO_5S_2$: 403.0398 $[M + Na]^+$.

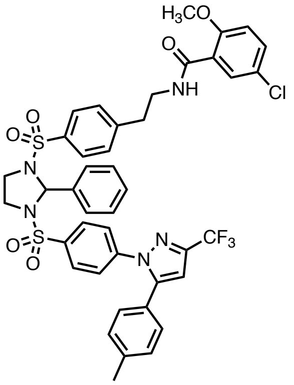

3p: The title compound was synthesized according to General Procedure (**Method E**) (**1i** 42.2 mg, 0.10 mmol, **2m** 68.6 mg, 0.15 mmol scale). The reaction was stirred for 40 h. Purified by flash column chromatography on silica gel (hexane/EtOAc = 50:50) and GPC afforded **3p** (45.7 mg, 0.053 mmol, 51%) as a white foam. ^1H NMR ($CDCl_3$ 500 MHz) δ 2.36 (s, 3H), 3.00 (t, *J* = 7.5 Hz, 2H), 3.39–3.46 (m, 3H), 3.52–3.58 (m, 1H), 3.69–3.75 (m, 2H), 3.78 (s, 3H), 6.42 (s, 1H), 6.74 (s, 1H), 6.85 (d, *J* = 8.5 Hz, 1H), 7.12 (d, *J* = 8.5 Hz, 2H), 7.17 (d, *J* = 8.5 Hz, 2H), 7.27–7.35 (m, 6H), 7.38 (d, *J* = 8.5 Hz, 2H), 7.43 (d, *J* = 8.5 Hz, 2H), 7.64 (d, *J* = 8.5 Hz, 2H), 7.68 (d, *J* = 8.5 Hz, 2H), 7.76–7.78 (m, 1H), 8.11 (d, *J* = 3.0 Hz, 1H); ^{13}C NMR ($CDCl_3$ 126 MHz) δ 21.5, 35.6, 40.8, 46.2, 46.4, 56.3, 76.0, 106.6, 112.9, 121.2 (q, *J* = 269 Hz), 123.0, 125.5, 125.7, 126.7, 126.8, 128.0, 128.6, 128.7, 128.9, 129.0, 129.9, 130.0, 131.9, 132.4, 135.8, 137.1, 138.3, 139.9, 143.1, 144.3 (q, *J* = 38.4 Hz), 145.4, 145.9, 156.0, 164.3; HRMS (ESI, positive): *m*/*z* = 886.1712 calcd for $C_{42}H_{37}ClF_3N_5NaO_6S_2$: 886.1724 $[M + Na]^+$.

1-4. General procedure for reaction of aziridine with isocyanate

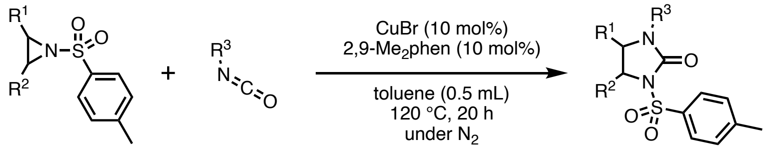

Method F: Aziridine (0.20 mmol, 1.0 equiv.), isocyanate (0.30 mmol, 1.5 equiv.), copper bromide (3.0 mg, 0.020 mmol, 10 mol%), and 2,9-dimethyl-1,10-phenanthroline (4.2 mg, 0.020 mmol, 10 mol%) were added with a stirring bar to a dried Schlenk tube. The tube was filled with nitrogen. Toluene (0.5 mL) was added to the tube and the mixture was stirred at 120 °C for 20 h. The crude mixture was passed through a short pad of silica gel and concentrated in vacuo. Purification by chromatography on silica gel provided the desired product.

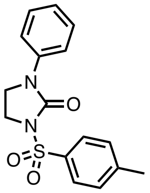

1-phenyl-3-tosylimidazolidin-2-one (5a): The title compound was synthesized according to General Procedure (**Method F**) (**1a** 40.1 mg, 0.20 mmol, **4a** 43.1 mg, 0.36 mmol scale). Purified by trituration (hexane/$CHCl_3$) afforded **5a** (47.9 mg, 0.151 mmol, 74%) as a white solid. ^{1}H NMR ($CDCl_3$ 500 MHz) δ 2.43 (s, 3H), 3.85–3.88 (m, 2H), 3.99–4.02 (m, 2H), 7.10 (t, J = 7.0 Hz, 1H), 7.30–7.35 (m, 4H), 7.44 (d, J = 8.5 Hz, 2H), 7.98 (d, J = 8.5 Hz, 2H); ^{13}C NMR ($CDCl_3$ 126 MHz) δ 21.8, 41.3, 42.5, 118.8, 124.5, 128.5, 129.1, 129.8, 134.9, 138.5, 145.2, 151.8; HRMS (ESI, positive): m/z = 339.0785 calcd for $C_{16}H_{16}N_2NaO_3S$: 339.0779 $[M + Na]^+$.

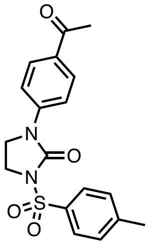

1-(4-acetylphenyl)-3-tosylimidazolidin-2-one (5b): The title compound was synthesized according to General Procedure (**Method F**) (**1a** 39.5 mg, 0.20 mmol, **4b** 48.5 mg, 0.30 mmol scale). Purified by trituration (hexane/CH_2Cl_2) afforded **5b** (42.9 mg, 0.120 mmol, 60%) as a yellow solid. ^{1}H NMR ($CDCl_3$ 500 MHz) δ 2.45 (s, 3H), 2.56 (s, 3H), 3.91–3.94 (m, 2H), 4.04–4.07 (m, 2H), 7.37 (d, J = 8.0 Hz, 2H), 7.57 (d, J = 9.0 Hz, 2H), 7.93 (d, J = 9.0 Hz, 2H), 7.99 (d, J = 8.0 Hz, 2H); ^{13}C NMR ($CDCl_3$ 126 MHz) δ 21.8, 26.6, 41.1, 42.2, 117.6, 128.4, 129.6, 129.9, 132.7, 134.7, 142.7, 145.5, 151.6, 197.0; HRMS (ESI, positive): m/z = 381.0879 calcd for $C_{18}H_{18}N_2NaO_4S$: 381.0885 $[M + Na]^+$.

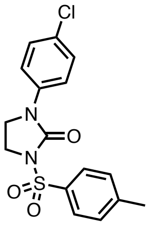

1-(4-chlorophenyl)-3-tosylimidazolidin-2-one (5c): The title compound was synthesized according to General Procedure (**Method F**) (**1a** 38.9 mg, 0.20 mmol, **4c** 48.1 mg, 0.31 mmol scale). Purified by recrystallization (hexane / EtOAc) afforded **5c** (57.2 mg, 0.163 mmol, 83%) as a white solid. ^{1}H NMR ($CDCl_3$ 500 MHz) δ 2.44 (s, 3H), 3.83–3.87 (m, 2H), 4.00–4.03 (m, 2H), 7.28 (d, J = 9.0 Hz, 2H), 7.35 (d, J = 8.0 Hz, 2H), 7.40 (d, J = 9.0 Hz, 2H), 7.98 (d, J = 8.0 Hz, 2H); ^{13}C NMR ($CDCl_3$ 126 MHz) δ 21.8, 41.2, 42.4, 119.8, 128.4, 129.1, 129.6, 129.9, 134.8, 137.1, 145.3, 151.7; HRMS (ESI, positive): m/z = 373.0389 calcd for $C_{16}H_{15}ClN_2NaO_3S$: 373.0390 $[M + Na]^+$.

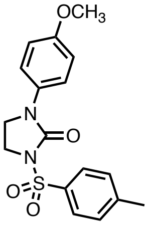

1-(4-methoxyphenyl)-3-tosylimidazolidin-2-one (5d): The title compound was synthesized according to General Procedure (**Method F**) (**1a** 40.0 mg, 0.20 mmol, **4d** 38.4 mg, 0.26 mmol scale). Purified by trituration (hexane/CH_2Cl_2) afforded **5d** (52.2 mg, 0.151 mmol, 74%) as a white solid. ^{1}H NMR ($CDCl_3$ 500 MHz) δ 2.43 (s, 3H), 3.77 (s, 3H), 3.81–3.84 (m, 2H), 3.97–4.00 (m, 2H), 6.85 (d, J = 9.5 Hz, 2H), 7.32–7.35 (m, 4H), 7.98 (d, J = 8.5 Hz, 2H); ^{13}C NMR ($CDCl_3$ 126 Hz) δ 21.8, 41.4, 43.0, 55.6, 114.3, 120.9, 128.4, 129.8, 131.6, 134.9, 145.1, 152.0, 156.7; HRMS (ESI, positive): m/z = 369.0877 calcd for $C_{17}H_{18}N_2NaO_4S$: 369.0885 $[M + Na]^+$.

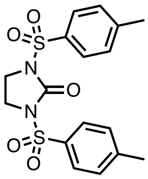

1,3-ditosylimidazolidin-2-one (5e): The title compound was synthesized according to General Procedure (**Method F**) (**1a** 39.7 mg, 0.20 mmol, **4e** 44.6 mg, 0.23 mmol scale). Purified by flash column chromatography on silica gel (hexane/EtOAc = 70:30) afforded **5e** (58.1 mg, 0.129 mmol, 64%) as a white solid. ^{1}H NMR ($CDCl_3$ 500 MHz) δ 2.46 (s, 6H), 3.84 (s, 4H), 7.36 (d, J = 8.0 Hz, 4H), 7.88 (d, J = 8.0 Hz, 4H); ^{13}C NMR ($CDCl_3$ 126 MHz) δ 21.9, 41.8, 128.3, 130.2, 133.9, 146.0, 149.2; HRMS (ESI, positive): m/z = 417.0564 calcd for $C_{17}H_{18}N_2NaO_5S_2$: 417.0555 $[M + Na]^+$.

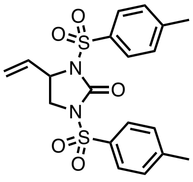

1,3-ditosyl-4-vinylimidazolidin-2-one (5f): The title compound was synthesized according to General Procedure (**Method F**) (**1d** 44.1 mg, 0.20 mmol, **4e** 54.3 mg, 0.28 mmol scale). Purified by column chromatography on silica gel (hexane/EtOAc = 70:30) and GPC afforded **5f** (35.9 mg, 0.085 mmol, 43%) as a white solid. ^{1}H NMR (CDCl$_3$ 500 MHz) δ 2.44 (s, 3H), 2.45 (s, 3H), 3.70 (dd, *J* = 9.0, 3.0 Hz, 1H), 3.90 (t, *J* = 9.0 Hz, 1H), 4.74 (td, *J* = 9.0, 3.0 Hz, 1H), 5.33 (d, *J* = 9.0 Hz, 1H), 5.42 (d, *J* = 16.5 Hz, 1H), 5.72–5.79 (m, 1H), 7.30 (d, *J* = 8.0 Hz, 2H), 7.35 (d, *J* = 8.0 Hz, 2H), 7.85–7.89 (m, 4H); ^{13}C NMR (CDCl$_3$ 126 MHz) δ 21.86, 21.88, 48.3, 56.7, 120.7, 128.3, 128.8, 129.8, 130.2, 133.6, 133.9, 135.2, 145.6, 145.9, 148.8; HRMS (ESI, positive): *m*/*z* = 443.0727 calcd for $C_{19}H_{20}N_2NaO_5S_2$: 443.0711 [M + Na]$^+$.

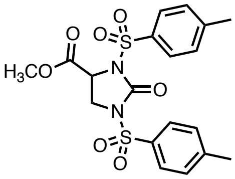

(5g): The title compound was synthesized according to General Procedure (**Method F**) (**1e** 51.2 mg, 0.20 mmol, **4e** 58.5 mg, 0.30 mmol scale). Purified by column chromatography on silica gel (hexane/EtOAc = 60:40) and GPC afforded **5g** (50.6 mg, 0.112 mmol, 56%) as a white solid. ^{1}H NMR (CDCl$_3$ 500 MHz) δ 2.44 (s, 6H), 3.76 (s, 3H), 3.97 (dd, *J* = 10.0, 4.0 Hz, 1H), 4.02 (t, *J* = 10.0 Hz, 1H), 4.84 (dd, *J* = 10.0, 4.0 Hz, 1H), 7.33–7.35 (m, 4H), 7.86 (d, *J* = 8.5 Hz, 2H), 7.93 (d, *J* = 8.5 Hz, 2H); ^{13}C NMR (CDCl$_3$ 126 MHz) δ 21.8, 21.9, 45.5, 53.5, 53.9, 128.3, 129.3, 129.6, 130.2, 133.8, 134.3, 145.9, 146.1, 148.4, 168.8; HRMS (ESI, positive): *m*/*z* = 475.0601 calcd for $C_{19}H_{20}N_2NaO_7S_2$: 475.0610 [M + Na]$^+$.

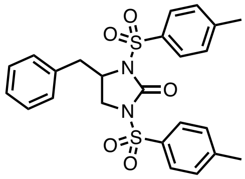

4-benzyl-1,3-ditosylimidazolidin-2-one (5h): The title compound was synthesized according to General Procedure (**Method F**) (**1f** 57.5 mg, 0.20 mmol, **4e** 64.0 mg, 0.32 mmol scale). Purified by column chromatography on silica gel (hexane/EtOAc = 55:45) and GPC afforded **5h** (50.3 mg, 0.104 mmol, 52%) as a white solid. ^{1}H NMR (CDCl$_3$ 500 MHz) δ 2.44 (s, 3H), 2.45 (s, 3H), 2.74 (dd, *J* = 13.0, 9.5 Hz, 1H), 3.35 (dd, *J* = 13.0, 3.5 Hz, 1H), 3.56 (t, *J* = 9.5 Hz, 1H), 3.72 (dd, *J* = 9.5, 2.5 Hz, 1H), 4.48–4.52 (m, 1H), 7.18 (d, *J* = 8.0 Hz, 2H), 7.27–7.34 (m, 7H), 7.77 (d, *J* = 8.0 Hz, 2H), 7.95 (d, *J* = 8.0 Hz, 2H); ^{13}C NMR (CDCl$_3$ 126 MHz) δ 21.8, 39.7, 46.0, 55.3, 127.6, 128.1, 128.5, 129.2, 129.6, 130.0, 130.1, 134.1, 134.8, 135.5, 145.7, 149.0; One sp^2 carbon peak and one sp^3 carbon peak were not observed because of overlapping. HRMS (ESI, positive): *m*/*z* = 507.1030 calcd for $C_{24}H_{24}N_2NaO_5S_2$: 507.1024 [M + Na]$^+$.

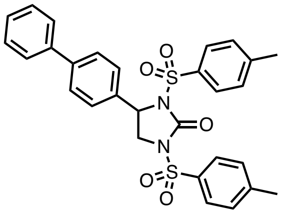

4-([1,1'-biphenyl]-4-yl)-1,3-ditosylimidazolidin-2-one (**5i**): The title compound was synthesized according to General Procedure (**Method F**) (**1g** 69.9 mg, 0.20 mmol, **4e** 59.2 mg, 0.30 mmol scale). Purified by recrystallization (hexane/CH_2Cl_2) afforded **5i** (39.7 mg, 0.073 mmol, 36%) as a yellow solid. ^{1}H NMR ($CDCl_3$ 500 MHz) δ 2.35 (s, 3H), 2.48 (s, 3H), 3.98 (dd, *J* = 9.0, 2.0 Hz, 1H), 4.14 (t, *J* = 9.0 Hz, 1H), 5.29 (dd, *J* = 9.0, 2.0 Hz, 1H), 7.06 (d, *J* = 8.5 Hz, 2H), 7.24 (d, *J* = 8.5 Hz, 2H), 7.36–7.42 (m, 5H), 7.47–7.50 (m, 4H), 7.55–7.57 (m, 2H), 7.93 (d, *J* = 8.5 Hz, 2H); ^{13}C NMR ($CDCl_3$ 126 MHz) δ 21.8, 21.9, 50.2, 56.9, 127.2, 127.4, 127.8, 128.0, 128.35, 128.43, 129.1, 129.4, 130.2, 134.0, 134.9, 136.9, 140.3, 142.4, 145.3, 146.0, 149.1; HRMS (ESI, positive): *m*/*z* = 569.1160 calcd for $C_{29}H_{26}N_2NaO_5S_2$: 569.1181 [M + Na]$^+$.

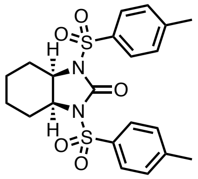

1,3-ditosyloctahydro-2*H*-benzo[*d*]imidazol-2-one (5j): The title compound was synthesized according to General Procedure (**Method F**) (**1h** 54.0 mg, 0.21 mmol, **4e** 52.4 mg, 0.26 mmol scale). Purified by flash column chromatography on silica gel (hexane/EtOAc = 75:25) and GPC afforded **5j** (21.5 mg, 0.048 mmol, 22%) as a white solid. ^{1}H NMR ($CDCl_3$ 500 MHz) δ 1.39–1.42 (m, 2H), 1.53–1.58 (m, 2H), 1.89–1.94 (m, 2H), 2.06–2.09 (m, 2H), 2.45 (s, 6H), 4.06–4.10 (m, 2H), 7.31 (d, *J* = 8.0 Hz, 4H), 7.87 (d, *J* = 8.0 Hz, 4H); ^{13}C NMR ($CDCl_3$ 126 MHz) δ 19.7, 21.9, 27.2, 56.4, 128.5, 129.9, 135.0, 145.5, 150.8; HRMS (ESI, positive): *m*/*z* = 471.1041 calcd for $C_{21}H_{24}N_2NaO_5S_2$: 471.1024 [M + Na]$^+$.

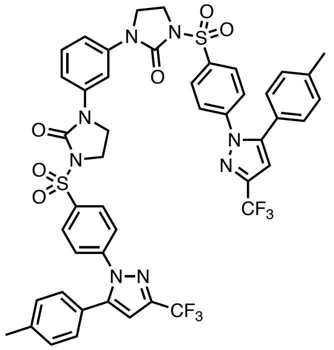

5k: The title compound was synthesized according to General Procedure (**Method F**) (**1i** 128.6 mg, 0.32 mmol, **4f** 16.5 mg, 0.10 mmol, copper bromide 3.5 mg, 0.024 mmol, 24 mol%, 2,9-dimethyl-1,10-phenanthroline, 4.7 mg, 0.023 mmol, 23 mol% scale). Purified by flash column chromatography on silica gel (hexane/EtOAc = 55:45) afforded **5k** (95.3 mg, 0.098 mmol, 95%) as a white solid. ^{1}H NMR ($CDCl_3$ 500 MHz) δ 2.37 (s, 6H), 3.77–4.04 (m, 8H), 6.73 (s, 2H), 7.11 (d, *J* = 8.0 Hz, 4H), 7.16–7.18 (m, 6H), 7.21–7.25 (m, 1H), 7.50 (d, *J* = 8.5 Hz, 4H), 7.61–7.71 (m, 1H), 8.05 (d, *J* = 8.5 Hz, 4H); ^{13}C NMR ($CDCl_3$ 126 MHz) δ 21.4, 41.2, 42.4, 106.6, 108.8, 114.4, 121.1 (q, *J* = 270 Hz), 125.4, 125.7, 128.8, 129.4, 129.5, 129.9, 136.9, 139.0, 140.0, 143.7, 144.3 (q, *J* = 38.4 Hz), 145.4, 151.5; HRMS (ESI, positive): *m*/*z* = 997.1976 calcd for $C_{46}H_{36}F_6N_8NaO_6S_2$: 997.2001 [M + Na]$^+$.

2 Possible reaction mechanism

An alternative reaction mechanism is shown in Supplementary Figure 1. The reaction initiates from the coordination of imine **2** to copper **A** to form intermediate **D**. Then, the reaction between **D** and aziridine **1** to form intermediate **E**, which finally affords product **3**.

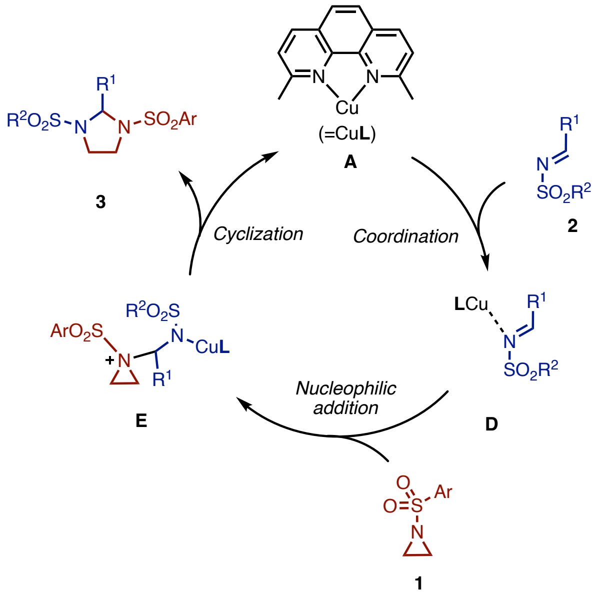

Supplementary Figure 1. Possible reaction mechanism.

3 Screening of catalyst

We investigated the effect of the catalysts. Other copper salts such as CuCl, CuOAc, CuCN, $CuBr_2$, or $CuCl_2$ were not effective for the reaction.

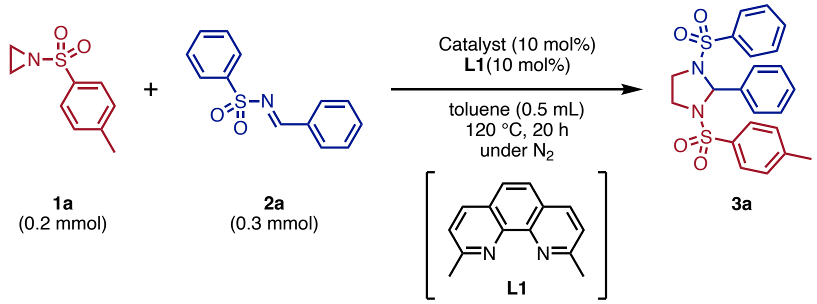

entry	cat.	NMR yield
1	CuCl	29%
2	CuOAc	n.d.
3	CuCN	1%
4	$CuBr_2$	28%
5	$CuCl_2$	6%

Supplementary Figure 2. Effect of other copper salts.

4 Competitive experiment

A competitive experiment revealed that the reaction of electron-deficient imine took place much faster than that of electron-rich one. As shown in Figure 5a, we performed the reaction of **1a** with a slight excess amount of the mixture of **2b** and **2f**. Interestingly, product **3b** was obtained in a quantitative yield, whereas **3f** was barely detected.

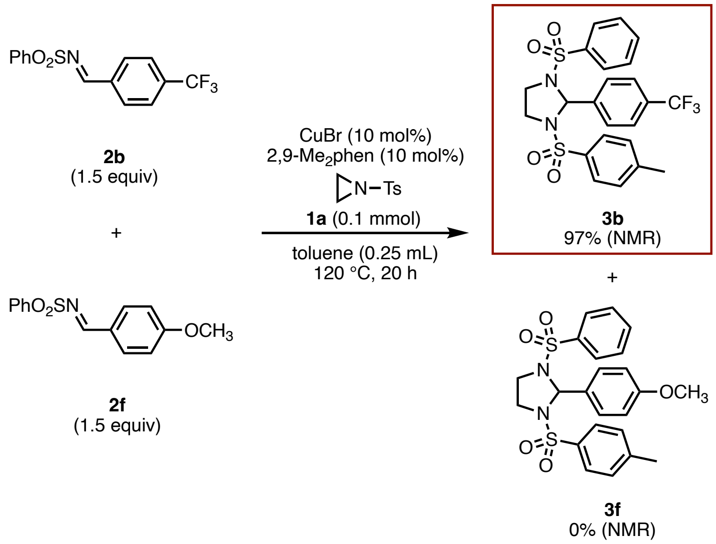

Supplementary Figure 3. Competitive experiments. ^{1}H NMR yields were shown using 1,1,2,2-tetrachloroethane.

5 NMR Spectra

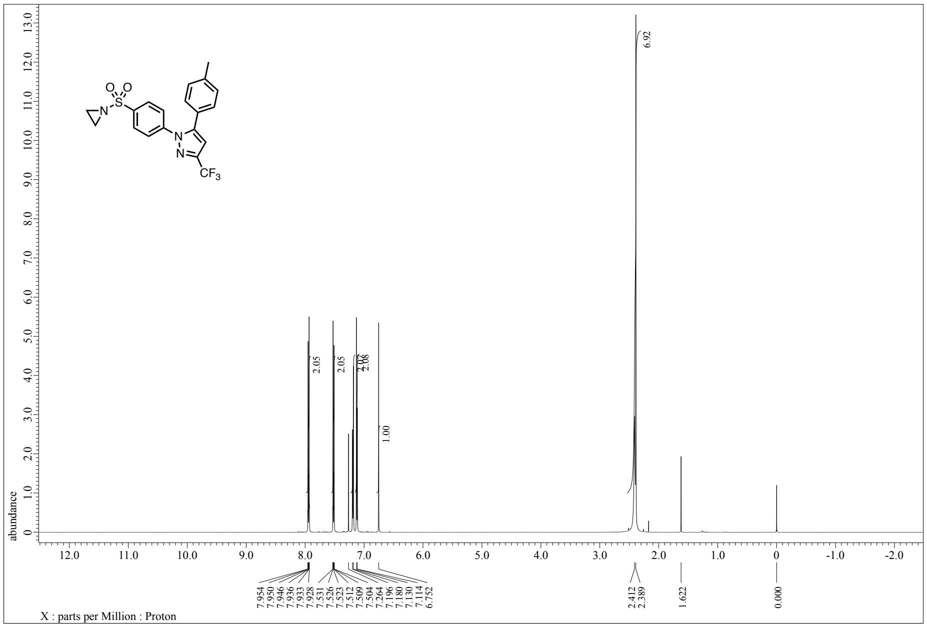

Supplementary Figure 4. ^{1}H NMR (500 MHz $CDCl_3$) spectrum of **1i**.

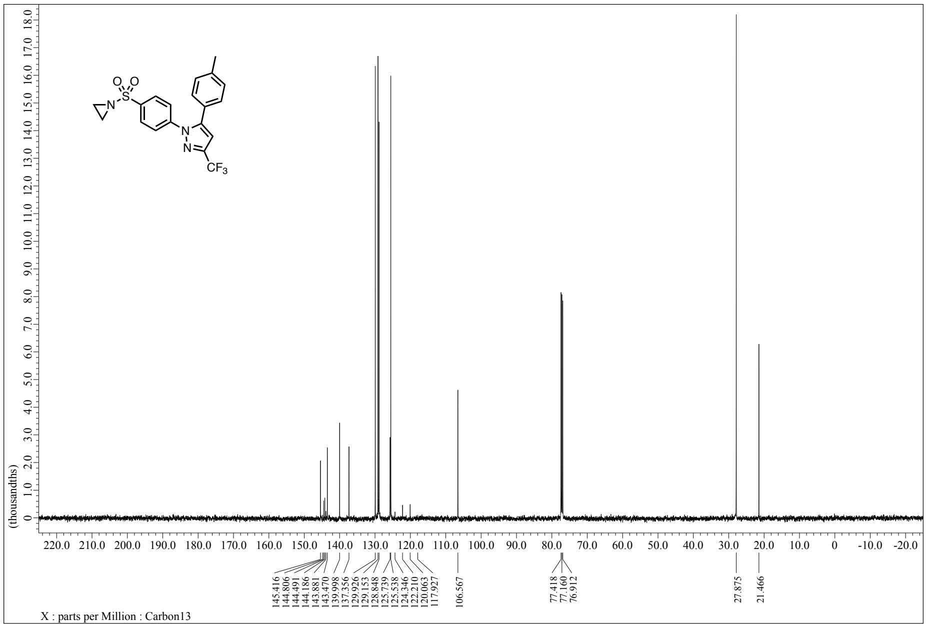

Supplementary Figure 5. ^{13}C NMR (126 MHz $CDCl_3$) spectrum of **1i**.

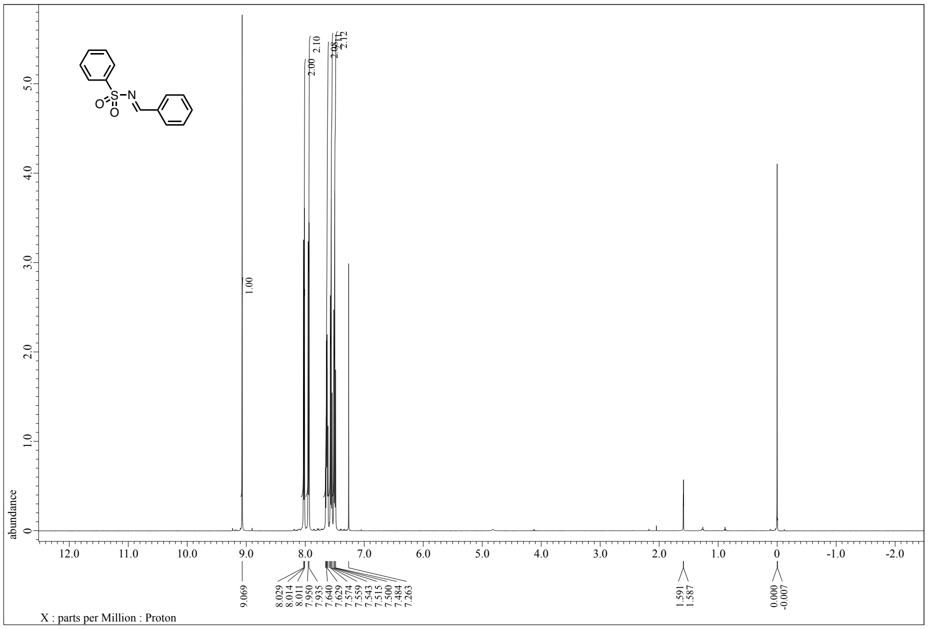

Supplementary Figure 6. ^{1}H NMR (500 MHz $CDCl_3$) spectrum of **2a**.

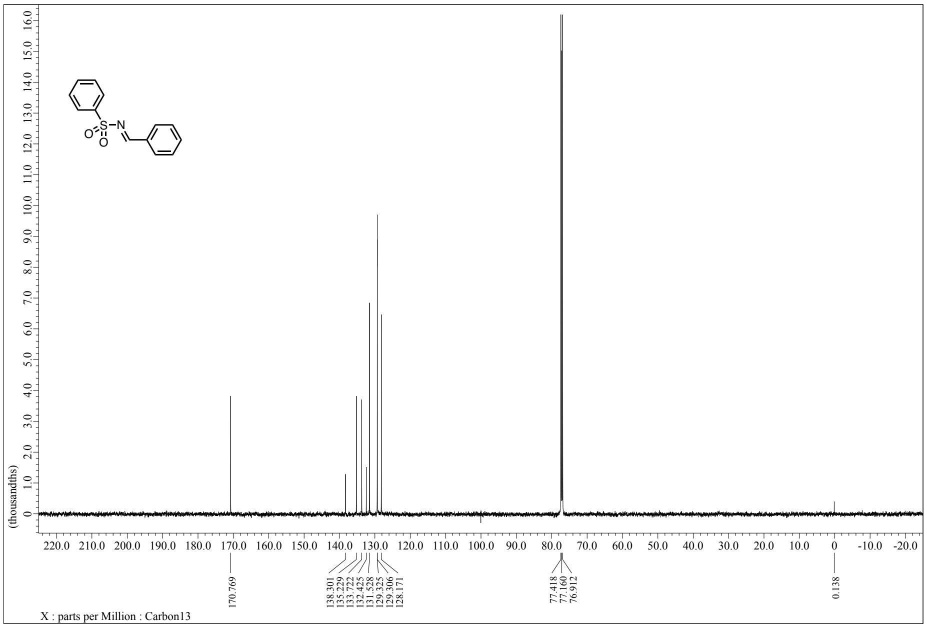

Supplementary Figure 7. ^{13}C NMR (126 MHz $CDCl_3$) spectrum of **2a**.

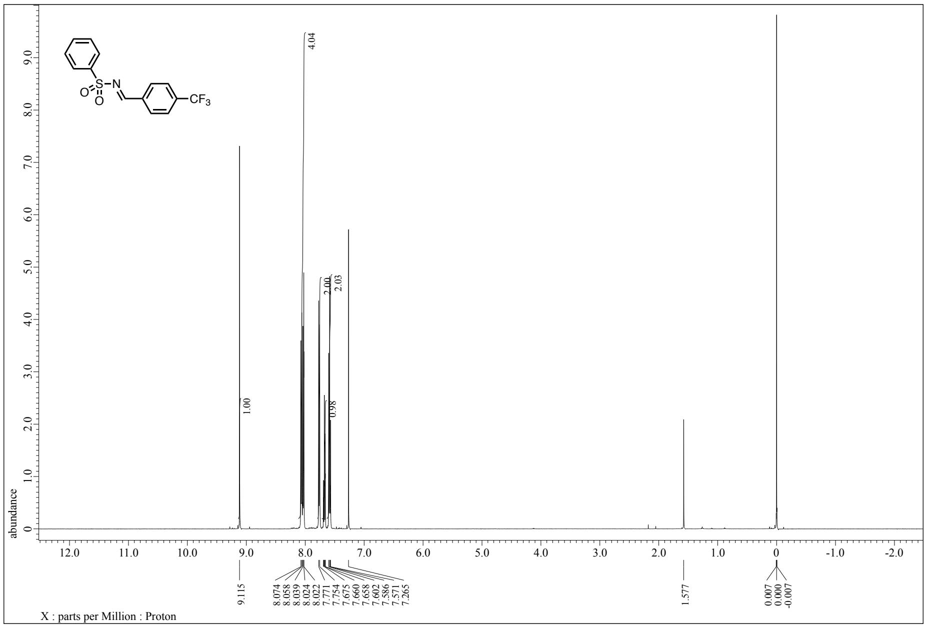

Supplementary Figure 8. ^{1}H NMR (500 MHz $CDCl_3$) spectrum of **2b**.

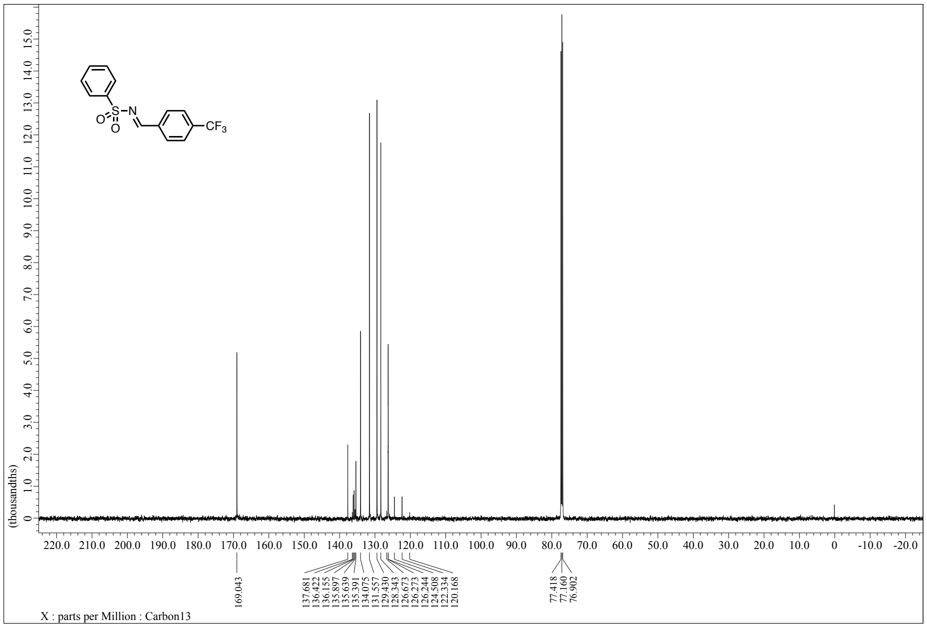

Supplementary Figure 9. ^{13}C NMR (126 MHz $CDCl_3$) spectrum of **2b**.

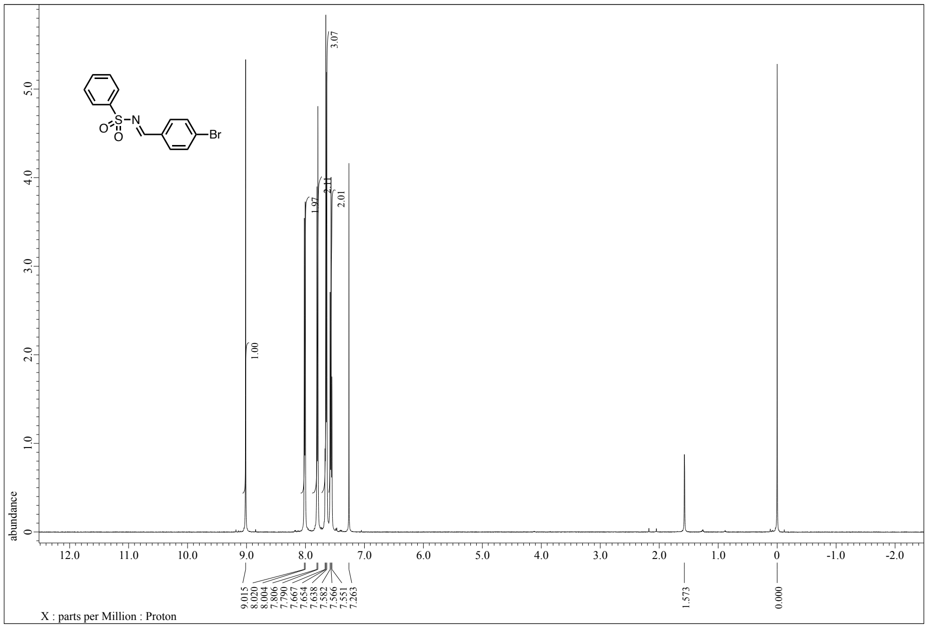

Supplementary Figure 10. ^{1}H NMR (500 MHz $CDCl_3$) spectrum of **2c**.

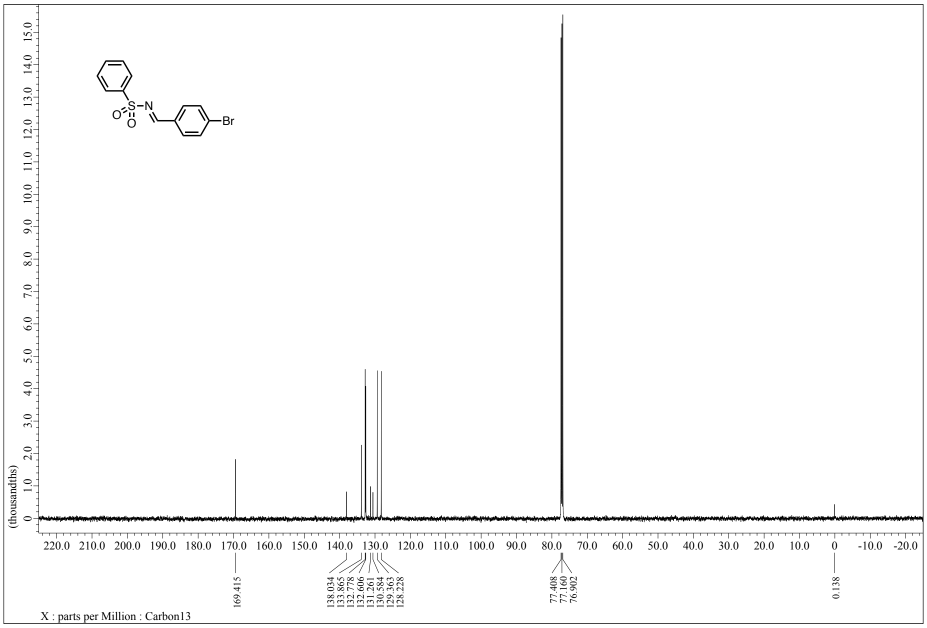

Supplementary Figure 11. ^{13}C NMR (126 MHz $CDCl_3$) spectrum of **2c**.

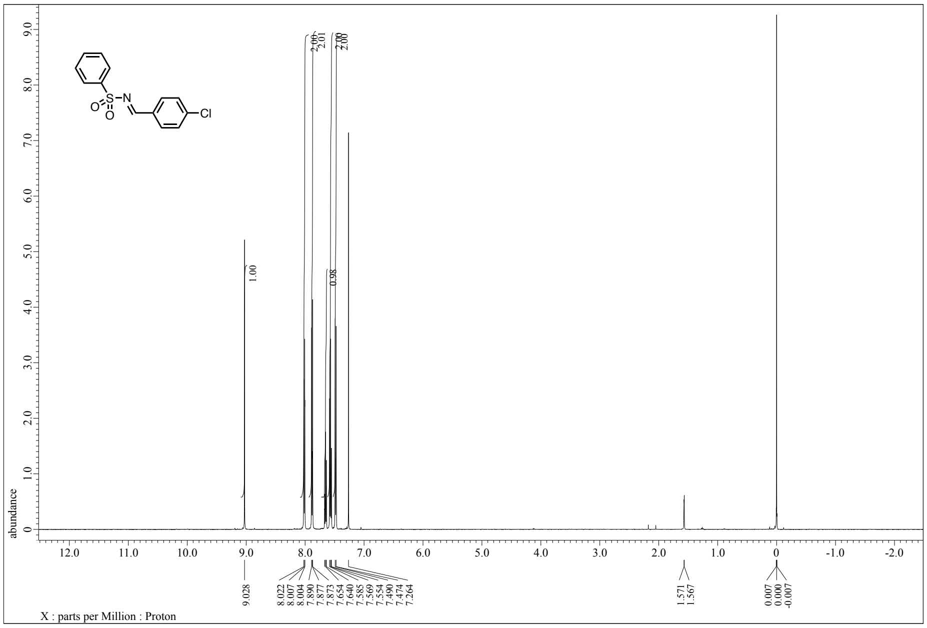

Supplementary Figure 12. ^{1}H NMR (500 MHz $CDCl_3$) spectrum of **2d**.

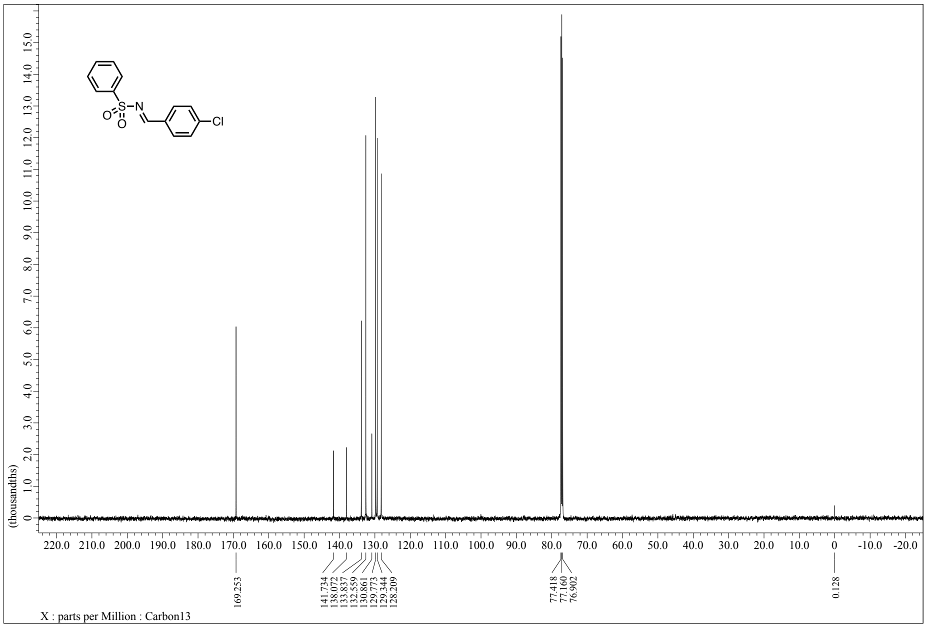

Supplementary Figure 13. ^{13}C NMR (126 MHz $CDCl_3$) spectrum of **2d**.

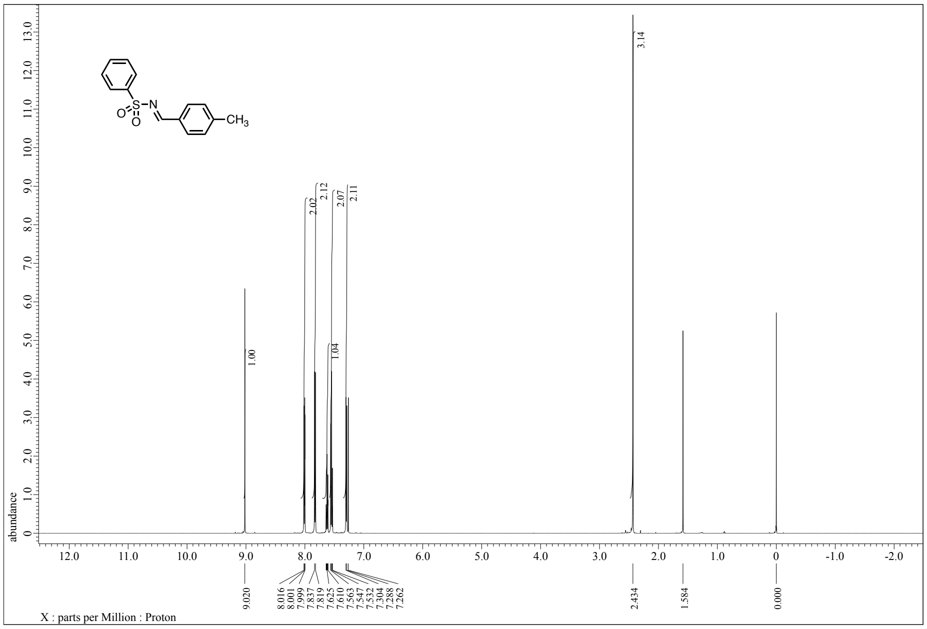

Supplementary Figure 14. ^{1}H NMR (500 MHz $CDCl_3$) spectrum of **2e**.

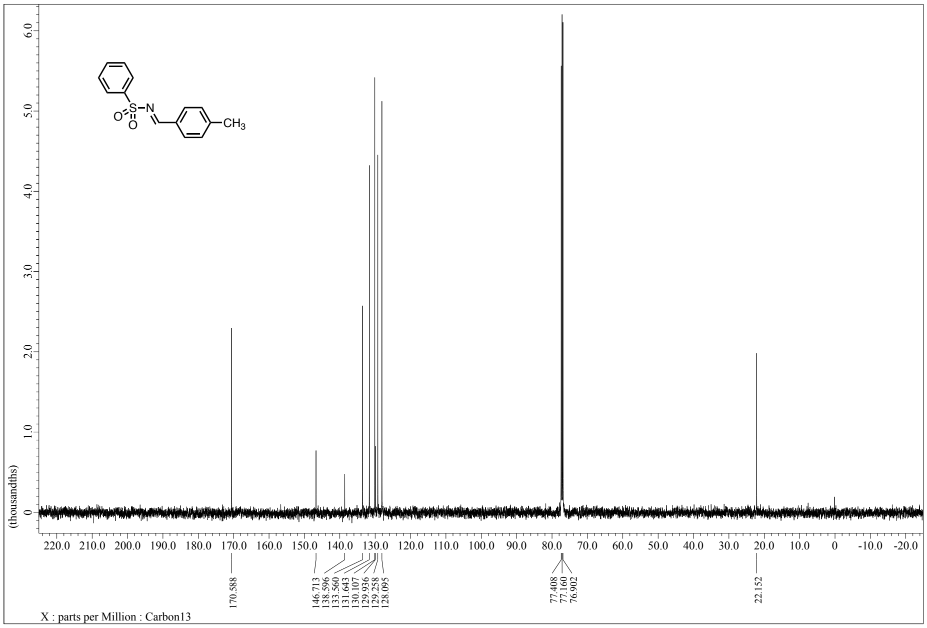

Supplementary Figure 15. ^{13}C NMR (126 MHz $CDCl_3$) spectrum of **2e**.

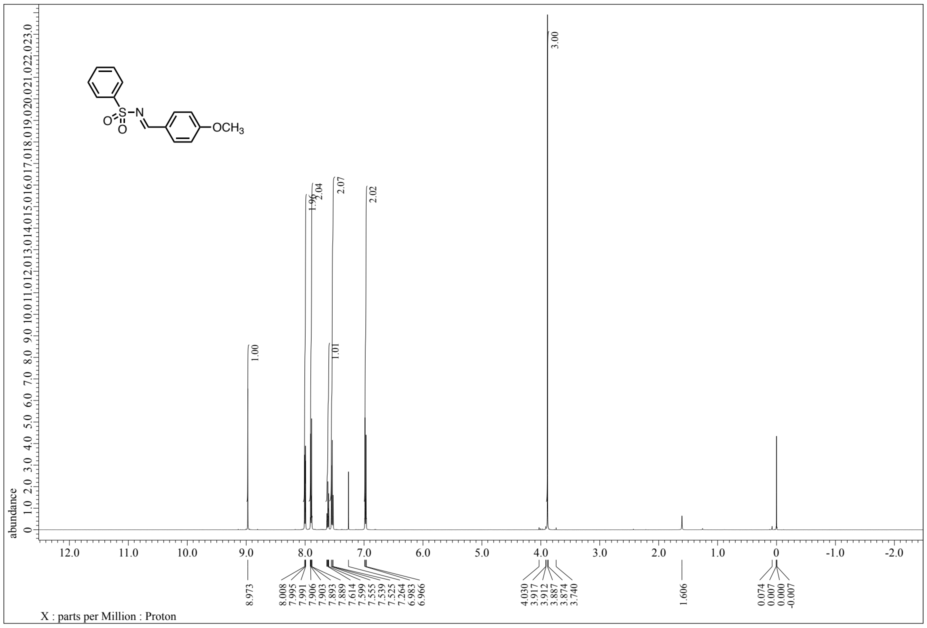

Supplementary Figure 16. ^{1}H NMR (500 MHz $CDCl_3$) spectrum of **2f**.

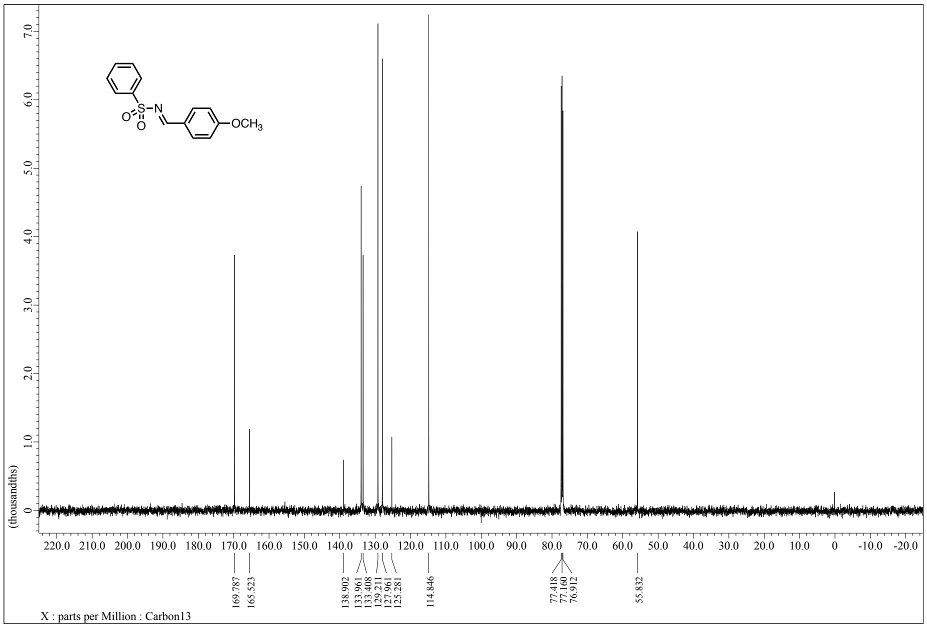

Supplementary Figure 17. ^{13}C NMR (126 MHz $CDCl_3$) spectrum of **2f**.

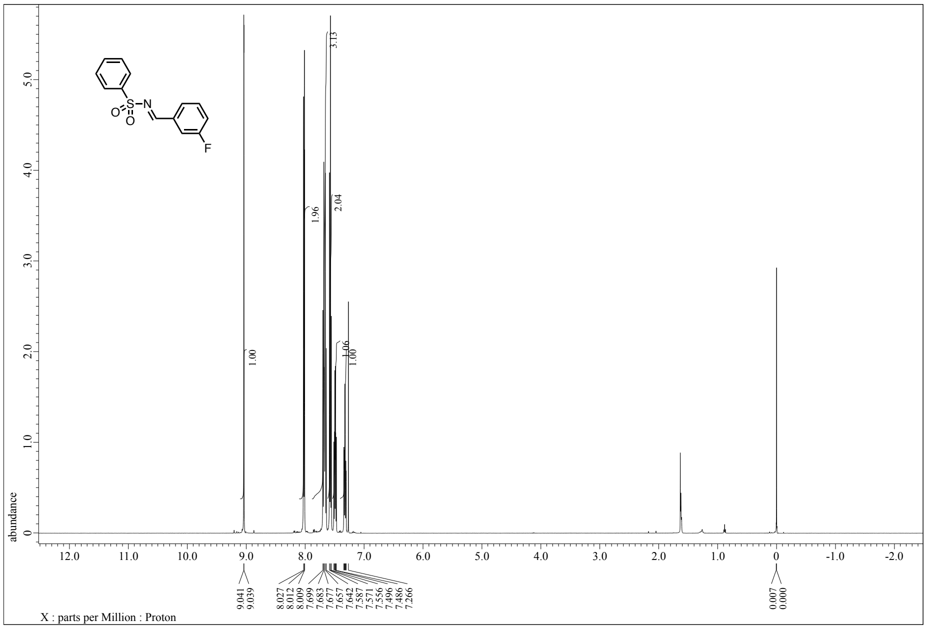

Supplementary Figure 18. ^{1}H NMR (500 MHz CDCl$_3$) spectrum of **2g**.

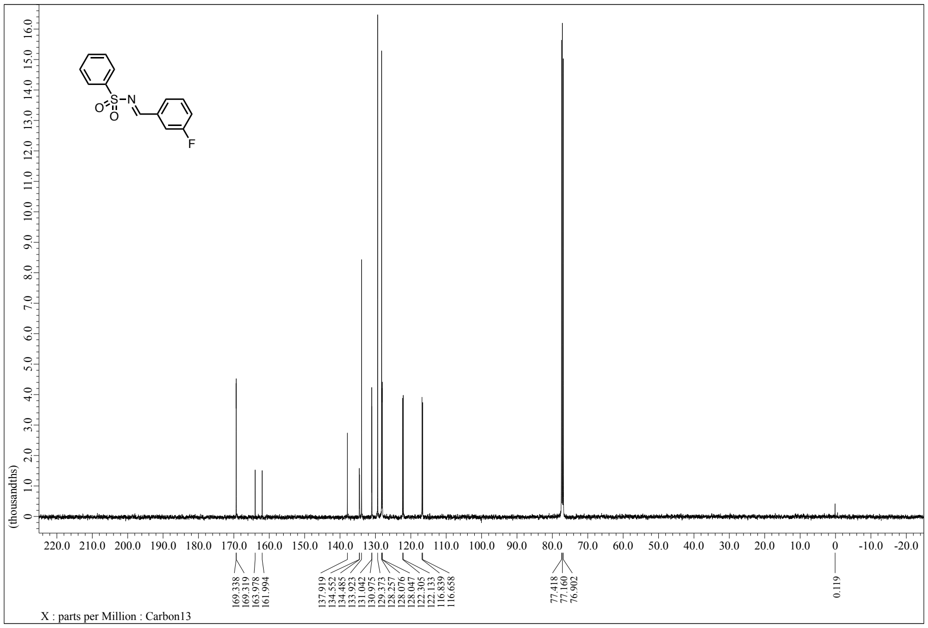

Supplementary Figure 19. ^{13}C NMR (126 MHz CDCl$_3$) spectrum of **2g**.

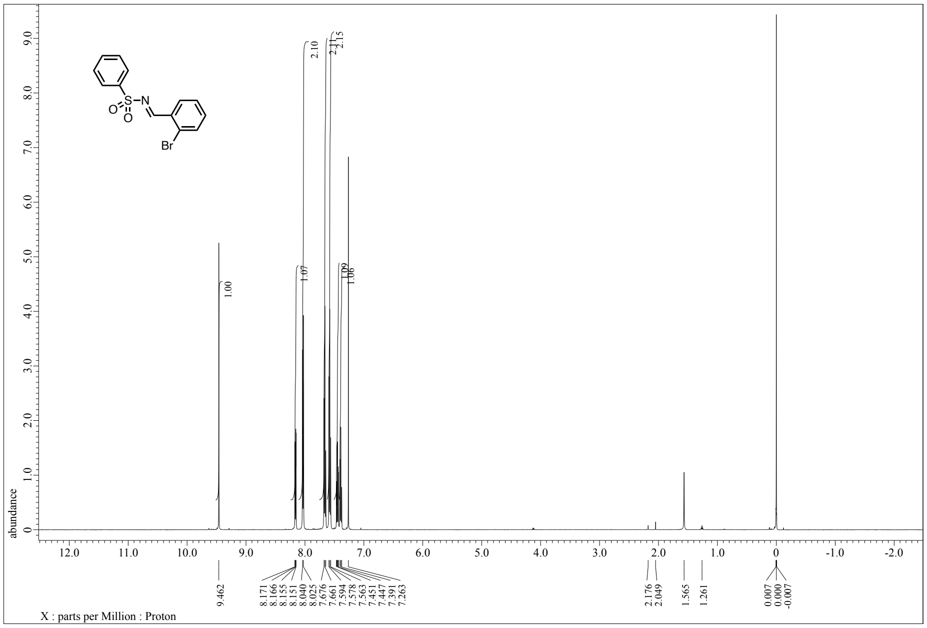

Supplementary Figure 20. ^{1}H NMR (500 MHz $CDCl_3$) spectrum of **2h**.

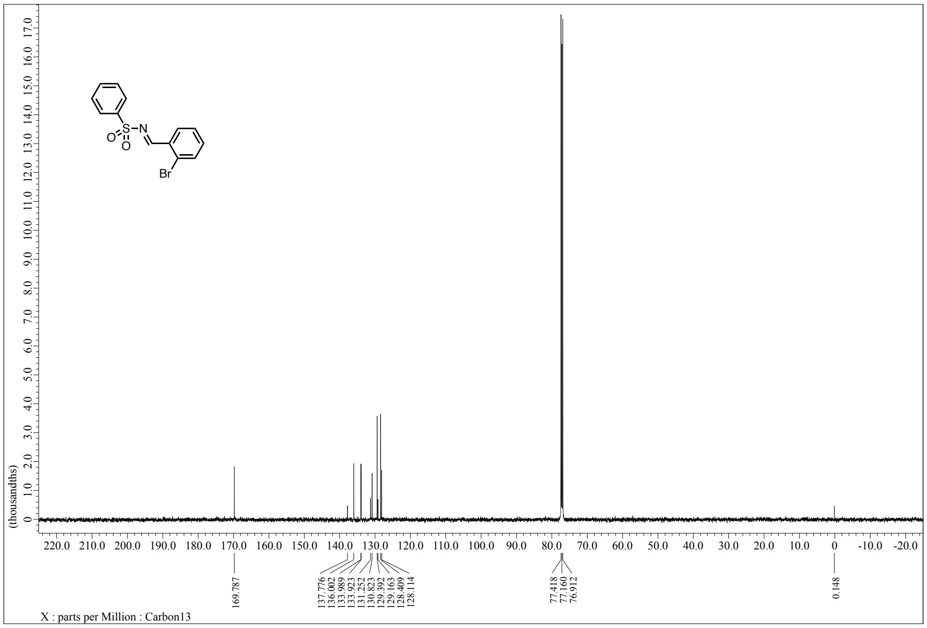

Supplementary Figure 21. ^{13}C NMR (126 MHz $CDCl_3$) spectrum of **2h**.

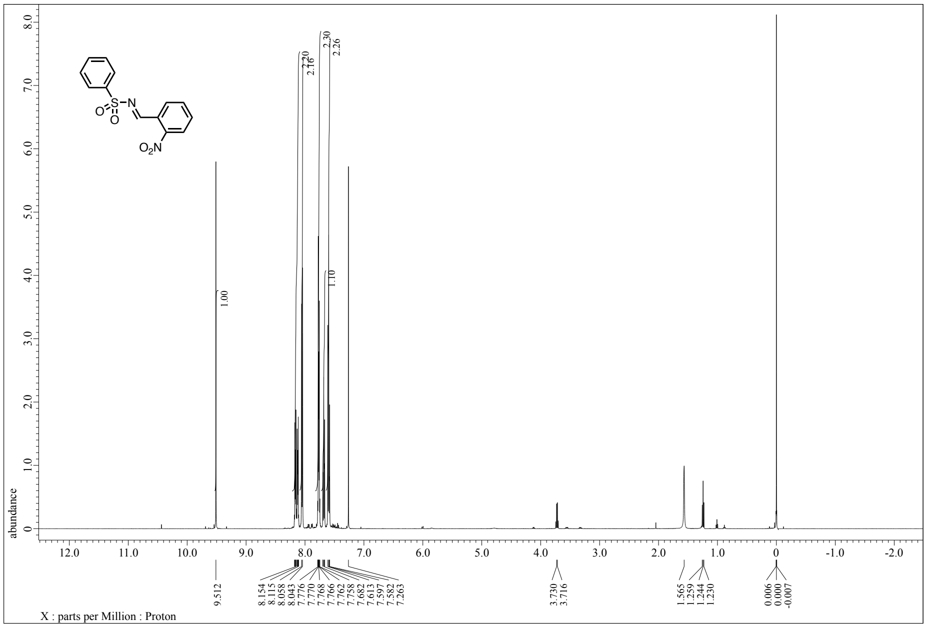

Supplementary Figure 22. ^{1}H NMR (500 MHz CDCl$_3$) spectrum of **2i**.

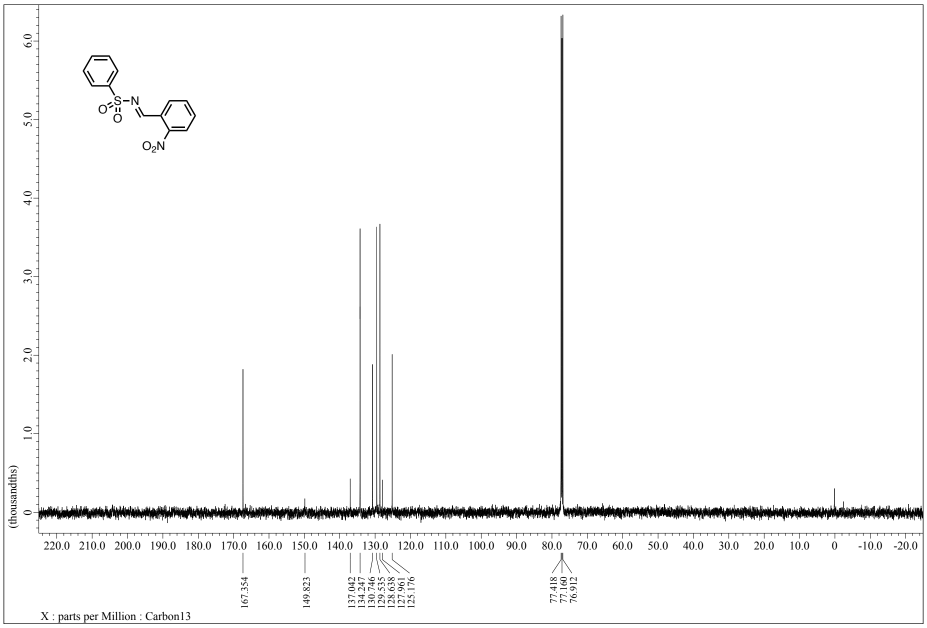

Supplementary Figure 23. ^{13}C NMR (126 MHz CDCl$_3$) spectrum of **2i**.

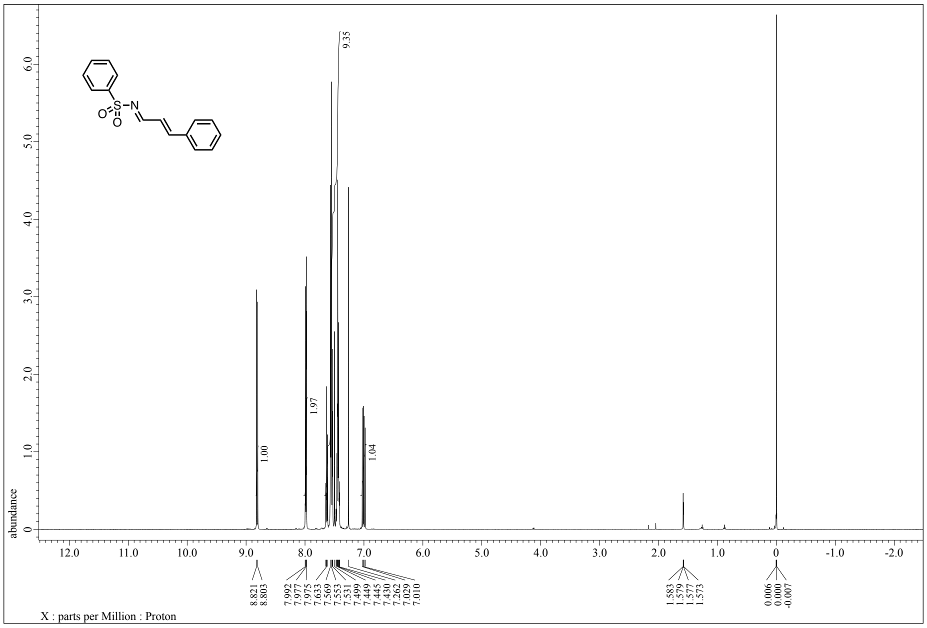

Supplementary Figure 24. ^{1}H NMR (500 MHz $CDCl_3$) spectrum of **2j**.

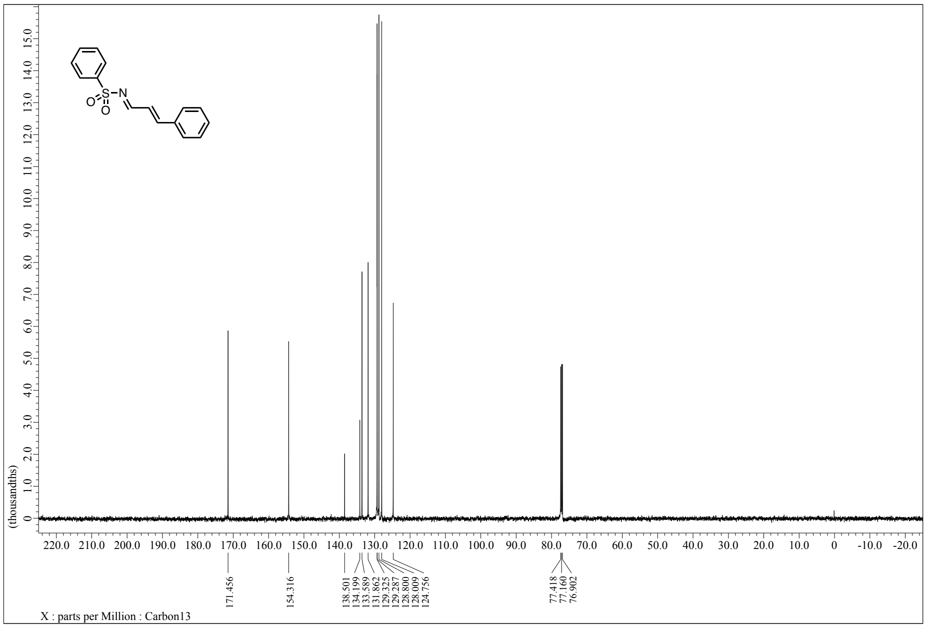

Supplementary Figure 25. ^{13}C NMR (126 MHz $CDCl_3$) spectrum of **2j**.

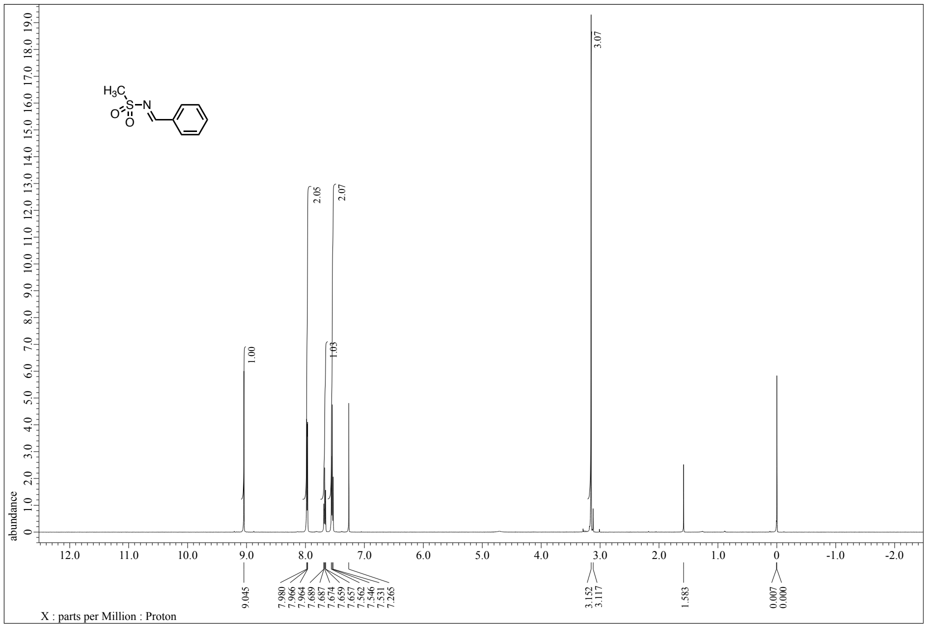

Supplementary Figure 26. ^{1}H NMR (500 MHz $CDCl_3$) spectrum of **2k**.

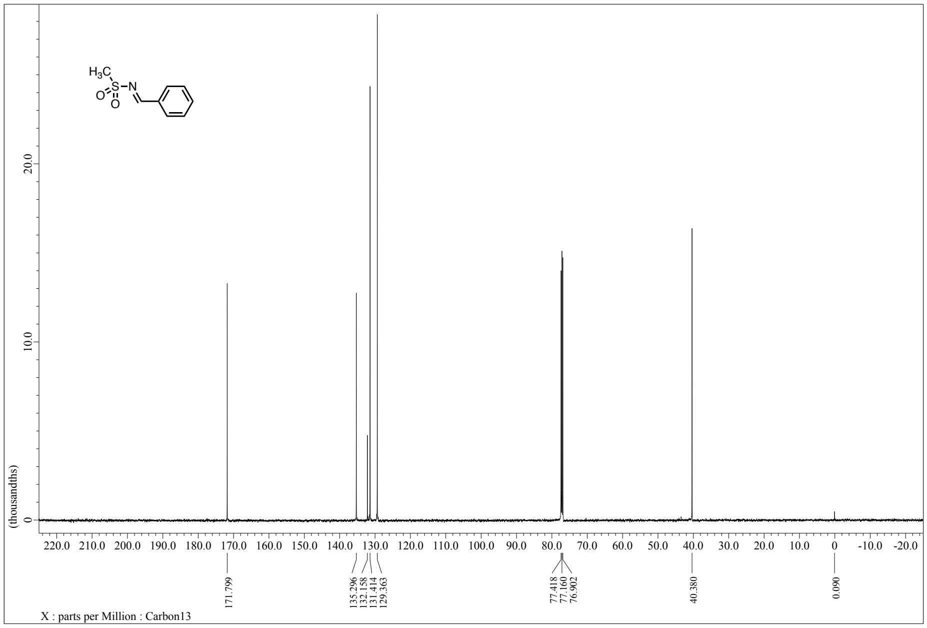

Supplementary Figure 27. ^{13}C NMR (126 MHz $CDCl_3$) spectrum of **2k**.

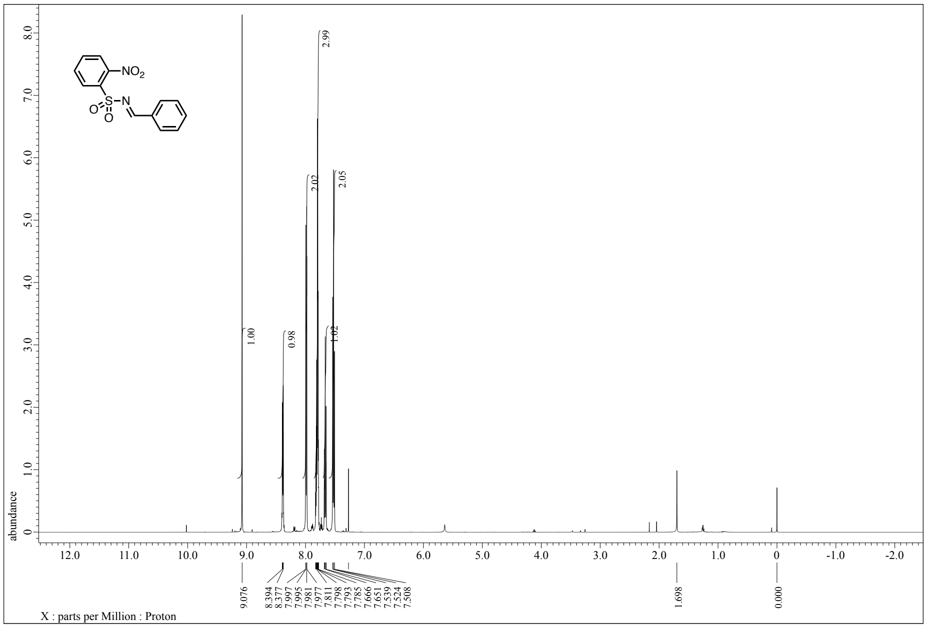

Supplementary Figure 28. ^{1}H NMR (500 MHz $CDCl_3$) spectrum of **2l**.

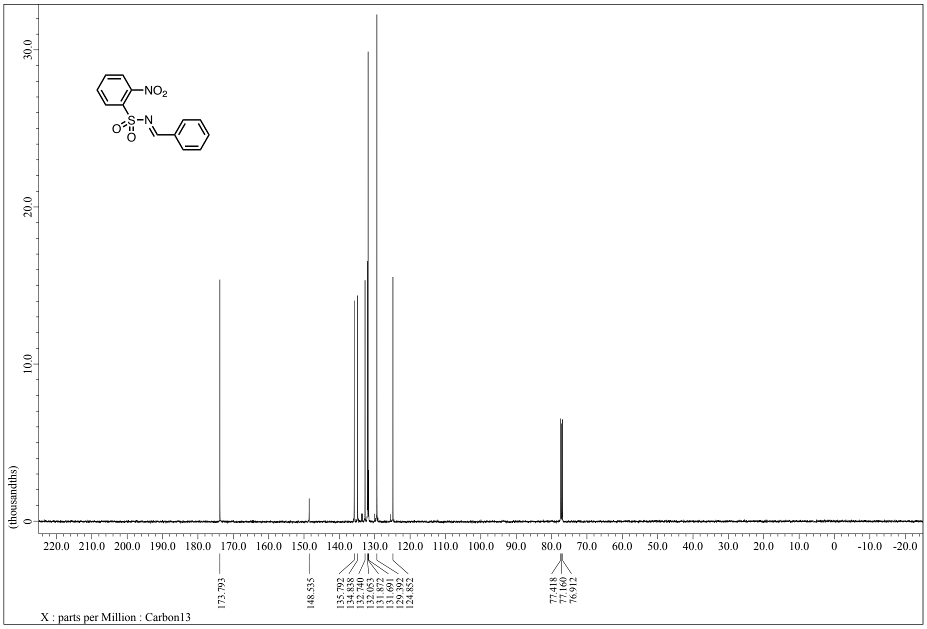

Supplementary Figure 29. ^{13}C NMR (126 MHz $CDCl_3$) spectrum of **2l**.

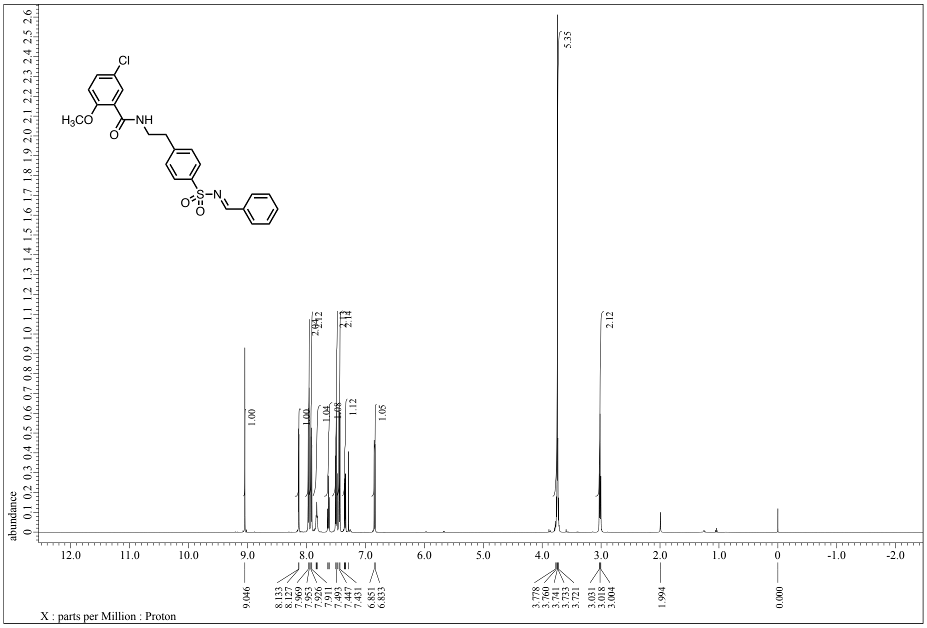

Supplementary Figure 30. ^{1}H NMR (500 MHz $CDCl_3$) spectrum of **2m**.

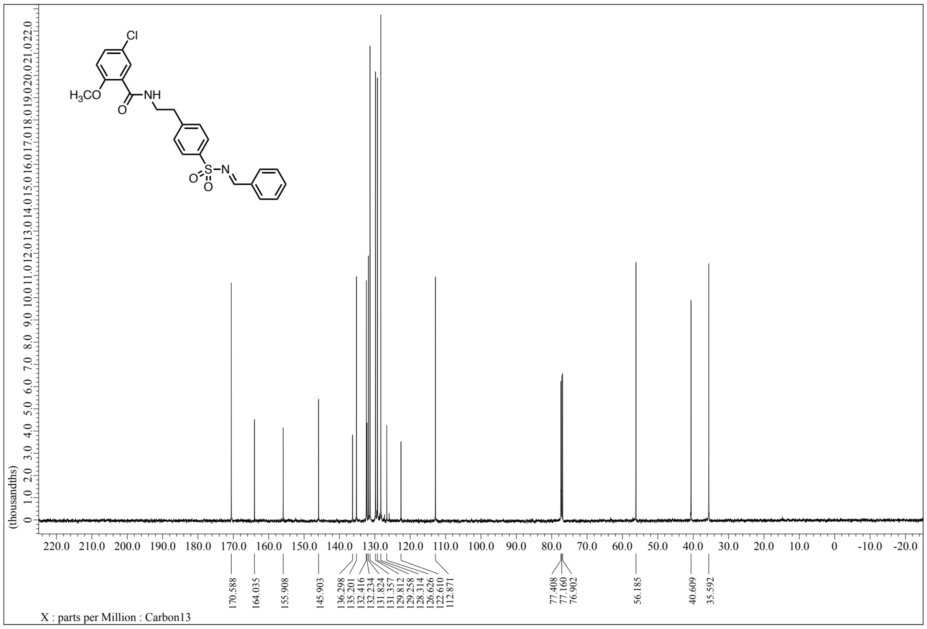

Supplementary Figure 31. ^{13}C NMR (126 MHz $CDCl_3$) spectrum of **2m**.

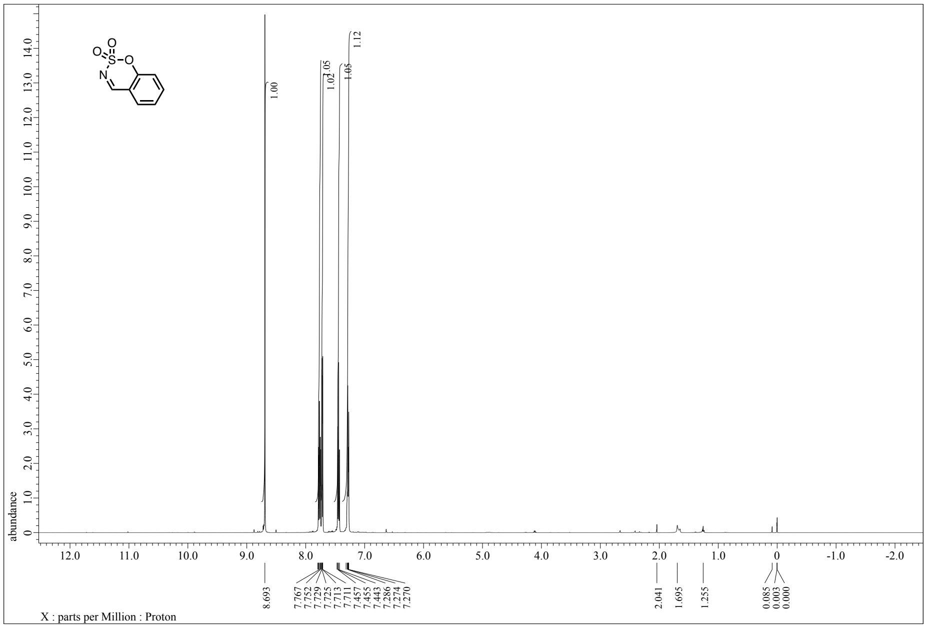

Supplementary Figure 32. ^{1}H NMR (500 MHz $CDCl_3$) spectrum of **2n**.

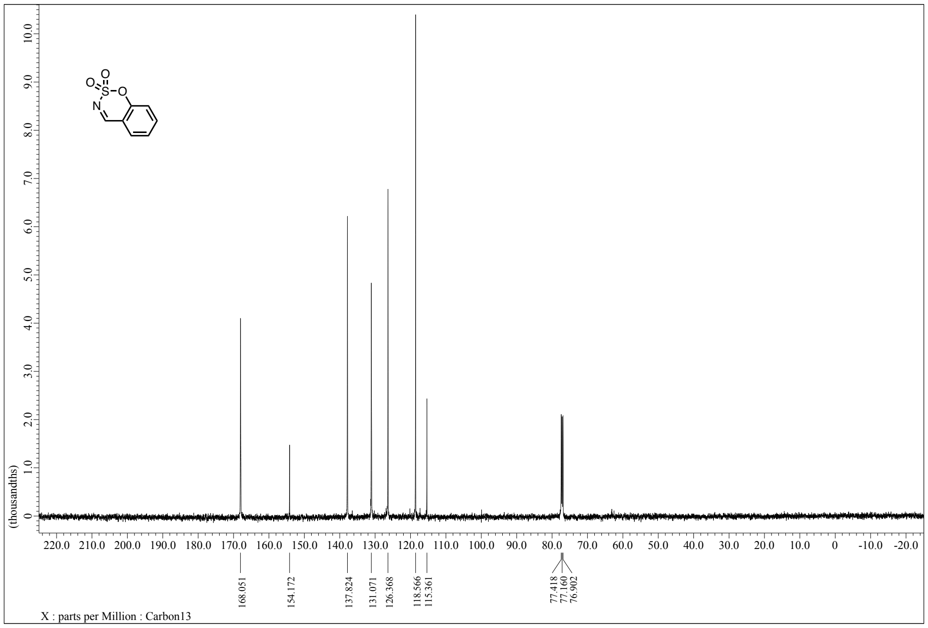

Supplementary Figure 33. ^{13}C NMR (126 MHz $CDCl_3$) spectrum of **2n**.

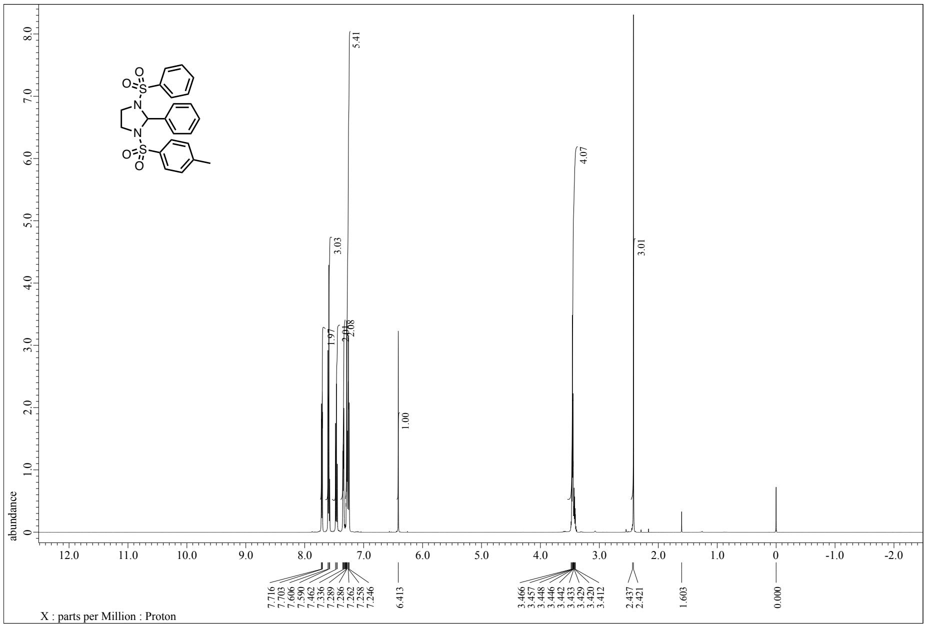

Supplementary Figure 34. ^{1}H NMR (500 MHz $CDCl_3$) spectrum of **3a**.

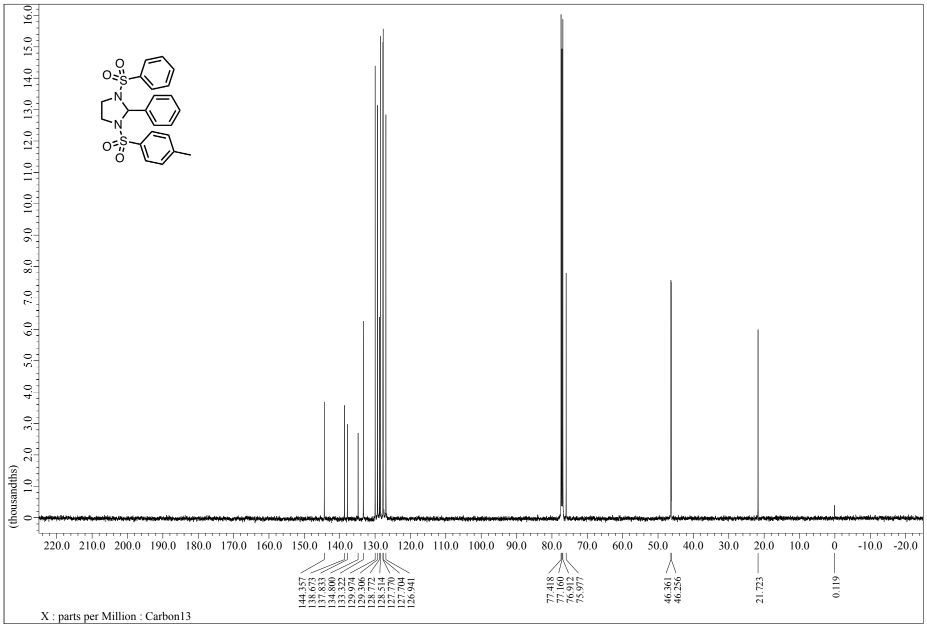

Supplementary Figure 35. ^{13}C NMR (126 MHz $CDCl_3$) spectrum of **3a**.

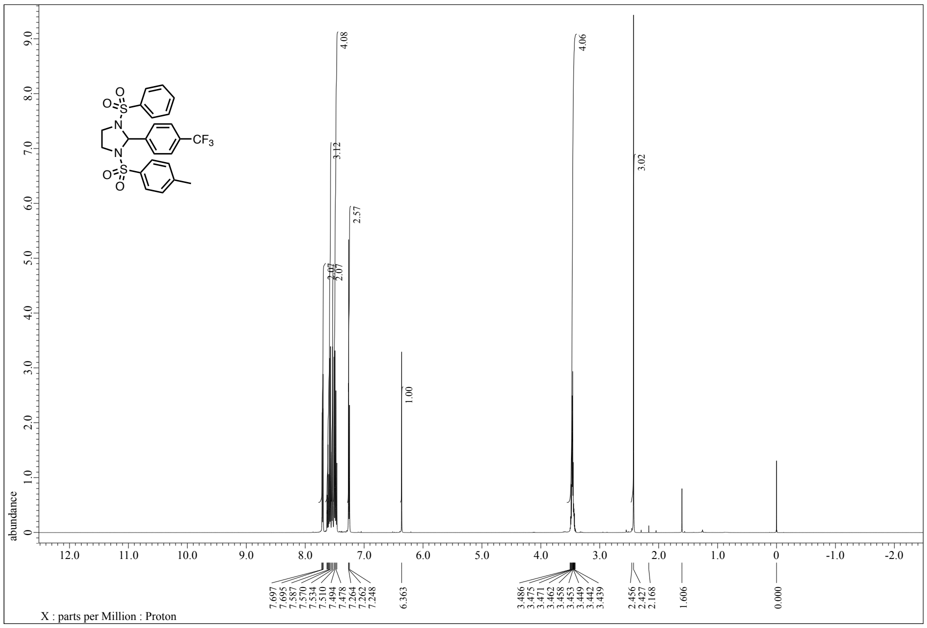

Supplementary Figure 36. ^{1}H NMR (500 MHz $CDCl_3$) spectrum of **3b**.

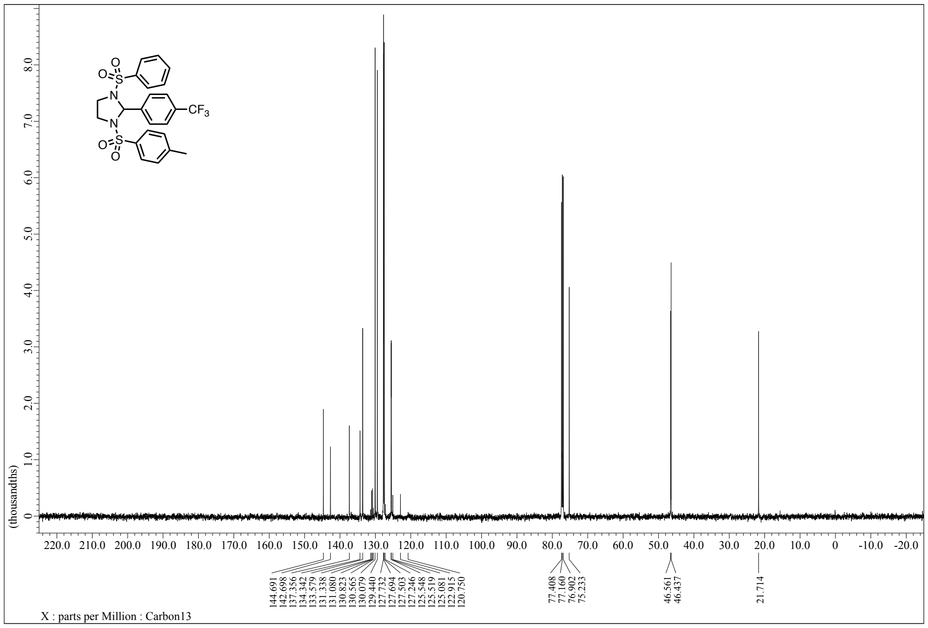

Supplementary Figure 37. ^{13}C NMR (126 MHz $CDCl_3$) spectrum of **3b**.

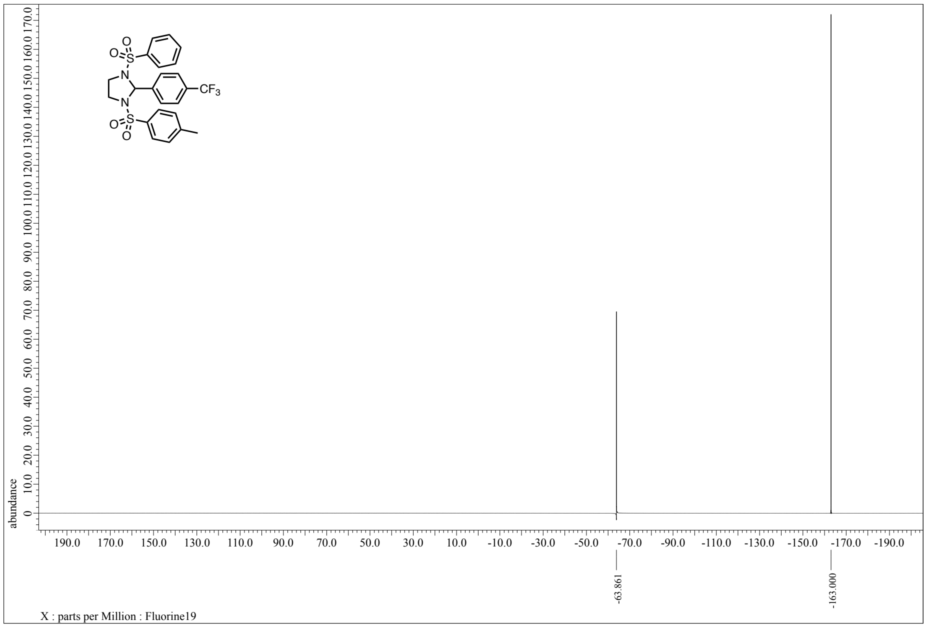

Supplementary Figure 38. ^{19}F NMR (471 MHz $CDCl_3$) spectrum of **3b**. (C_6F_6 was added to the sample.)

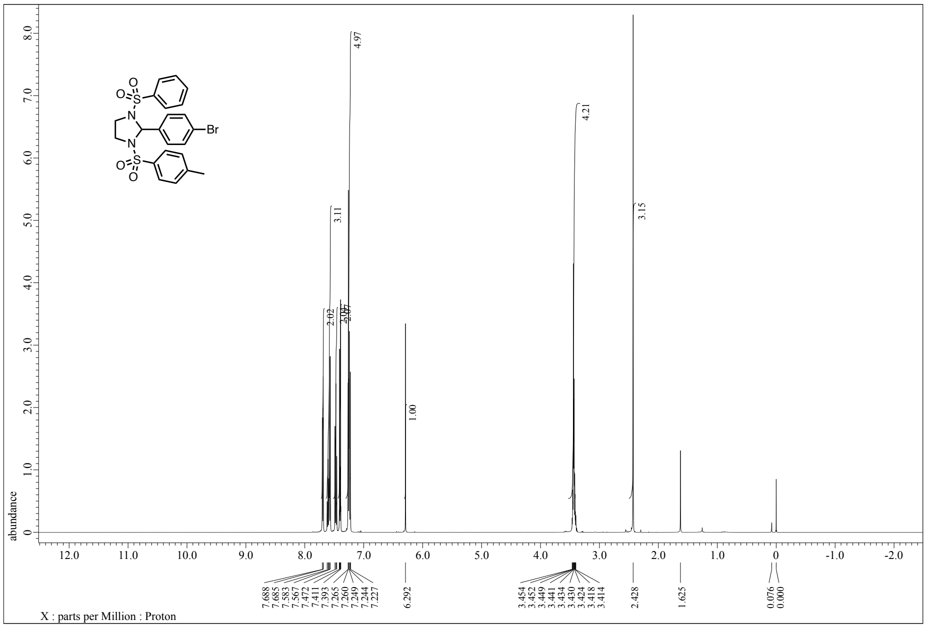

Supplementary Figure 39. ^{1}H NMR (500 MHz $CDCl_3$) spectrum of **3c**.

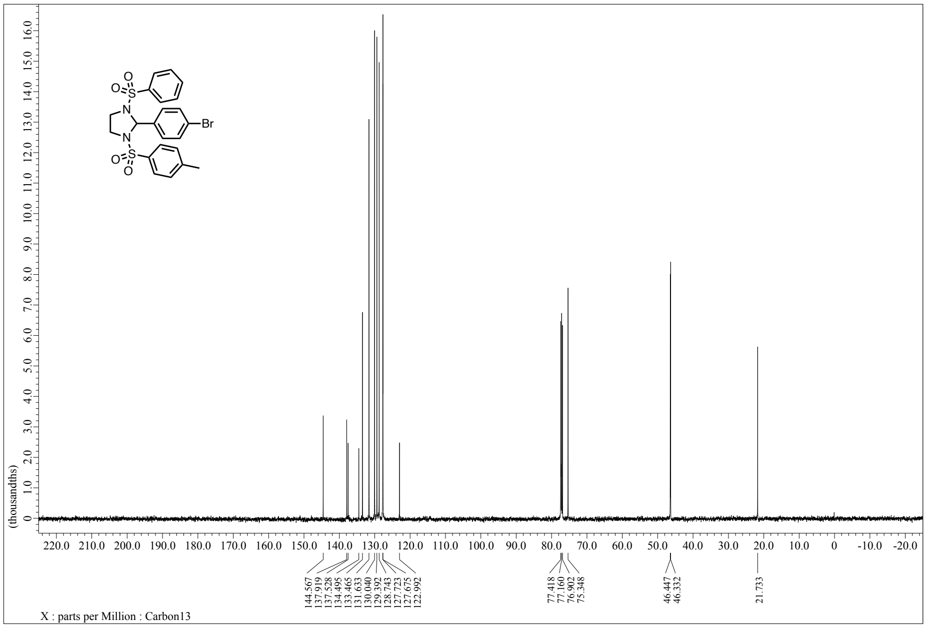

Supplementary Figure 40. ^{13}C NMR (126 MHz $CDCl_3$) spectrum of **3c**.

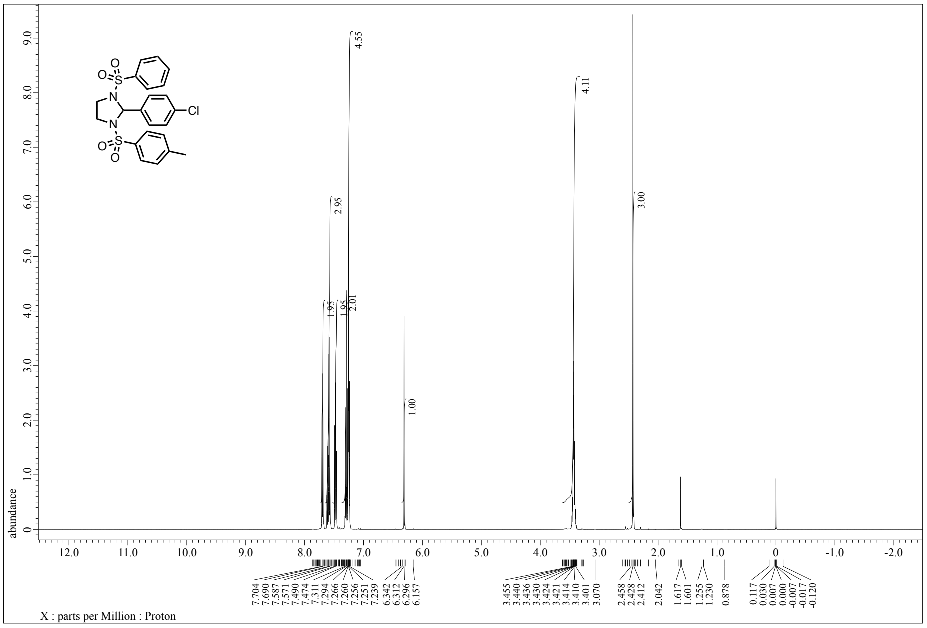

Supplementary Figure 41. ^{1}H NMR (500 MHz $CDCl_3$) spectrum of **3d**.

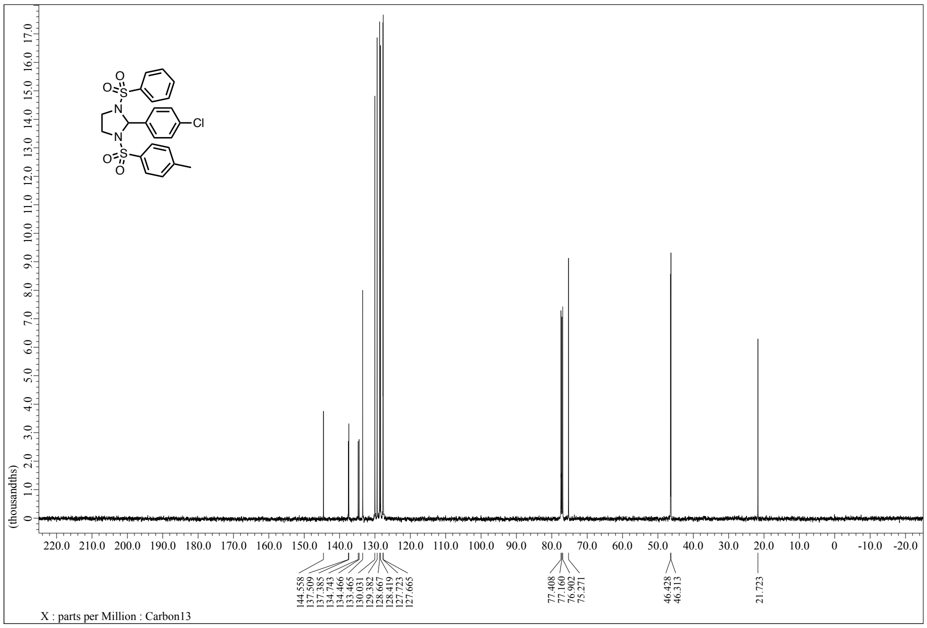

Supplementary Figure 42. ^{13}C NMR (126 MHz $CDCl_3$) spectrum of **3d**.

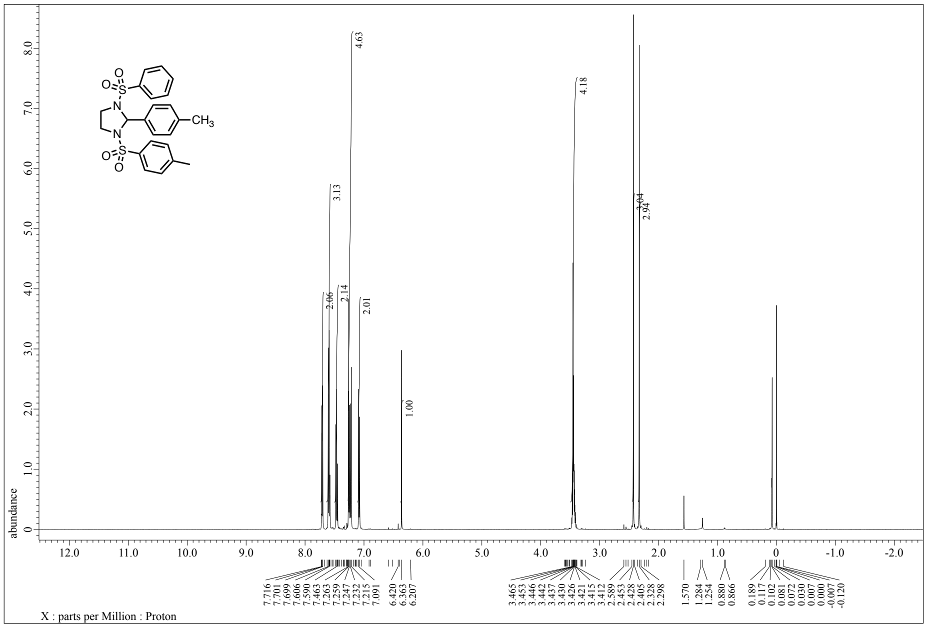

Supplementary Figure 43. ^{1}H NMR (500 MHz $CDCl_3$) spectrum of **3e**.

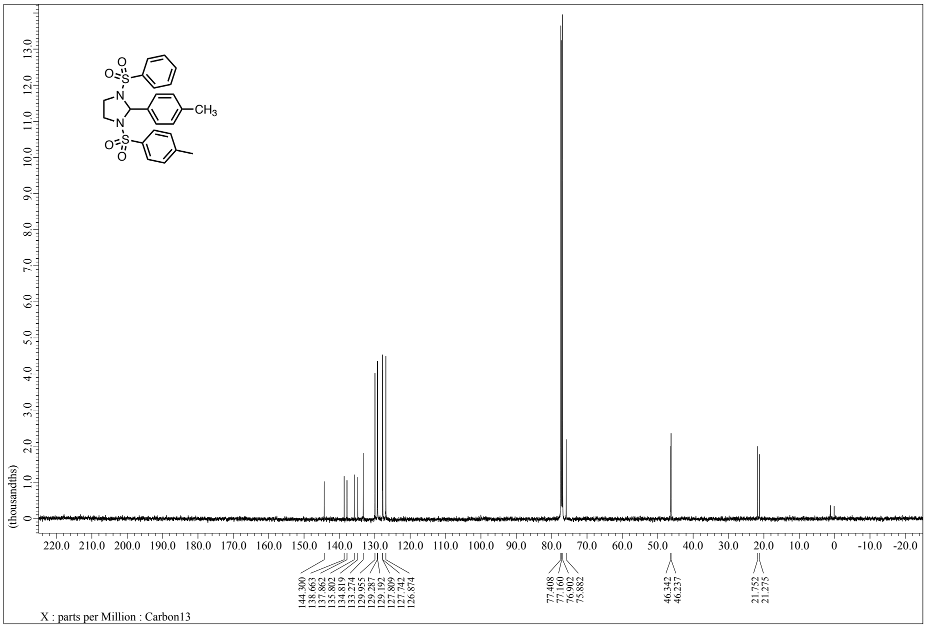

Supplementary Figure 44. ^{13}C NMR (126 MHz $CDCl_3$) spectrum of **3e**.

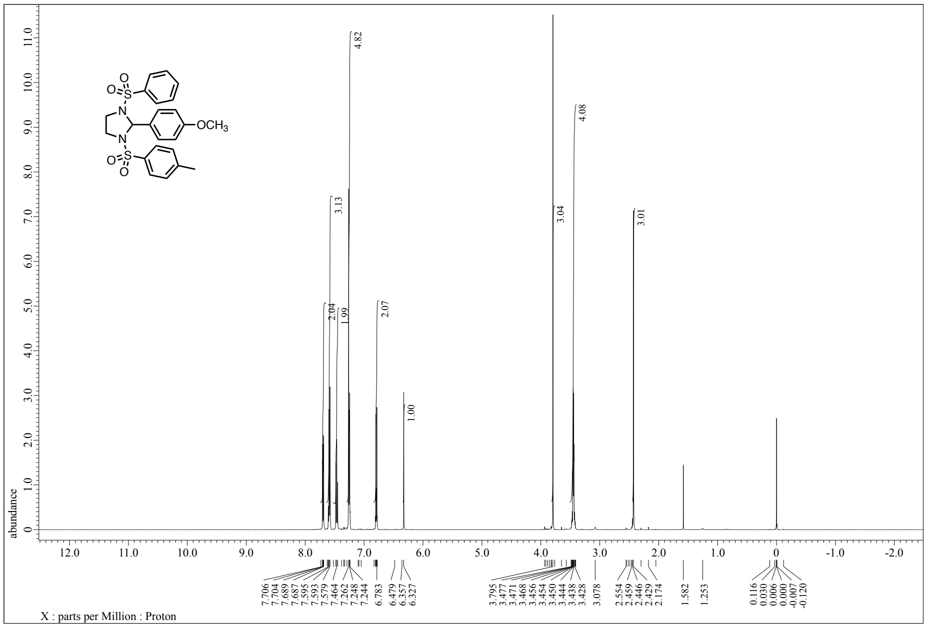

Supplementary Figure 45. ^{1}H NMR (500 MHz $CDCl_3$) spectrum of **3f**.

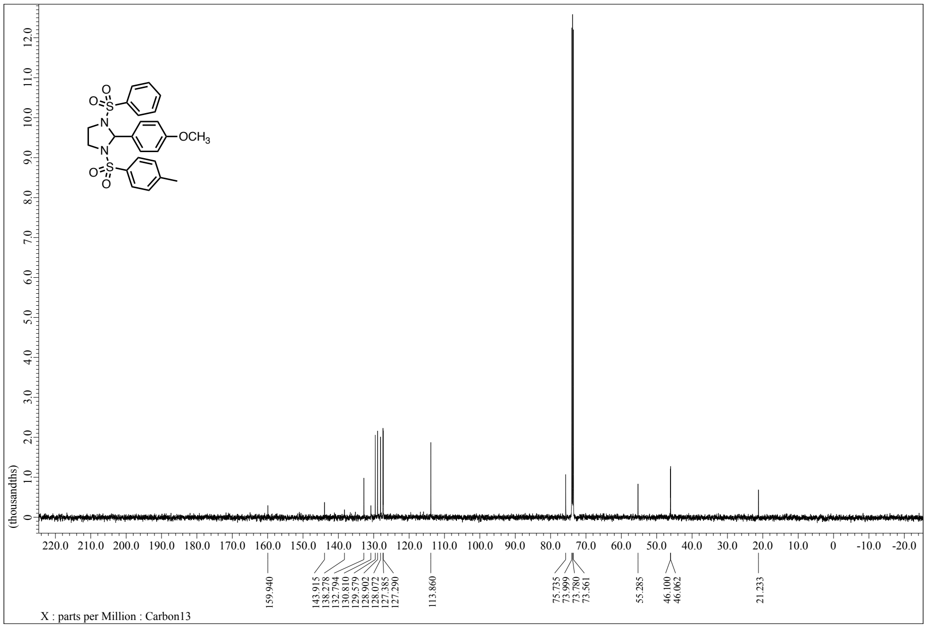

Supplementary Figure 46. ^{13}C NMR (126 MHz $C_2D_2Cl_4$ 100 °C) spectrum of **3f**.

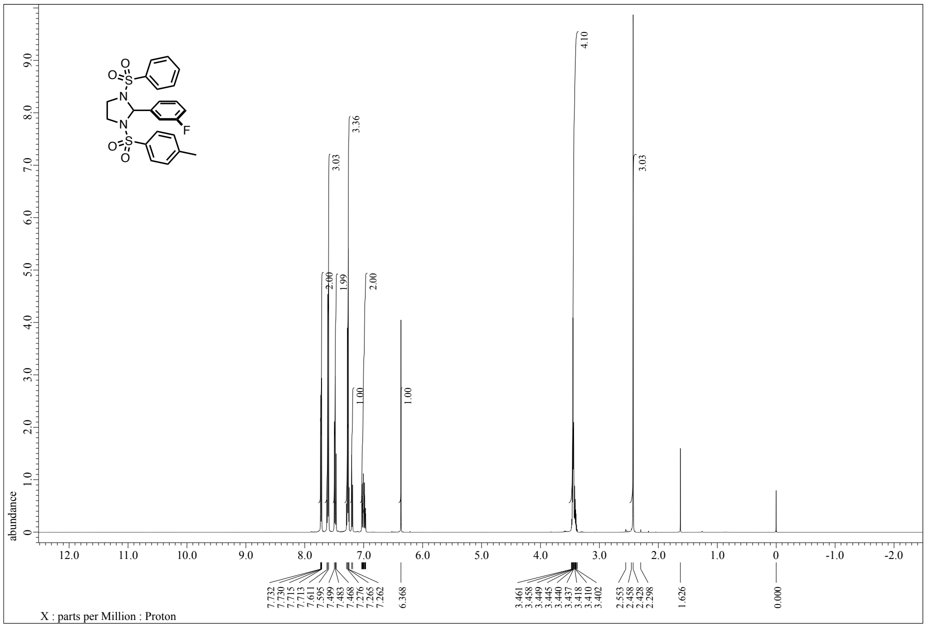

Supplementary Figure 47. ^{1}H NMR (500 MHz $CDCl_3$) spectrum of **3g**.

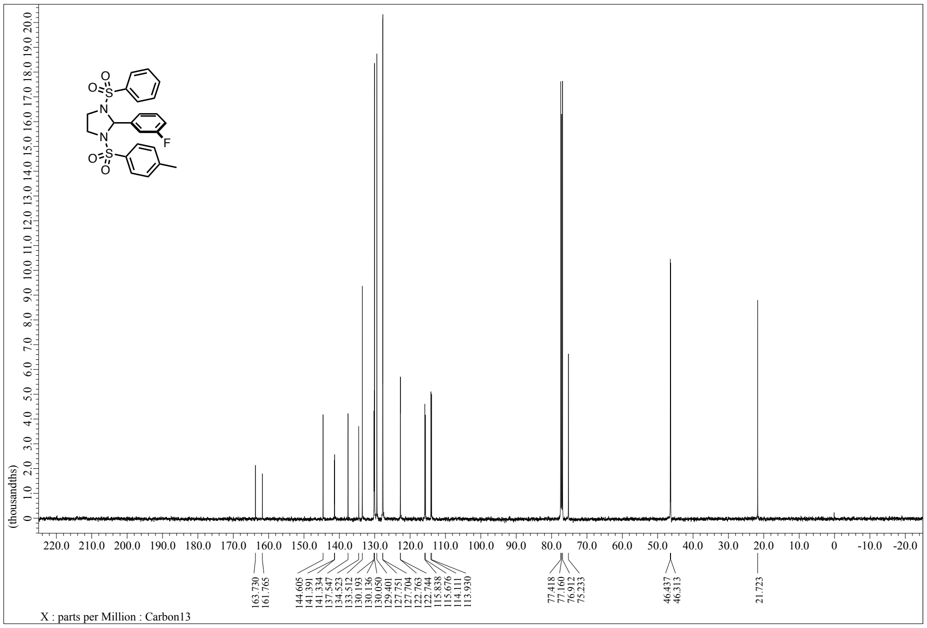

Supplementary Figure 48. ^{13}C NMR (126 MHz $CDCl_3$) spectrum of **3g**.

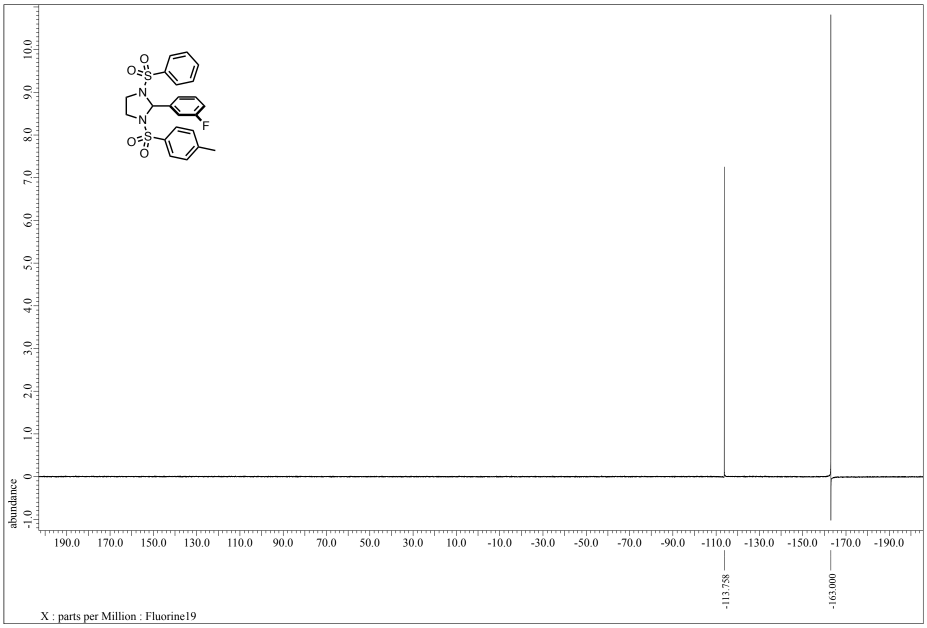

Supplementary Figure 49. ^{19}F NMR (471 MHz $CDCl_3$) spectrum of **3g**. (C_6F_6 was added to the sample.)

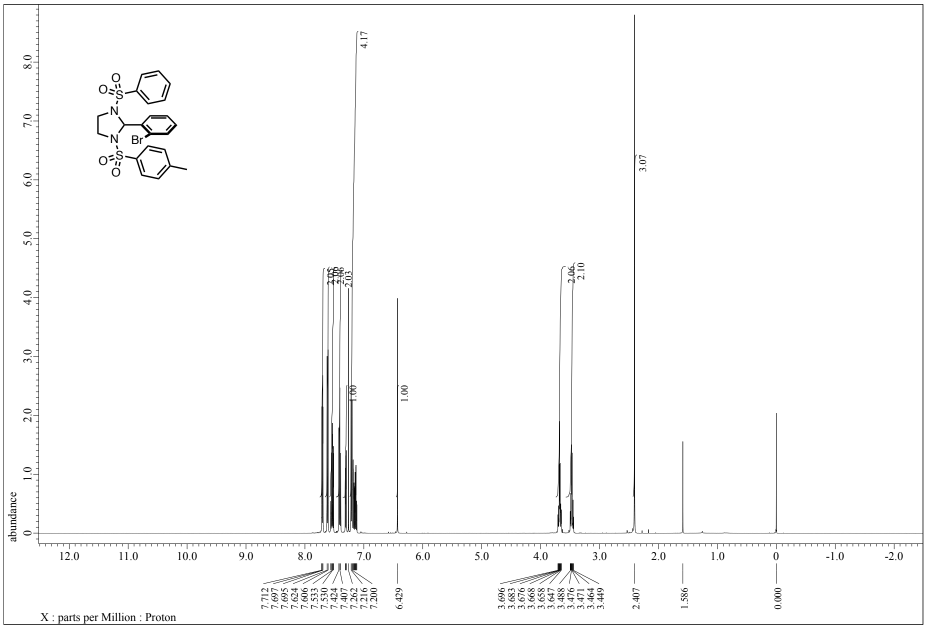

Supplementary Figure 50. ^{1}H NMR (500 MHz $CDCl_3$) spectrum of **3h**.

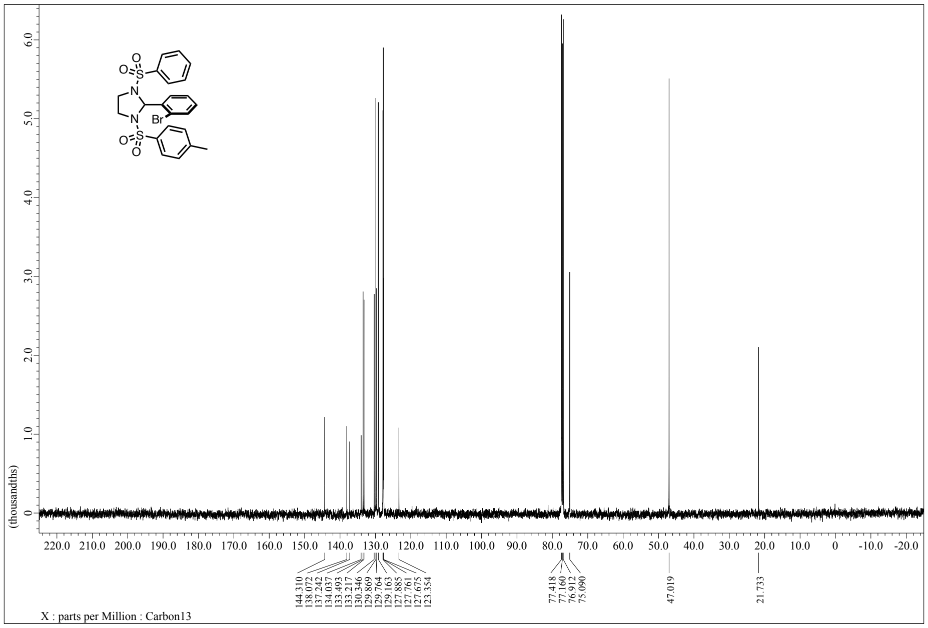

Supplementary Figure 51. ^{13}C NMR (126 MHz $CDCl_3$) spectrum of **3h**.

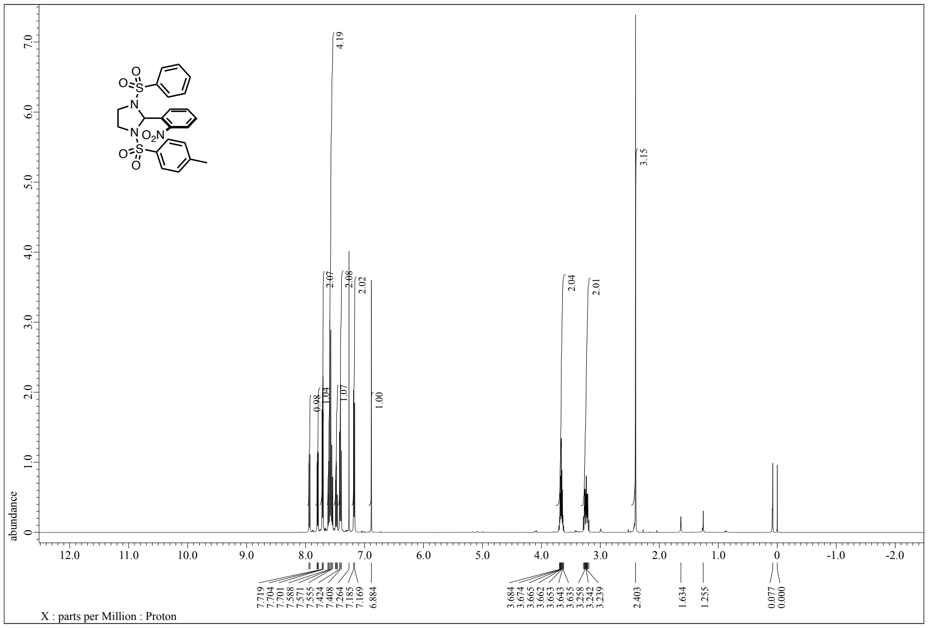

Supplementary Figure 52. ^{1}H NMR (500 MHz $CDCl_3$) spectrum of **3i**.

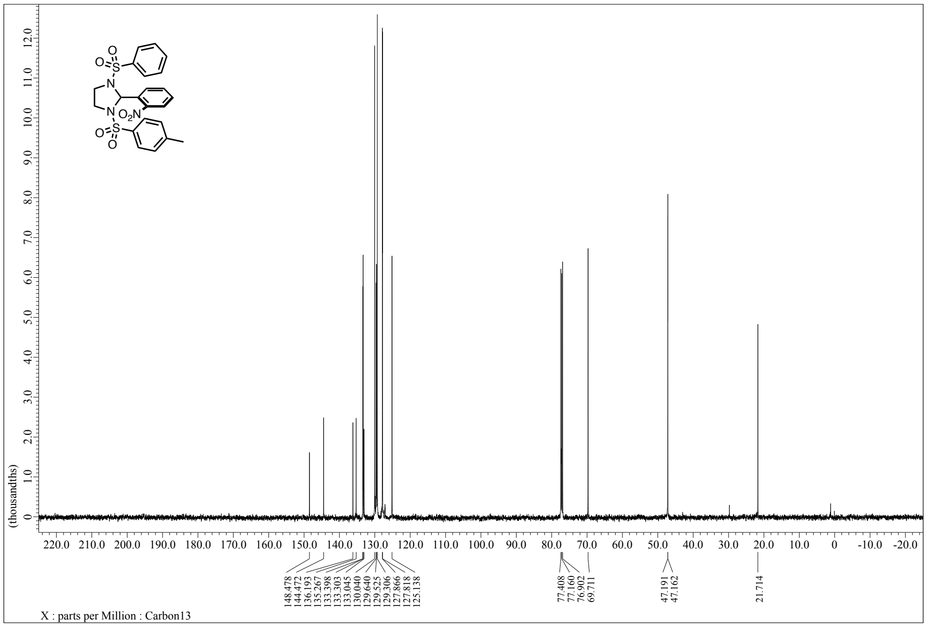

Supplementary Figure 53. ^{13}C NMR (126 MHz $CDCl_3$) spectrum of **3i**.

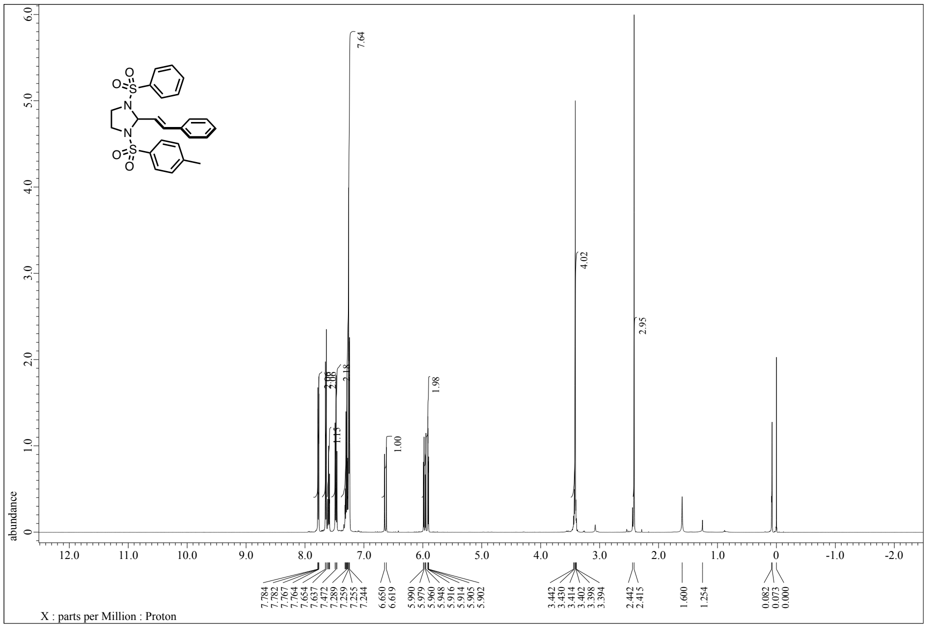

Supplementary Figure 54. ^{1}H NMR (500 MHz $CDCl_3$) spectrum of **3j**.

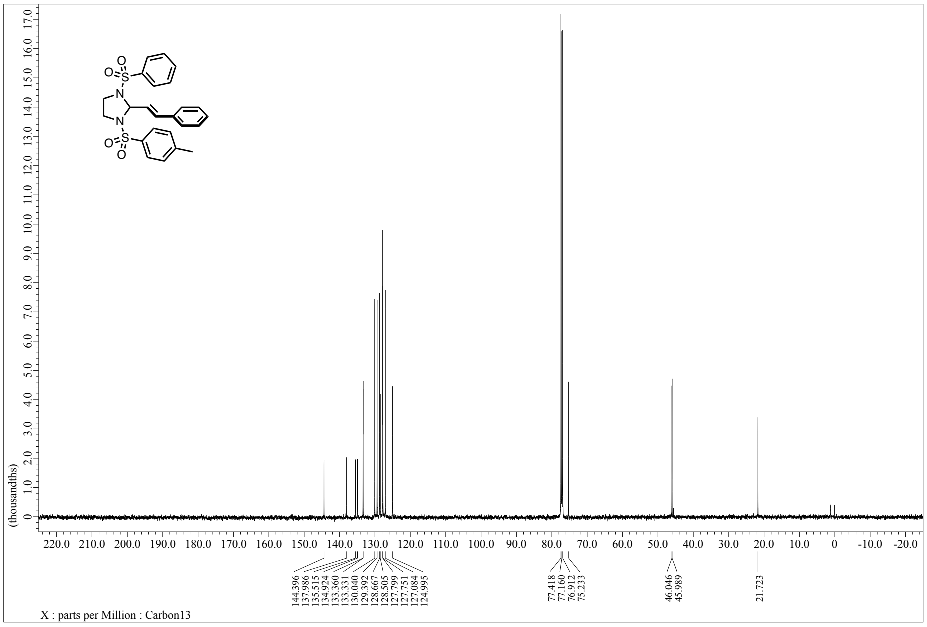

Supplementary Figure 55. ^{13}C NMR (126 MHz $CDCl_3$) spectrum of **3j**.

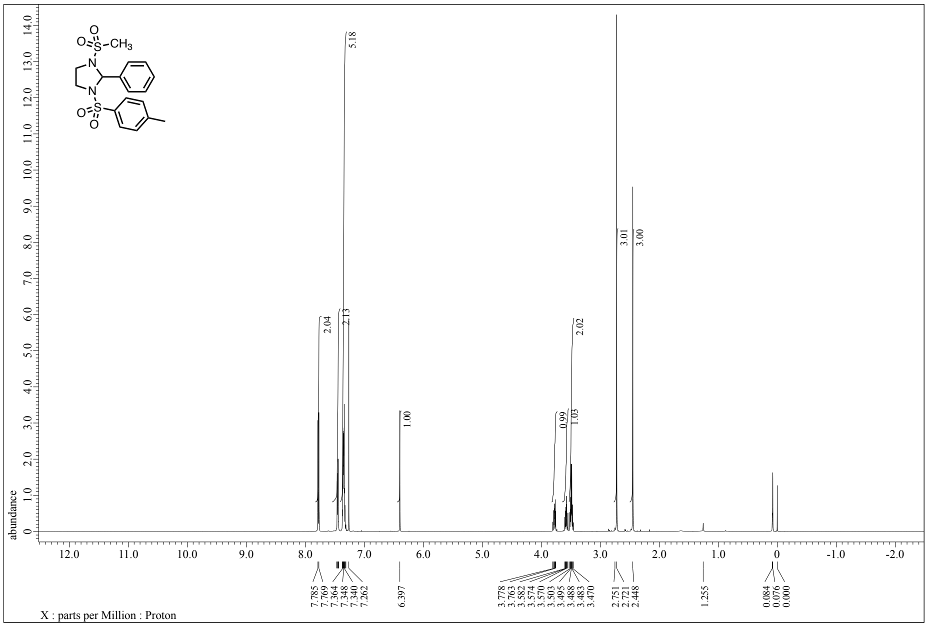

Supplementary Figure 56. ^{1}H NMR (500 MHz $CDCl_3$) spectrum of **3k**.

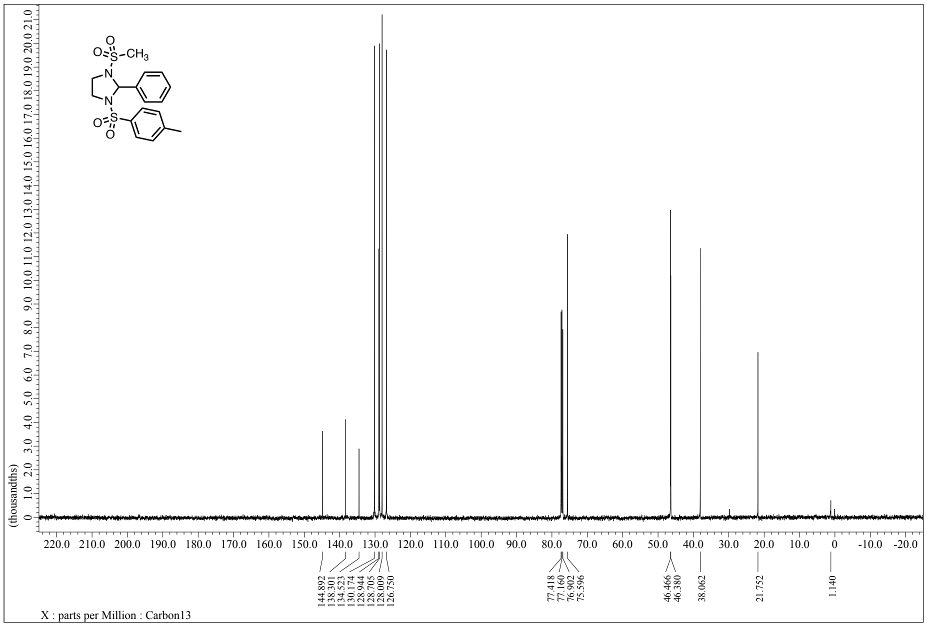

Supplementary Figure 57. ^{13}C NMR (126 MHz $CDCl_3$) spectrum of **3k**.

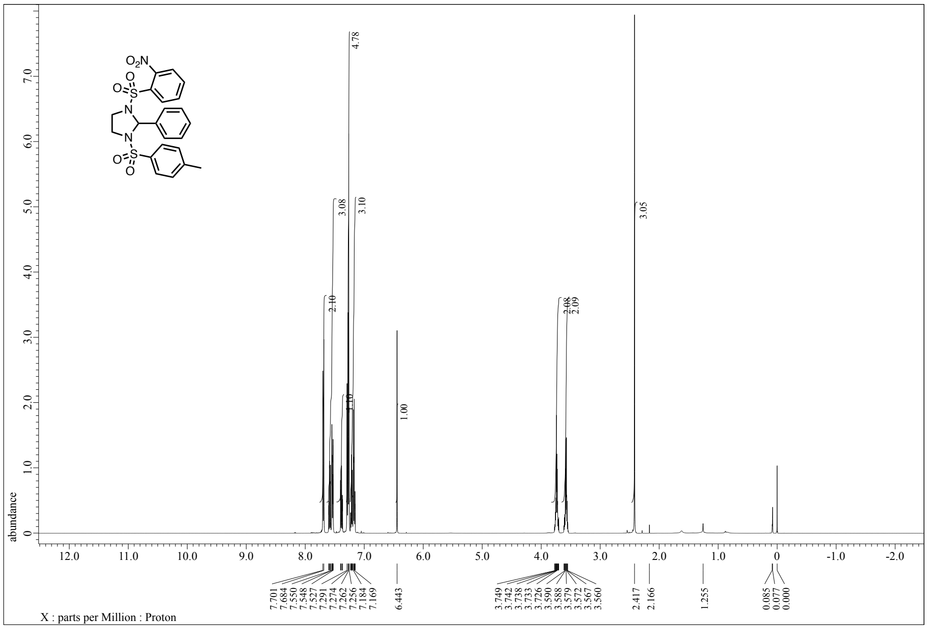

Supplementary Figure 58. ^{1}H NMR (500 MHz $CDCl_3$) spectrum of **3l**.

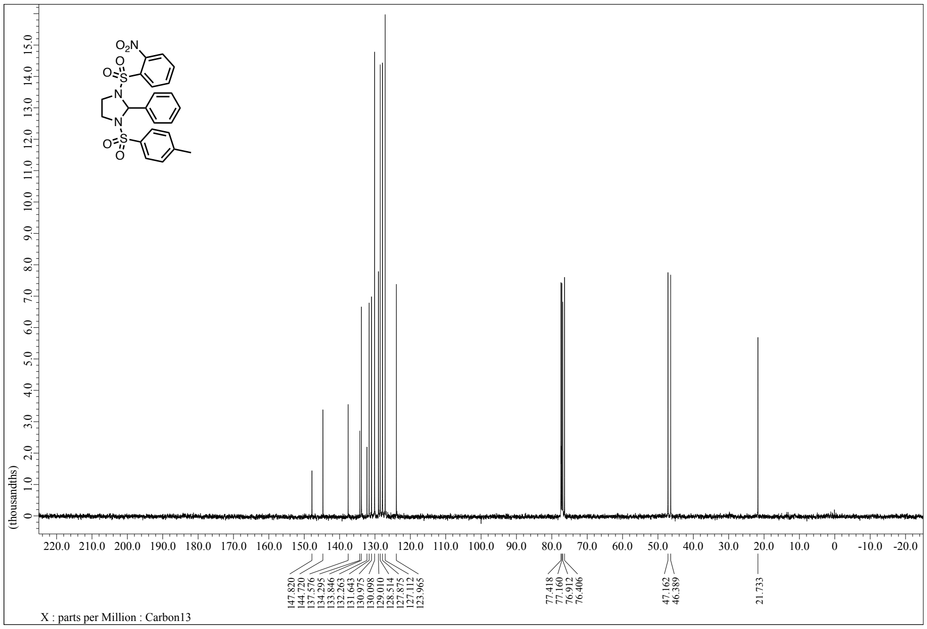

Supplementary Figure 59. ^{13}C NMR (126 MHz $CDCl_3$) spectrum of **3l**.

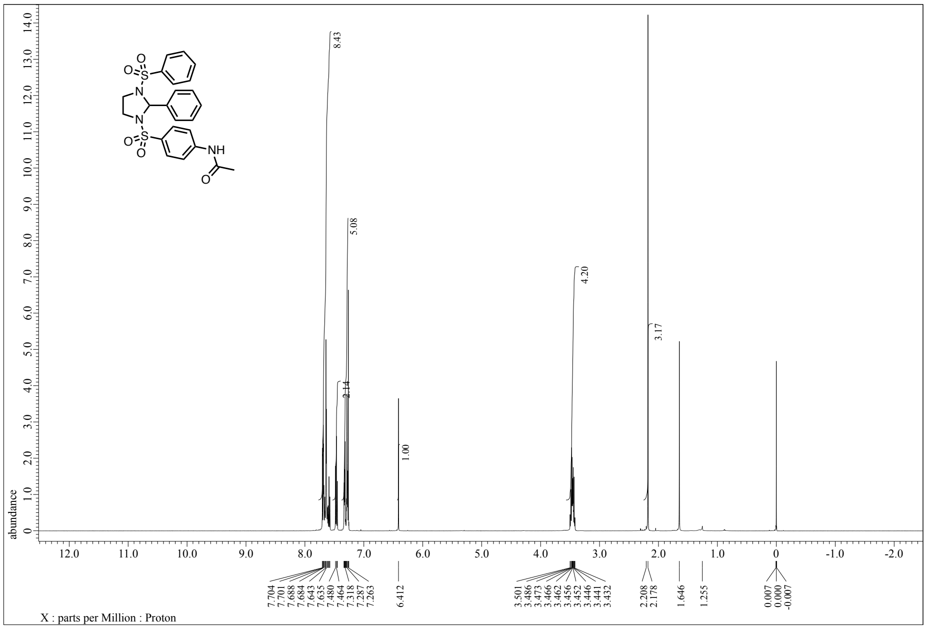

Supplementary Figure 60. ^{1}H NMR (500 MHz $CDCl_3$) spectrum of **3m**.

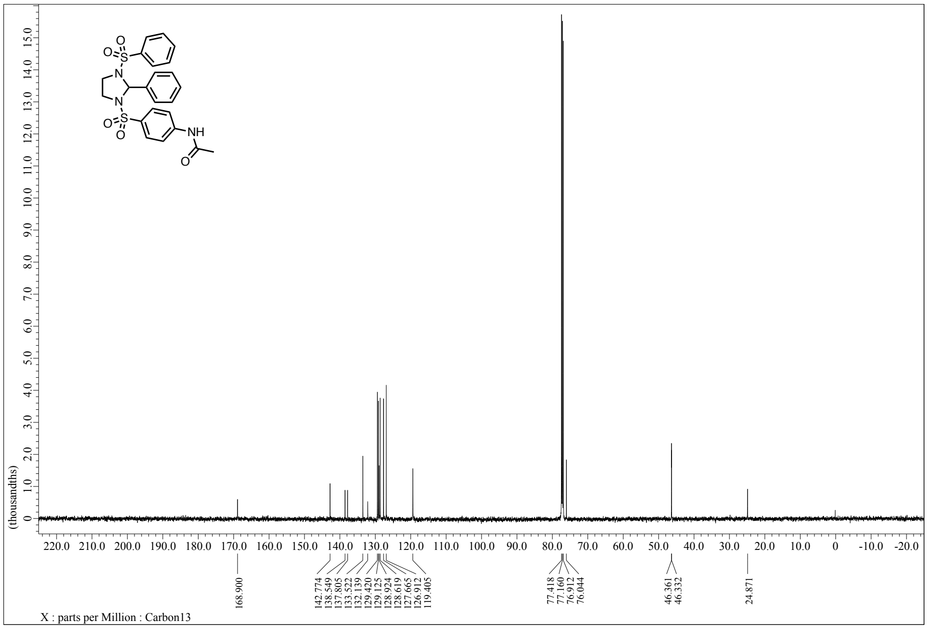

Supplementary Figure 61. ^{13}C NMR (126 MHz $CDCl_3$) spectrum of **3m**.

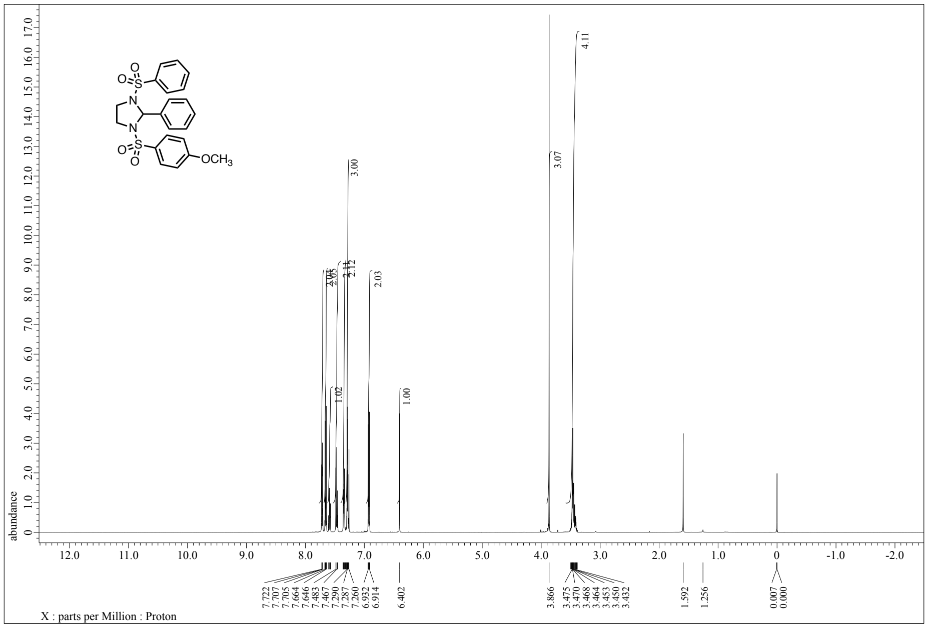

Supplementary Figure 62. ^{1}H NMR (500 MHz $CDCl_3$) spectrum of **3n**.

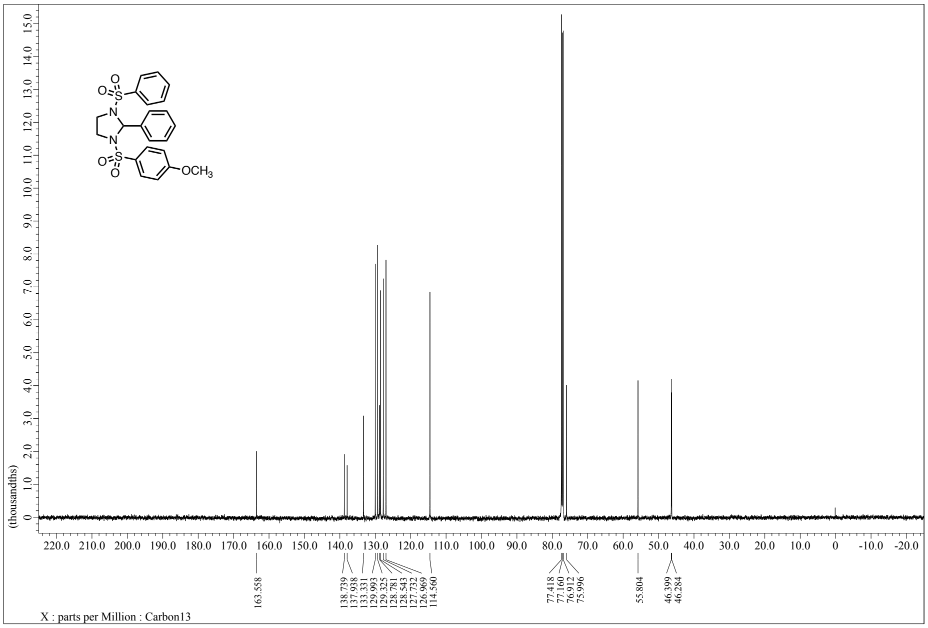

Supplementary Figure 63. ^{13}C NMR (126 MHz $CDCl_3$) spectrum of **3n**.

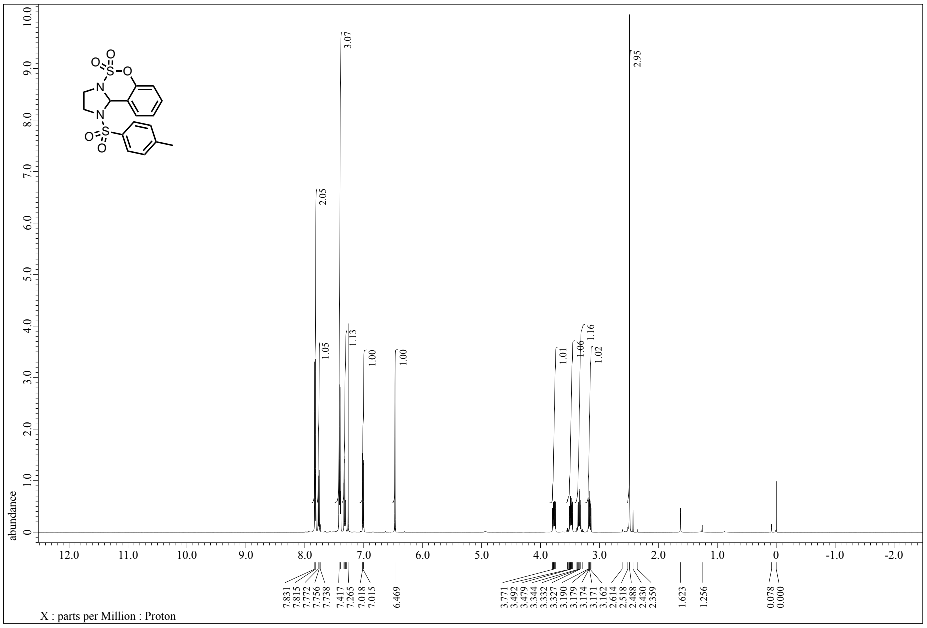

Supplementary Figure 64. ^{1}H NMR (500 MHz $CDCl_3$) spectrum of **3o**.

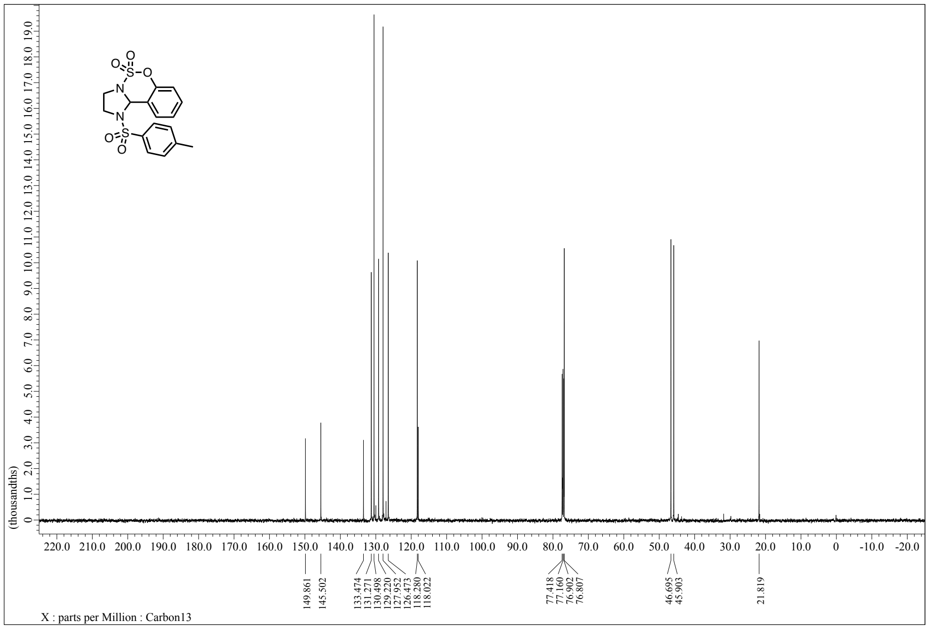

Supplementary Figure 65. ^{13}C NMR (126 MHz $CDCl_3$) spectrum of **3o**.

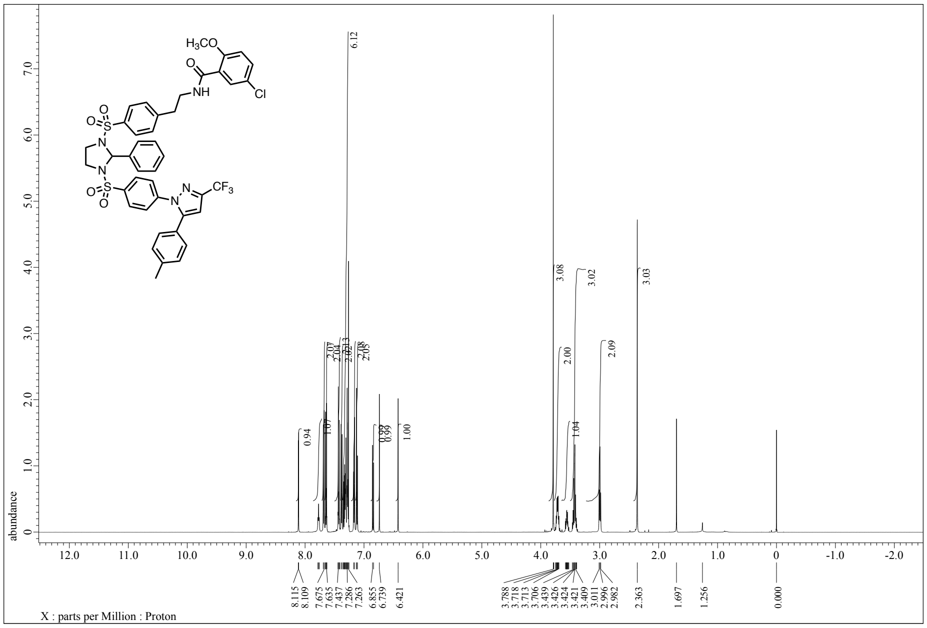

Supplementary Figure 66. ^{1}H NMR (500 MHz $CDCl_3$) spectrum of **3p**.

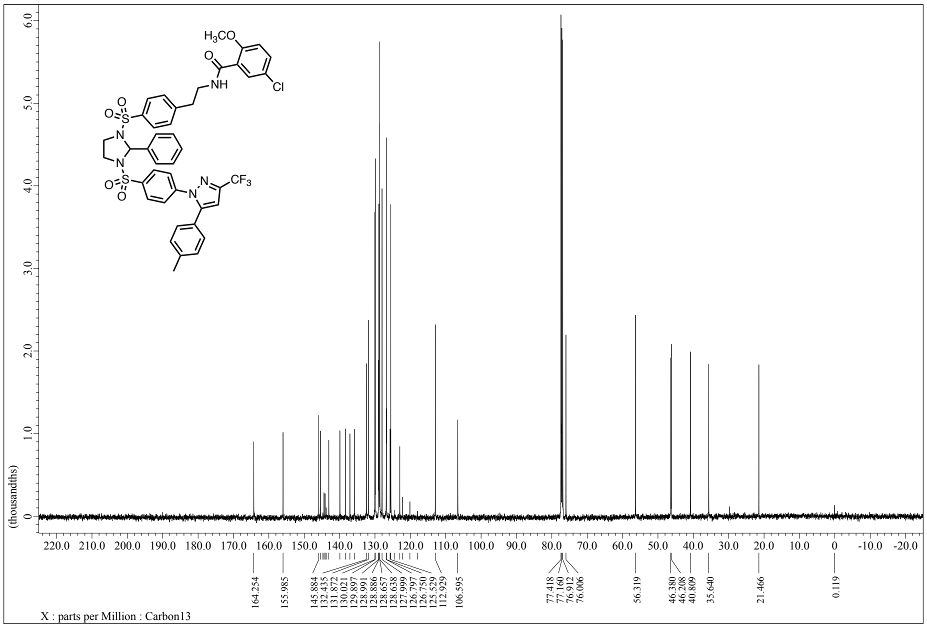

Supplementary Figure 67. ^{13}C NMR (126 MHz $CDCl_3$) spectrum of **3p**.

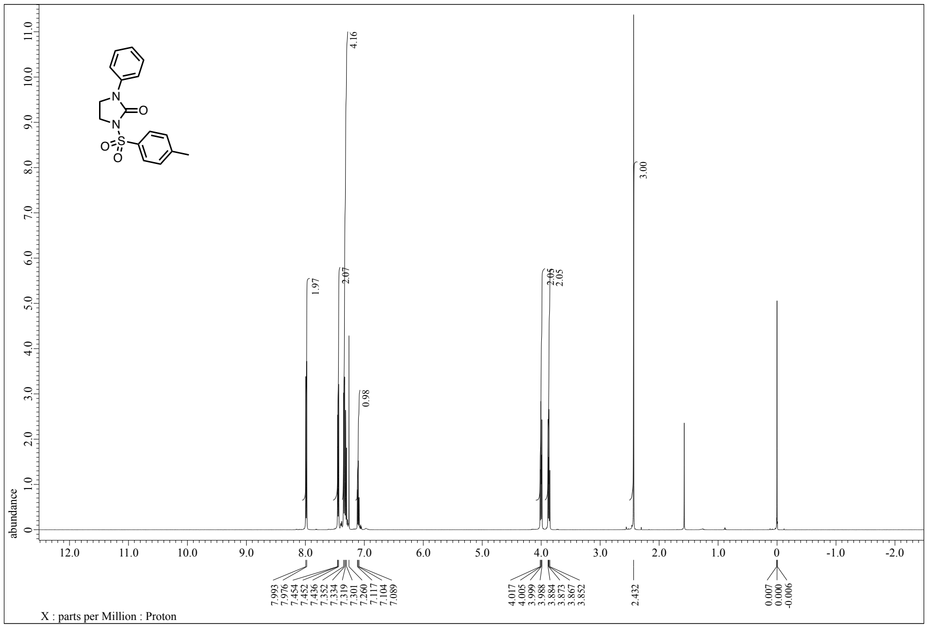

Supplementary Figure 68. ^{1}H NMR (500 MHz $CDCl_3$) spectrum of **5a**.

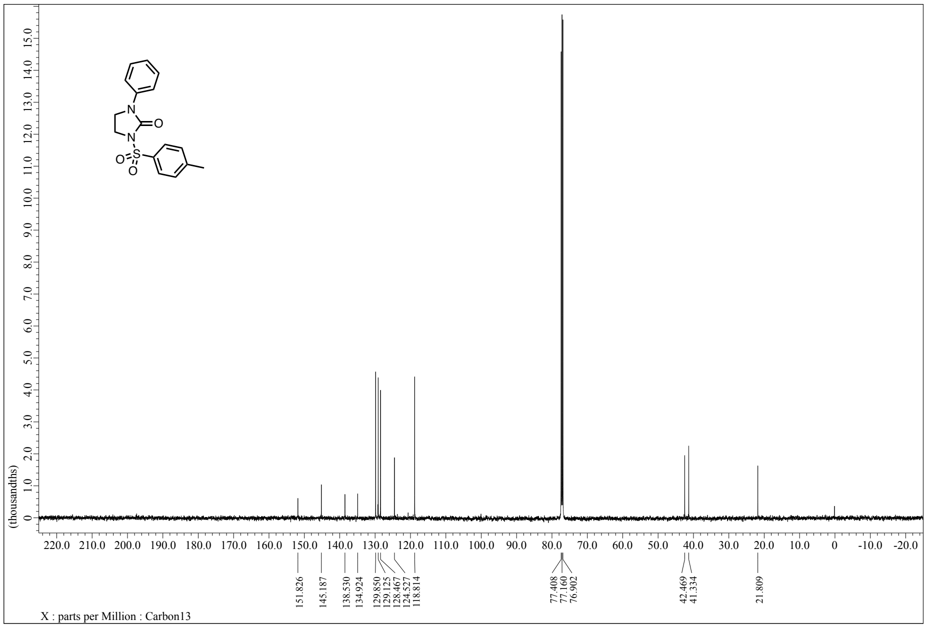

Supplementary Figure 69. ^{13}C NMR (126 MHz $CDCl_3$) spectrum of **5a**.

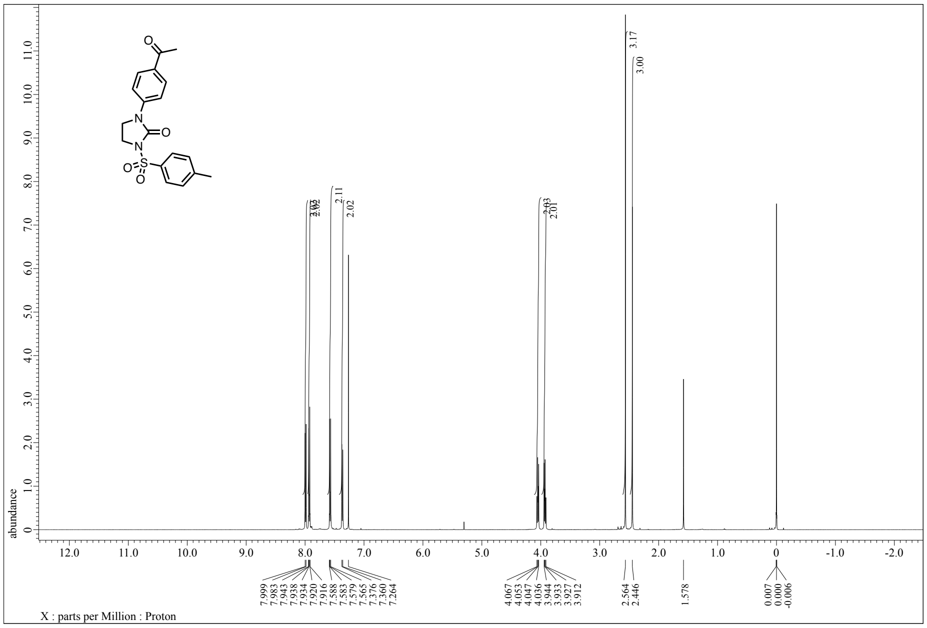

Supplementary Figure 70. ^{1}H NMR (500 MHz $CDCl_3$) spectrum of **5b**.

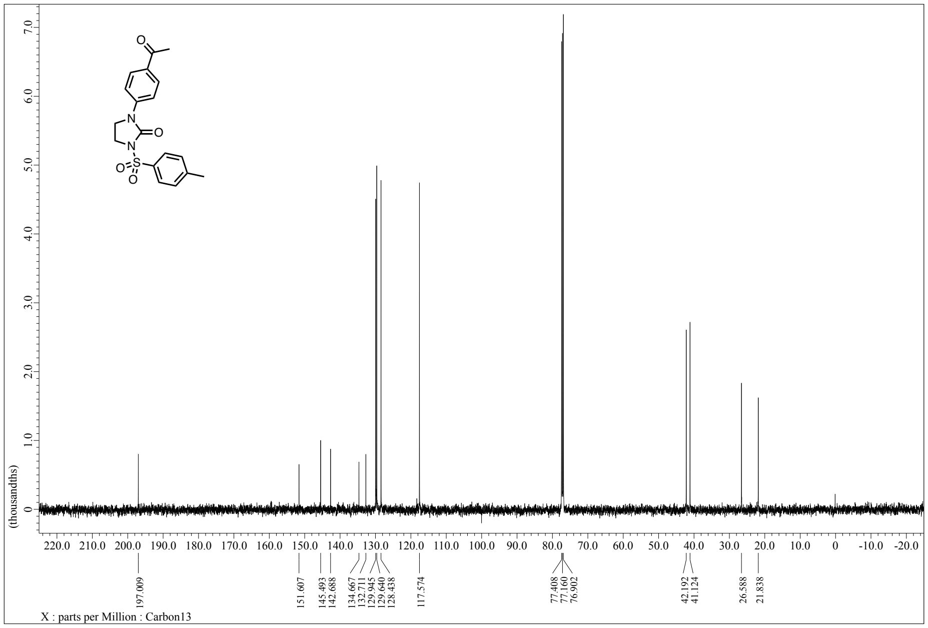

Supplementary Figure 71. ^{13}C NMR (126 MHz $CDCl_3$) spectrum of **5b**.

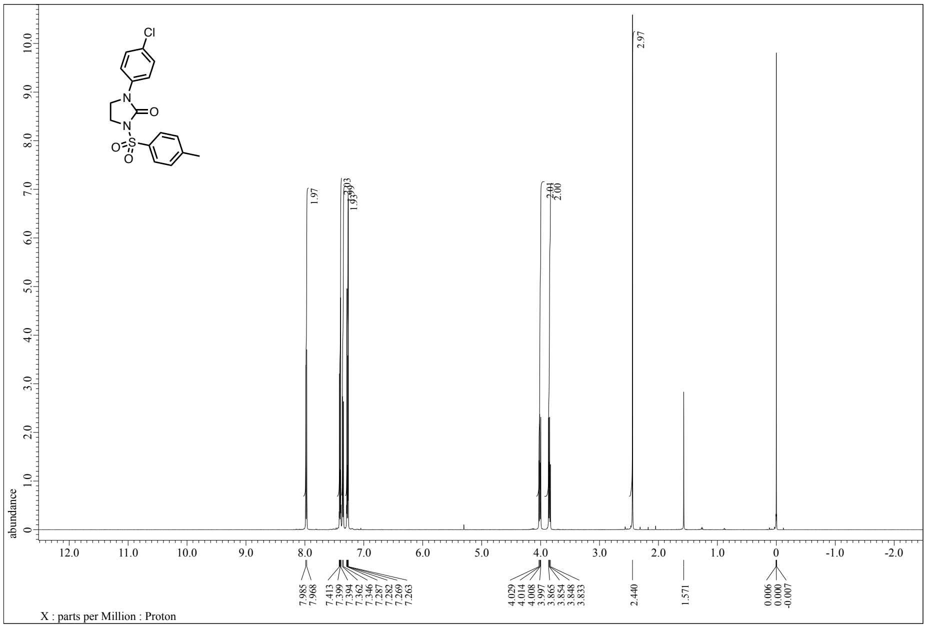

Supplementary Figure 72. ^{1}H NMR (500 MHz $CDCl_3$) spectrum of **5c**.

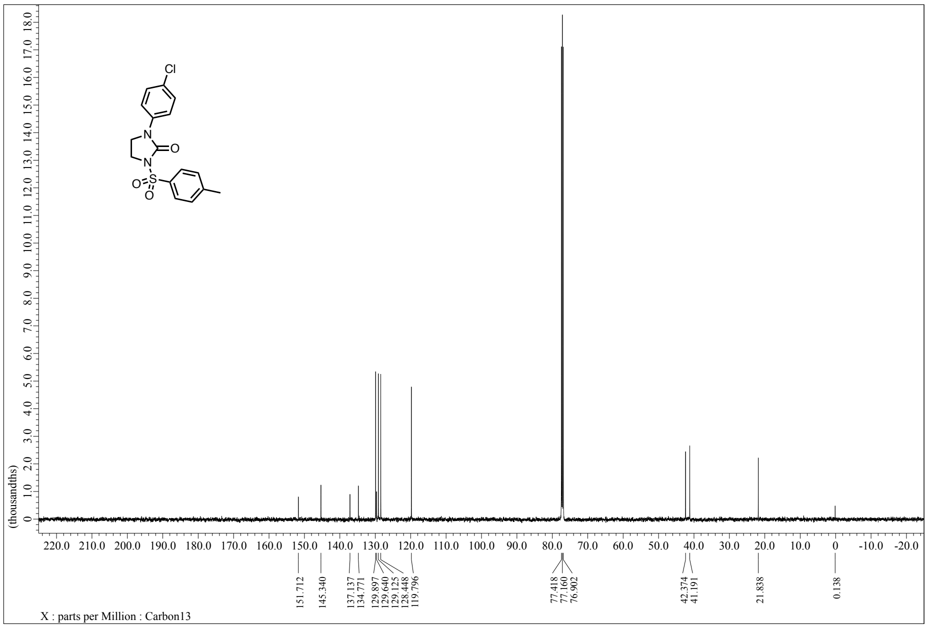

Supplementary Figure 73. ^{13}C NMR (126 MHz $CDCl_3$) spectrum of **5c**.

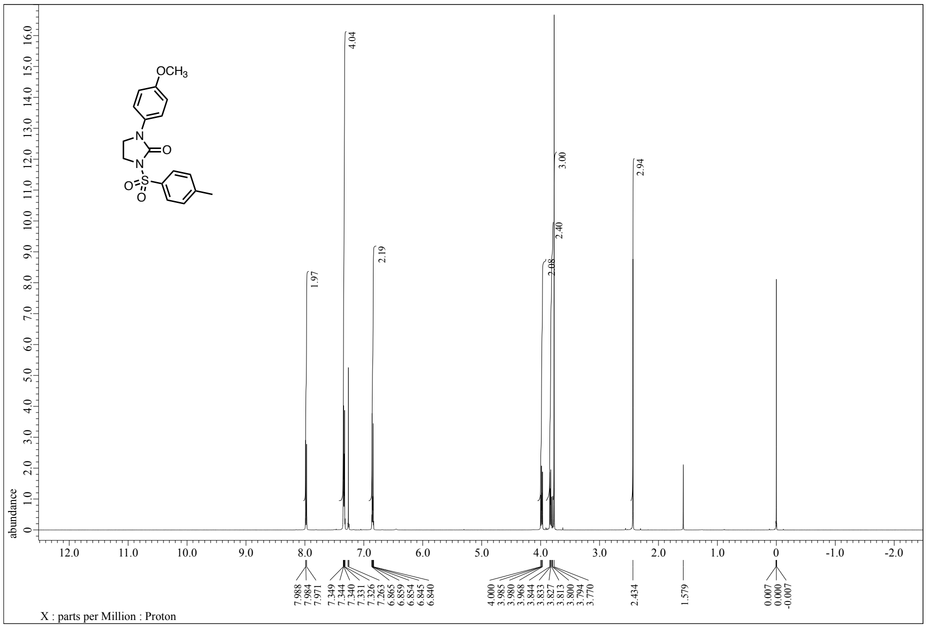

Supplementary Figure 74. ^{1}H NMR (500 MHz $CDCl_3$) spectrum of **5d**.

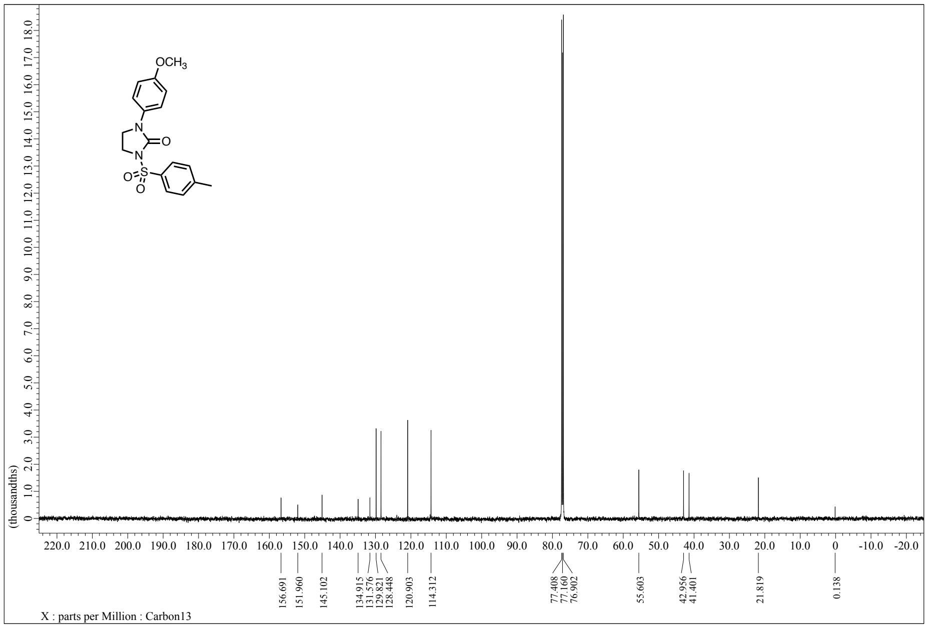

Supplementary Figure 75. ^{13}C NMR (126 MHz $CDCl_3$) spectrum of **5d**.

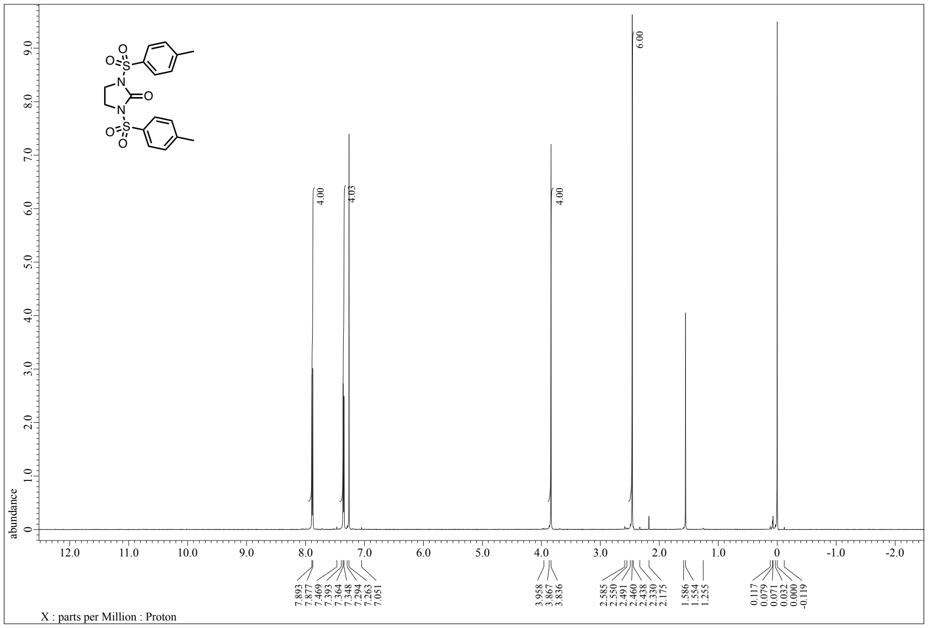

Supplementary Figure 76. ^{1}H NMR (500 MHz $CDCl_3$) spectrum of **5e**.

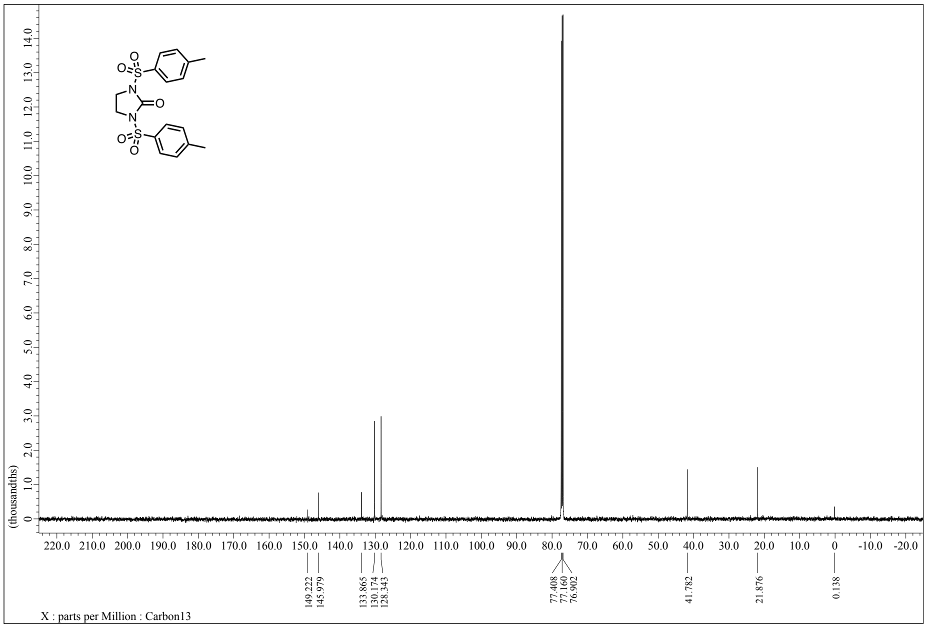

Supplementary Figure 77. ^{13}C NMR (126 MHz $CDCl_3$) spectrum of **5e**.

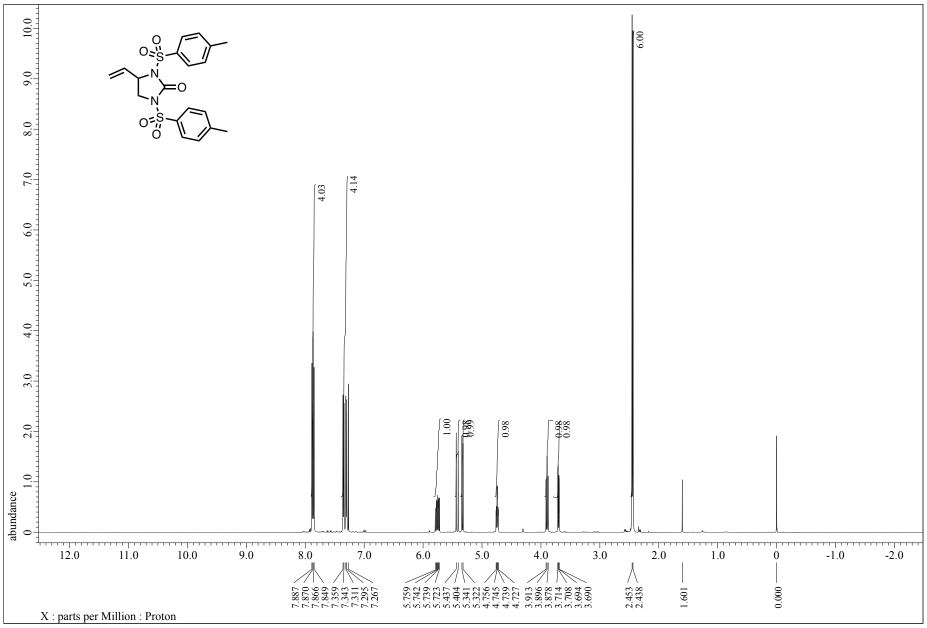

Supplementary Figure 78. ^{1}H NMR (500 MHz CDCl$_3$) spectrum of **5f**.

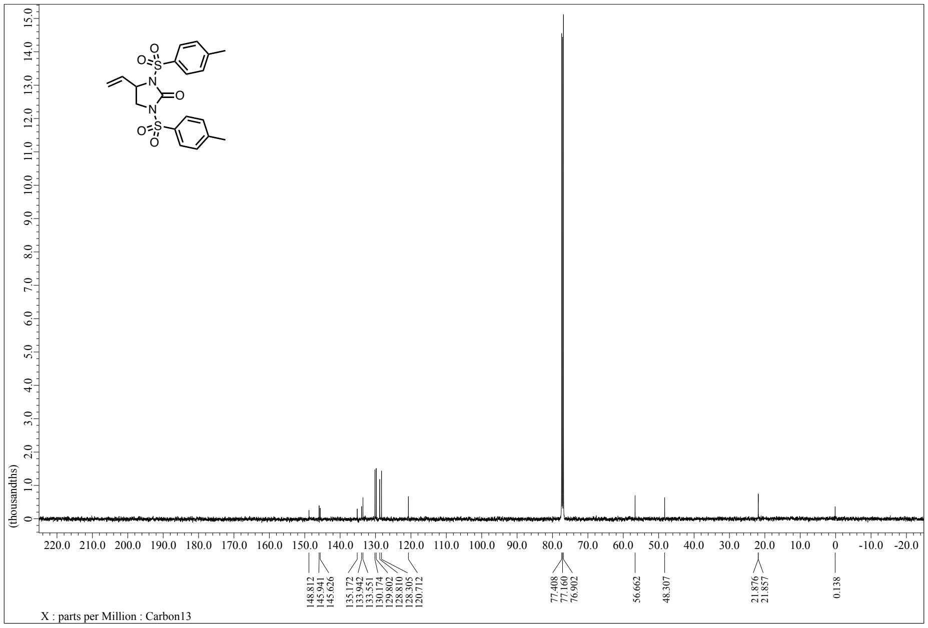

Supplementary Figure 79. ^{13}C NMR (126 MHz CDCl$_3$) spectrum of **5f**.

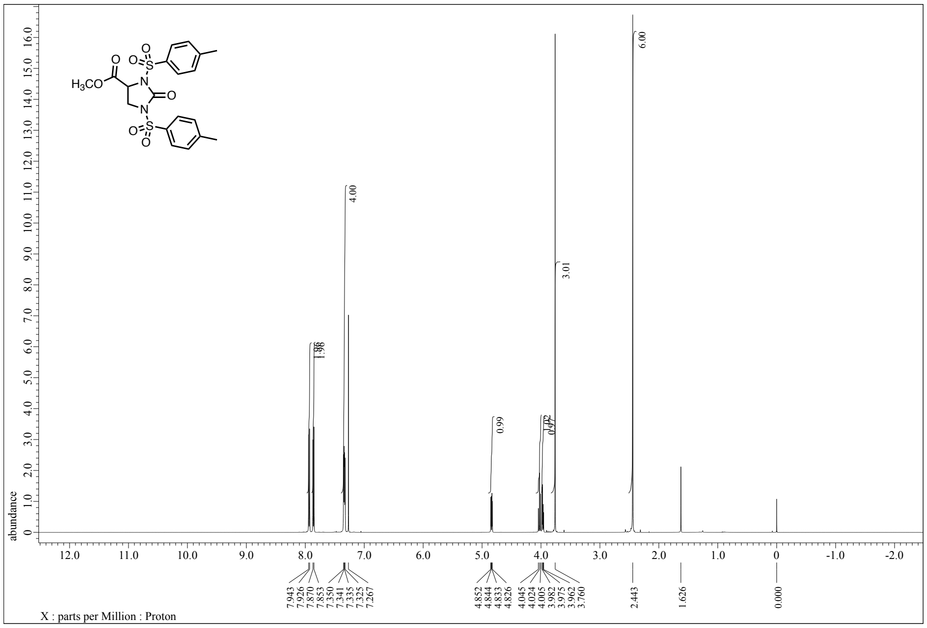

Supplementary Figure 80. ^{1}H NMR (500 MHz $CDCl_3$) spectrum of **5g**.

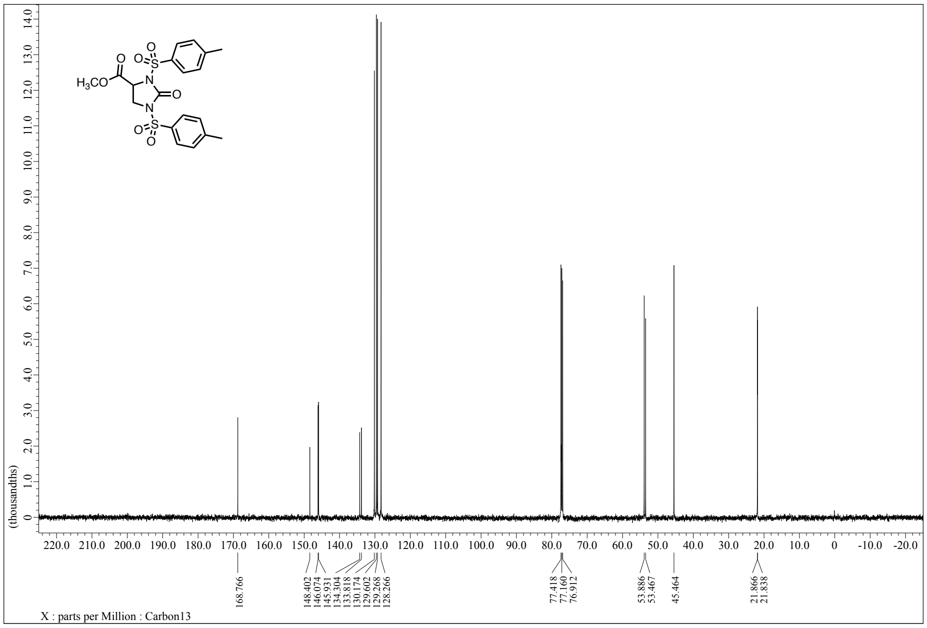

Supplementary Figure 81. ^{13}C NMR (126 MHz $CDCl_3$) spectrum of **5g**.

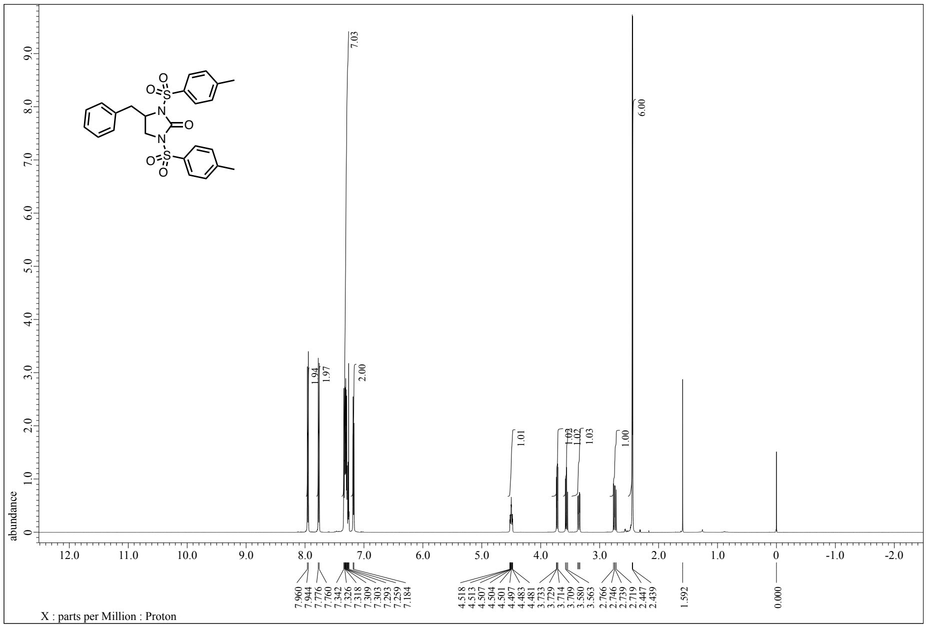

Supplementary Figure 82. ^{1}H NMR (500 MHz $CDCl_3$) spectrum of **5h**.

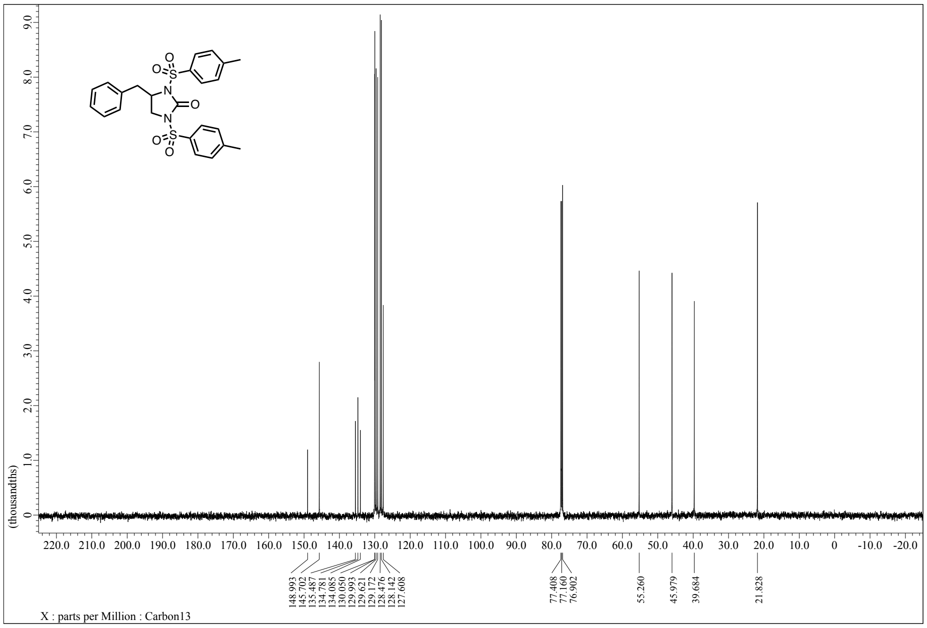

Supplementary Figure 83. ^{13}C NMR (126 MHz $CDCl_3$) spectrum of **5h**.

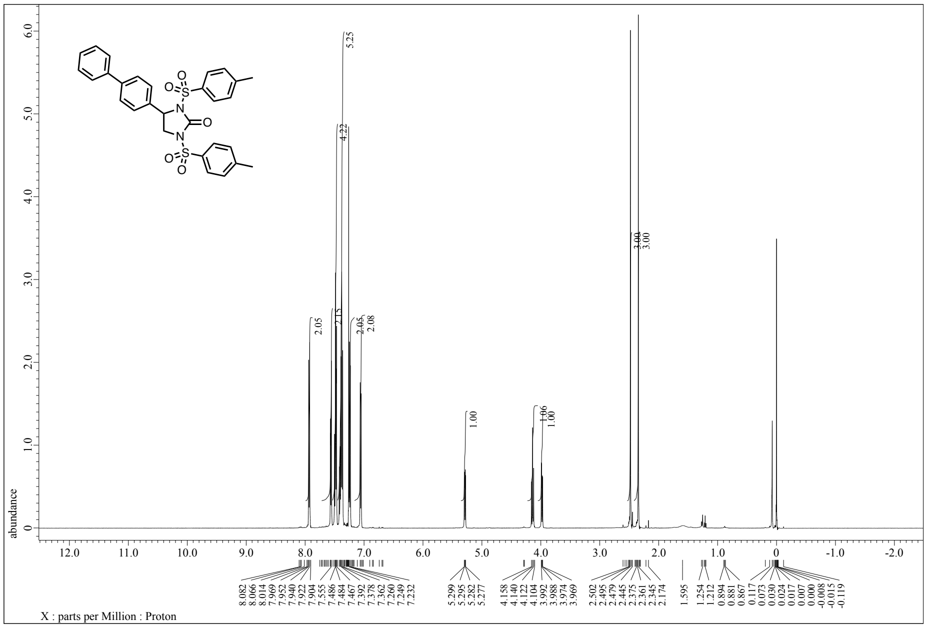

Supplementary Figure 84. ^{1}H NMR (500 MHz $CDCl_3$) spectrum of **5i**.

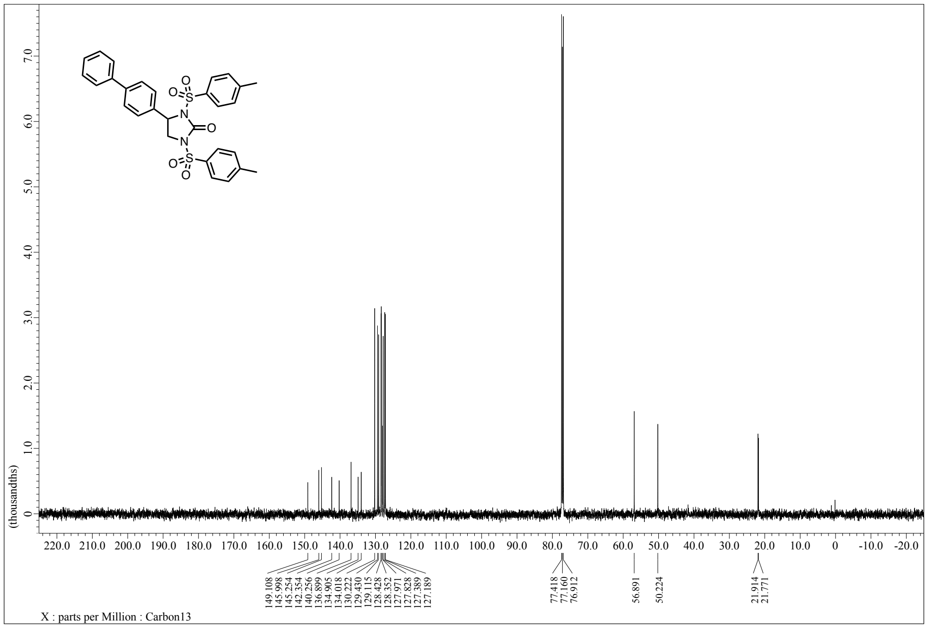

Supplementary Figure 85. ^{13}C NMR (126 MHz $CDCl_3$) spectrum of **5i**.

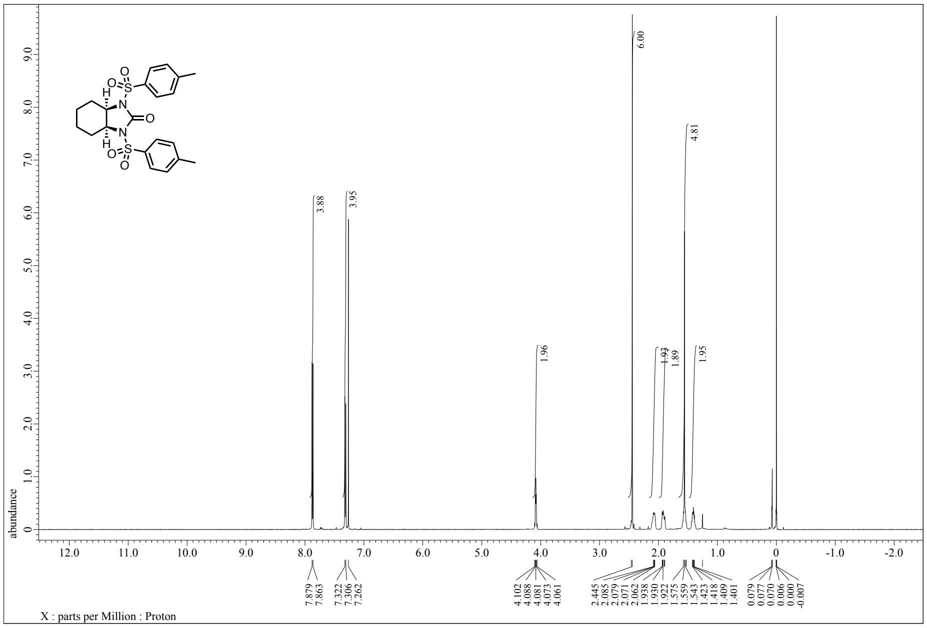

Supplementary Figure 86. ^{1}H NMR (500 MHz $CDCl_3$) spectrum of **5j**.

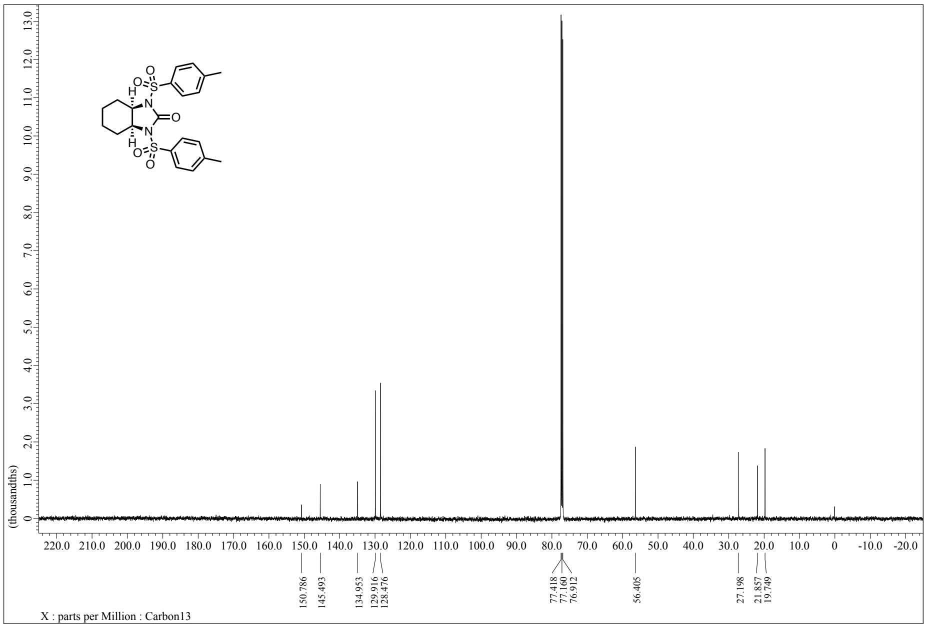

Supplementary Figure 87. ^{13}C NMR (126 MHz $CDCl_3$) spectrum of **5j**.

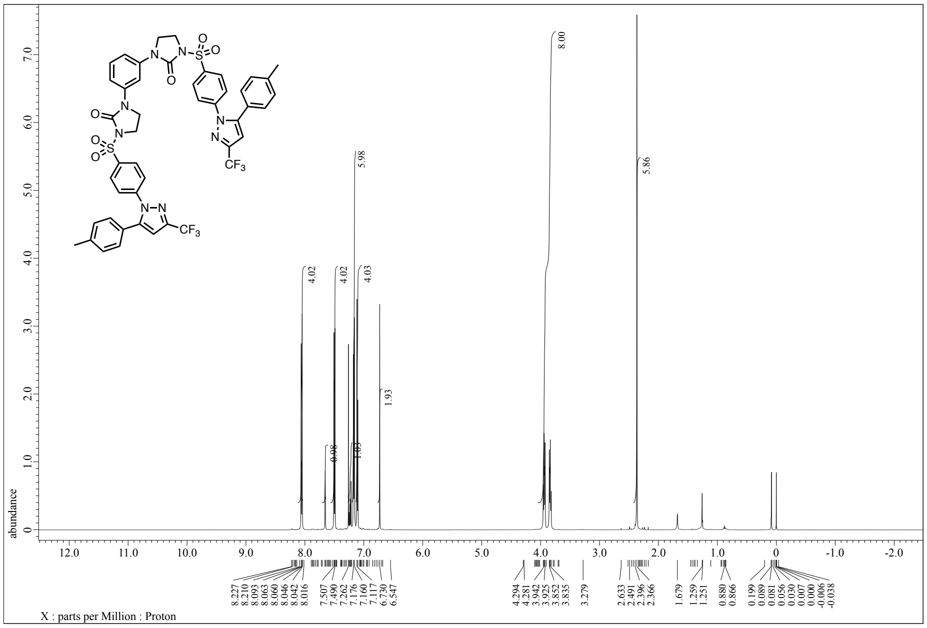

Supplementary Figure 88. ^{1}H NMR (500 MHz $CDCl_3$) spectrum of **5k**.

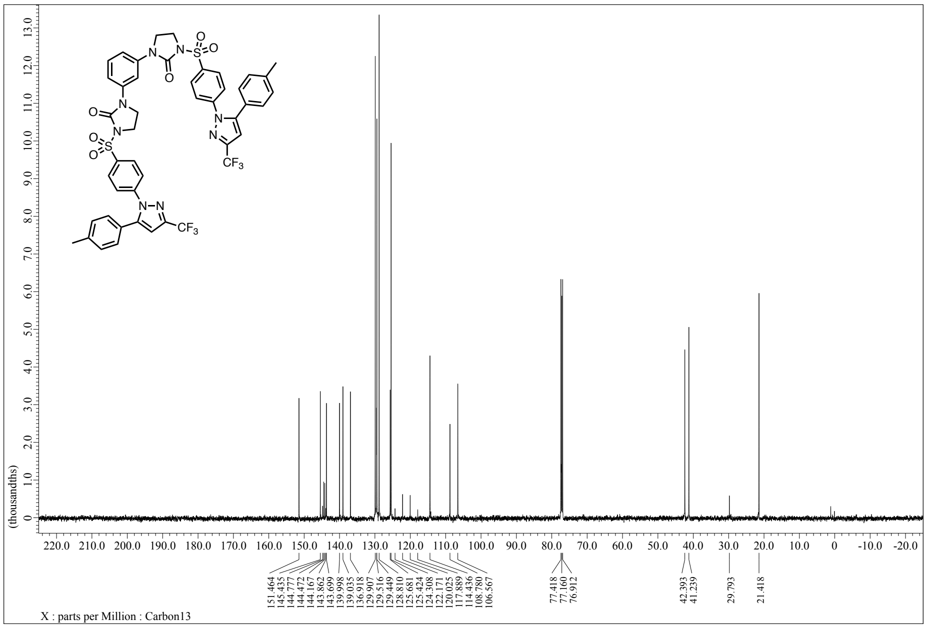

Supplementary Figure 89. ^{13}C NMR (126 MHz $CDCl_3$) spectrum of **5k**.
